# Chemically Recyclable Poly(β-thioether ester)s Based on Rigid Spirocyclic Ketal Diols Derived from Citric Acid

**DOI:** 10.1021/acs.biomac.2c00452

**Published:** 2022-05-26

**Authors:** Rauno Sedrik, Olivier Bonjour, Siim Laanesoo, Ilme Liblikas, Tõnis Pehk, Patric Jannasch, Lauri Vares

**Affiliations:** † Institute of Technology, University of Tartu, Nooruse 1, Tartu 50411, Estonia; ‡ Laboratory of Chemical Physics, National Institute of Chemical Physics and Biophysics, Akadeemia tee 23, Tallinn 12618, Estonia; § Department of Chemistry, Lund University, Box 124, Lund 221 00, Sweden

## Abstract

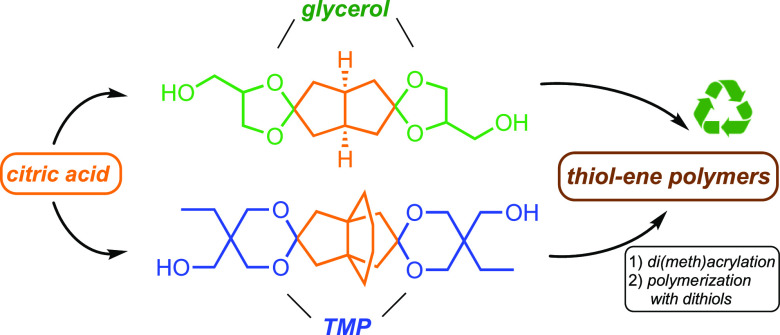

Incorporating rigid cyclic acetal and ketal units into polymer structures is an important strategy toward recyclable high-performance materials from renewable resources. In the present work, citric acid, a widely used platform chemical derived from biomass, has been efficiently converted into di- and tricyclic diketones. Ketalization with glycerol or trimethylolpropane afforded rigid spirodiols, which were obtained as complex mixtures of isomers. After a comprehensive NMR analysis, the spirodiols were converted into the respective di(meth)acrylates and utilized in thiol–ene polymerizations in combination with different dithiols. The resulting poly(β-thioether ester ketal)s were thermally stable up to 300 °C and showed glass-transition temperatures in a range of −7 to 40 °C, depending on monomer composition. The polymers were stable in aqueous acids and bases, but in a mixture of 1 M aqueous HCl and acetone, the ketal functional groups were cleanly hydrolyzed, opening the pathway for potential chemical recycling of these materials. We envision that these novel bioderived spirodiols have a great potential to become valuable and versatile bio-based building blocks for several different kinds of polymer materials.

## 1 Introduction

Replacing the fossil oil-derived materials we use in our everyday life with more sustainable and readily recyclable bio-based alternatives drives research and development in both the scientific community and industry, thereby reducing our environmental footprint.^1,2^ However, there are many challenges and pitfalls in the quest for competitive bio-based plastics. It is perhaps particularly difficult to combine processability and recyclability with the high demands for material properties required in various applications in an economically viable way.^3^ Hence, in 2017, the market share of bioderived plastics was only around 2%.^4^ Diols are one of the most versatile building blocks in the production of new bio-based polymers. In addition to the direct use as monomers in condensation polymerizations, these compounds can be converted into, for example, corresponding di(meth)acrylate, divinyl, diallyl, and diepoxide derivatives, which can be polymerized to form a wide variety of different materials.^5^ Rigid bioderived diol monomers are required for polymers with high glass-transition temperatures (*T*_g_’s). In this respect, bicyclic isosorbide is one of the most accessible ones and has therefore been extensively investigated in polymer science.^6^ It has shown great promise as a building block for the preparation of, e.g., (meth)acrylates,^7−9^ epoxides,^10,11^ and different condensation polymers.^6^ However, the poor thermal stability, in combination with the restricted reactivity of its secondary hydroxy groups, has limited its use.^12^

**Scheme 1:**
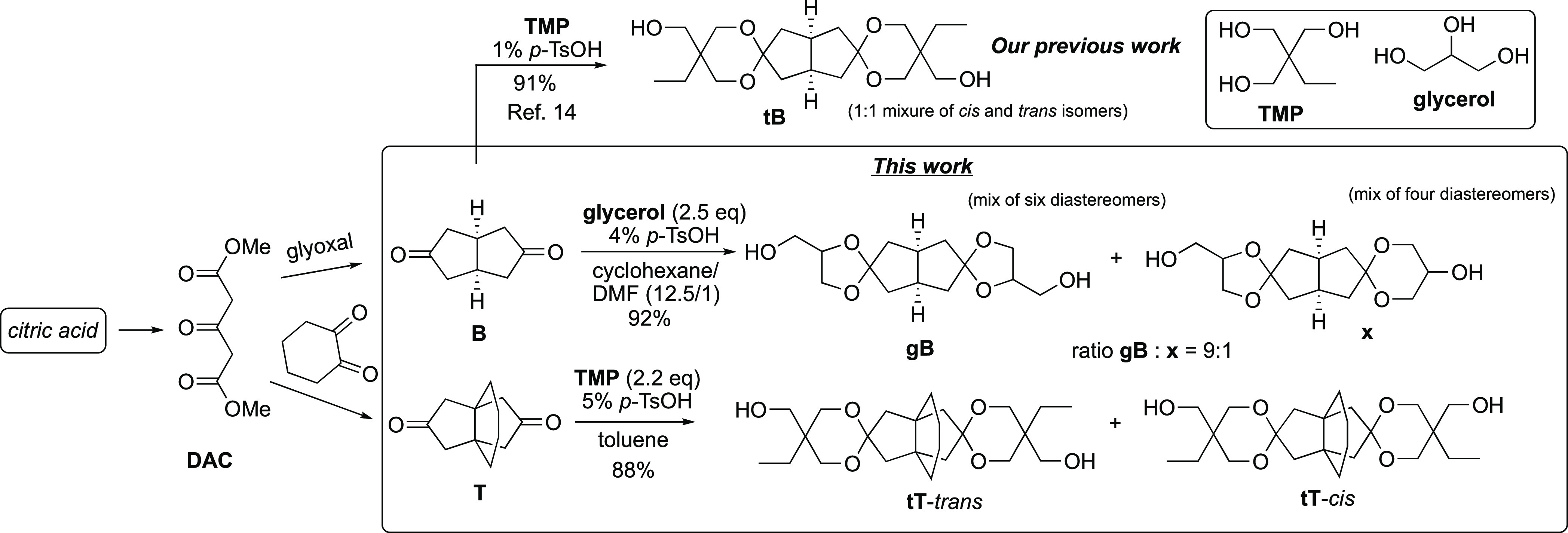
Synthesis of Spirocyclic Diols **tT** and **gB**

One attractive strategy to increase polymer rigidity is to introduce cyclic acetal and ketal units into the monomers. This can be achieved by reacting an aldehyde and ketone group, respectively, with a suitable diol in the presence of an acidic catalyst.^13^ The use of cyclic ketones leads to spiro-ketals, which are especially rigid structures. We have recently developed a spirodiol (**tB**, Scheme 1) obtained by reacting a bicyclic diketone derived from citric acid with partially bio-based trimethylolpropane (TMP).^14^ Polycarbonates prepared from **tB** showed high thermal stability, up to 350 °C, with essentially no discoloration after employing polycondensation temperatures up to 280 °C.

Aromatic spirodiols have been prepared by Mankar and co-workers by reacting 2 equiv of vanillin with pentaerythritol.^15^ Using a similar approach, Warlin et al. reported the reaction of pentaerythritol with 2 equiv of 5-(hydroxymethyl)furfural (5-HMF) to obtain a spirocyclic diol.^16^ de Vries, on the other hand, prepared a spirodiol by reacting 1 equiv of 5-HMF with glycerol.^17^ Glycerol has also been applied by other groups. For example, Du Prez et al. used 1,4-cyclohexadienone and 4,4′-dicyclohexanone,^18^ and Suh et al. employed camphorquinone,^19^ to prepare the corresponding glycerol diketals. Recently, a bifurane-based glycerol diacetal was reported by Kasuya et al.,^20^ and mannitol-based diketals with camphor have been disclosed by Suh’s research group.^21^ Acetals and ketals are generally stable under basic conditions but are susceptible to hydrolytic degradation via acidic hydrolysis.^22^ However, such an inherent instability can open a pathway for chemical recycling of acetal-containing polymers,^20,23^ or promote biodegradation as recently reported by Zhang and Zhu.^24^ The latter study demonstrated that the ester bond in spirodiacetal-containing polyesters can be enzymatically hydrolyzed after degradation of the diacetal functionality in a 2 M aqueous HCl solution. Several examples of potentially chemically recyclable acetal-containing polymers under acidic hydrolytic conditions have been demonstrated by Zhu,^25^ de Vries,^17^ and Miller et al.^26^

The thiol-Michael addition reaction can be described as the addition of a thiolate nucleophile to an α,β-unsaturated carbonyl in the presence of a basic or nucleophilic catalyst.^27^ This thermodynamically favored reaction has been used for more than 100 years in the synthesis of small molecules and has been accepted as a “click” reaction.^28^ Due to the operational simplicity, high regioselectivity, and the possibility to carry out reactions under mild conditions, it has in recent decades gained popularity also in material science, and particularly in the medical field.^27,29^ Acrylates, methacrylates, and acrylamides are often used as Michael acceptors, but vinyl sulfones, acrylonitriles, and other electron-deficient alkenes bearing a noncarbonyl electron-withdrawing group can also be utilized.^30^ Mild organobases such as Et_3_N and 1,8-diazabicyclo[5.4.0]undec-7-ene (DBU) or nucleophilic phosphines are commonly employed as catalysts. In addition to the catalyzed mechanism, thiol-Michael polymerizations can also proceed via a radical mechanism initiated by UV light, heat, or radical initiators such as AIBN.^31^ In the case of the base-catalyzed reaction, the rate increases with the electron deficiency of the alkene.^32^ In contrast, the radical reaction is typically favored by more electron-rich alkenes.^30^

Thiol-Michael polymerizations have been employed for the synthesis of various cross-linked, star-shaped, hyperbranched, and linear polymers.^28^ The use of bio-based building blocks has also been reported. For example, isosorbide diacrylate copolymers with linear dithiols were prepared by Long et al., reaching *T*_g_ values from −14 to 15 °C.^33^ Various sugar-based poly(ester-thioethers) with *T*_g_ values from −8 to 19 °C were reported by Reineke et al.^34^ In addition, soybean oil has been used as a starting material for cross-linked thiol–ene networks.^35−37^ Biodegradable cross-linked thiol–ene polymers with *T*_g_ values ranging from −60 to −30 °C have been developed by Junkers et al.^38^ Both acrylate and thiol end groups are potentially reactive and offer different possibilities for postmodification. For example, terminal acrylates are susceptible to cross-linking reactions. Hence, the use of slight excess of the dithiols reduces the number of acrylate end groups and thereby decreases the risk of cross-linking such polymers.^39^ In addition, the reversibility of thiol–ene reaction has been demonstrated.^40^

In the present work, we have synthesized two new readily accessible rigid spirocyclic diols derived from citric acid. We envision that these spirocyclic diols have the potential to become valuable bio-based building blocks in various applications where stiff diols or derivatives are required. The diols were then converted into the corresponding acrylate and methacrylate diesters and investigated in copolymerizations with different dithiols via base-catalyzed thiol-Michael “click” reactions. We were particularly interested in studying the relationship between the di(meth)acrylate structure and the properties of the corresponding polymers. The poly(β-thioether ester ketal)s were characterized by nuclear magnetic resonance (NMR) spectroscopy, size exclusion chromatography (SEC), thermogravimetry analysis (TGA), calorimetry (DSC), and dynamic mechanical analysis (DMA). The hydrolytic stability and cleavage of the ketal functionalities were studied for both the monomers and the polymers to investigate the potential for the chemical recycling of these materials.

## 2 Experimental Section

### 2.1 Materials

Dimethyl-1,3-acetonedicarboxylate (DAC, 97%) and trimethylolpropane (TMP, 98%) were obtained from Acros Organics; 3,4-hexanedione (94%), acryloyl chloride (96%), methacryloyl chloride (97%), propane-1,3-dithiol (PDT, 97%), and hexane-1,6-dithiol (HDT, 97%) were obtained from Alfa Aesar; 4,4′-thiobisbenzenethiol (TBBT, 98%) was obtained from Sigma-Aldrich. All reagents and solvents were used as received. Hydroquinone was employed to stabilize the **tBa** and **gBa** monomers after synthesis. The syntheses of the monomers were monitored by thin-layer chromatography (TLC, Xtra SIL G/UV_254_), and the plates were visualized by staining with a phosphomolybdic acid solution. For flash chromatography, silica gel 60 (0.040–0.063 mm, 230–400 mesh) was used.

### 2.2 Structural Characterization

The structures of the monomers and polymers were characterized by NMR spectroscopy using 800 and 400 MHz Bruker spectrometers. The samples were measured in chloroform-*d* and dimethyl sulfoxide-*d*_6_. Acetone-*d*_6_ and acetonitrile-*d*_3_ were used for hydrolysis experiments. The ^1^H and ^13^C spectra were recorded at 800 or 400 MHz, and 201 or 101 MHz, respectively. Residual solvent signals were used for calibration (7.26 and 77.00 ppm for C*D*Cl_3_, 2.50 ppm for DMSO-*d*_6_, 2.05 ppm for acetone-*d*_6_, and 1.94 ppm for acetonitrile-*d*_3_). High-resolution mass spectrometry (HRMS) analyses of the novel monomers were carried out using a Thermo Electron LTQ Orbitrap XL analyzer.

The molar masses of the polymers were determined by size exclusion chromatography (SEC) using either CHCl_3_ or THF as eluent. A Shimadzu Prominence setup with a refractive index detector (RID-20A) and three Shodex columns (KF-805, -804, and -802.5, coupled in series) was used. All samples were run at 40 °C at an elution rate of 1 mL/min. Poly(ethylene oxide) standards (*M*_n_ = 3860, 21 160, 49 640, 96 100 g/mol) were employed for calibration, and the results were analyzed using the Shimadzu LabSolution software.

### 2.3 Synthesis of Spirodiols

#### 2.3.1 Glycerol-Spirodiol **gB**

Diketone **B** (5.99 g, 43.4 mmol), glycerol (11.18 g, 121.6 mmol, 2.8 equiv), and *p*-toluenesulfonic acid monohydrate (0.41 g, 2.17 mmol, 0.05 equiv) were weighed into a 250 mL round-bottom flask. Cyclohexane (150 mL) and dimethylformamide (DMF, 12 mL) were added, and the mixture was refluxed for 15 h using a Dean–Stark apparatus for water removal. After cooling, the crude reaction mixture was concentrated under reduced pressure on a rotavapor. The residue was purified by flash chromatography (gradual elution: petroleum ether/EtOAc, 1:1, and later 10% MeOH in EtOAc) to give 11.43 g (92%) of **gB** as a colorless viscous liquid. Alternatively, an elution mixture of 5% MeOH in CH_2_Cl_2_ could be employed for flash chromatography. The product contained traces of DMF (ca 2 mol %), which were difficult to remove by a single flash column.

##### 2.3.1.1 Alternative Procedure without DMF

Diketone **B** (2.76 g, 20 mmol), glycerol (4.42 g, 48 mmol, 2.4 equiv), and *p*-toluenesulfonic acid monohydrate (0.09 g, 0.47 mmol, 0.023 equiv) were weighed into a 250 mL round-bottom flask. Cyclohexane (80 mL) and toluene (40 mL) were added, and the mixture was refluxed for 16 h using a Dean–Stark apparatus to remove the water. After cooling, the crude reaction mixture was concentrated under reduced pressure on a rotavapor and purified as described above. The diol was isolated in 82% yield (4.69 g). A thorough inspection of the NMR spectra indicated the presence of six isomeric products resulting from the different mutual orientations of the terminal hydroxymethyl groups. In addition, ca. 10% of a regioisomeric product where one five-membered dioxalane ring had been replaced by a six-membered dioxane ring, as well as ca. 1% of a diol with two dioxane rings, were also detected. The diol with five- and six-membered spiro rings consisted of four diastereomers, whereas the diol with two six-membered rings consisted of two diastereomers. The detailed characterization and analytical data of isomers are given in the Supporting Information and discussed in Section 3. The isomers were not separable by conventional flash purification, and an isomeric mixture of **gB** was used in the subsequent step. *R*_f_ = 0.275 (100% EtOAc), *R*_f_ = 0.23 (4% MeOH/CH_2_Cl_2_), HRMS (ESI): calcd for C_14_H_22_O_6_ [M + Na]^+^ 309.1309, found 309.1312.

#### 2.3.2 Spirodiol tT

The tricyclic diketone **T** (1.026 g, 5.2 mmol, prepared according to the previously reported procedure,^41^ see the Supporting Information), TMP (1.537 g, 11.4 mmol, 2.2 equiv), and *p*-toluenesulfonic acid (40 mg, 0.23 mmol, 0.05 equiv) were added to a 100 mL round-bottom flask. Toluene (40 mL) was added, and the flask was fitted with a Dean–Stark apparatus before refluxing for 48 h. After the reaction mixture had cooled, the organic layers were combined, dried over MgSO_4_, and concentrated under reduced pressure. The pure crystalline **tT** (roughly a 1:1 mixture of **tT**-*trans* and **tT**-*cis*) was obtained by crystallization in acetone/petroleum ether (1:1) with a yield of 30%. Alternatively, the crude product was purified by flash chromatography (2% methanol in CH_2_Cl_2_) to afford 1.945 g (88%) of the crystalline **tT** (mixture of isomers). The isomers could be partially separated for analytical purposes by chromatography, but no attempts for full separation were made and the mixture of **tT**-*cis* and **tT**-*trans* was used in the next step. TLC: *R*_f_ = 0.33 (5% MeOH in CH_2_Cl_2_). NMR data of *cis* and *trans* isomers are given in the Supporting Information (Figures S10 and S11). HRMS (ESI): calculated for C_24_H_40_O_6_ [M + H]^+^ 425.2903, found 425.2898.

### 2.4 Di(meth)acrylate Monomer Synthesis

The spirodiol (1 equiv) was dissolved in CH_2_Cl_2_ or 2-MeTHF (0.1 g/mL) in a round-bottom flask flushed with argon and capped with a rubber septum. The mixture was cooled by an ice bath, and acryloyl- or methacryloyl chloride (2.2–2.5 equiv) and Et_3_N (2.5–3 equiv) were simultaneously added dropwise. After the addition, the ice bath was removed and the mixture was stirred overnight (16 h) at room temperature. Next, the reaction mixture was quenched using a saturated aqueous NaHCO_3_ solution and extracted with CH_2_Cl_2_ three times. The organic layers were gathered, dried over MgSO_4_, and concentrated under reduced pressure. The concentrate was then purified via flash chromatography and concentrated under vacuum to obtain the pure di(meth)acrylate product as a viscous liquid (for detailed procedures, see the Supporting Information).

### 2.5 Thiol-Michael Polymerization Reactions

We followed the procedure described by Long after slight modification.^33^ Di(meth)acrylate (typically about 500 mg) was dissolved in CHCl_3_ or 2-MeTHF to reach a concentration of ca. 100 mg/mL before 1 equiv of dithiol monomer was added. The di(meth)acrylates typically contained 5–10 wt % of residual solvents (EtOAc or CH_2_Cl_2_), which were difficult to remove due to the high viscosity of the monomers. The residual solvent content was estimated by ^1^H NMR analysis prior to the reaction and was taken into account in the calculations. The solution was cooled using an ice bath, and 0.1 equiv of DBU was added as a solution in chloroform. The ice bath was removed shortly afterward, and the solution was stirred at room temperature for 24–48 h. The progress of the polymerizations was monitored by NMR spectroscopy by comparing the remaining signals from the acrylate groups to those of the formed polymer. Due to signal overlap, the −S*H* signal was not detectable in the solution. The polymer was precipitated in 100 mL of MeOH. The crude product was stirred slowly overnight (16 h), after which the polymer precipitated. The solvent was decanted, and the polymer product was left to dry for 5–10 min, where after a small amount of CH_2_Cl_2_ (2–3 mL) was added to dissolve the polymer. A film of the polymer was cast in a small Petri dish and left to dry at room temperature overnight before it was removed from the dish for further drying under vacuum at 80 °C. Poly(β-thioether ester)s were generally soluble in THF, CHCl_3_, and toluene but did not dissolve in water, MeOH, CH_3_CN, DMSO, and Et_2_O. EtOAc only dissolved some of the polymers (see Table S2 for details).

### 2.6 Thermal Characterization

Thermogravimetric analysis (TGA) was performed using a TA Instruments TGA Q500 apparatus to determine the thermal stability of the polymers under an N_2_ flux of 60 mL/min. Samples of 2–12 mg were kept isothermally at 150 °C for up to 60 min to remove solvent residues. After equilibration at 40 °C, the samples were analyzed up to 600 °C at a heating rate of 10 °C/min. The thermal decomposition temperature (*T*_d,95_) was determined at a 5% weight loss. Differential scanning calorimetry (DSC) analysis was carried out using a TA Instruments DSC Q2000 differential scanning calorimeter. Dried samples of 3–9.5 mg were transferred to aluminum pans, which were hermetically sealed. In a preliminary study, the samples were first heated to 200 °C at a rate of 10 °C/min. This was followed by an isothermal period of 5 min before cooling to −50 °C and a 5 min isothermal period. Finally, the samples were heated to the original starting temperature at 10 °C/min. The *T*_g_’s were evaluated from the thermograms as the middle point between the onset and offset temperatures. Because of sample degradation, the maximum temperature was restricted to 100 °C in the subsequent measurements.

### 2.7 Dynamic Mechanical Analysis

Dynamic mechanical analysis (DMA) was carried out on a TA Instruments DMA Q800. Sample bars of 35 × 5 × 1 mm^3^ were hot-pressed between Teflon plates using a hydraulic press (Specac, GS15011) at 80–100 °C. Subsequently, the sample bars were analyzed at a frequency of 1 Hz and 0.1% strain between −50 and 100 °C at a heating rate of 3 °C/min. *T*_g_’s were determined from the local maximum value of the loss modulus.

### 2.8 Hydrolytic Stability and Degradation of Polymers

Initially, the polymers were submerged into aqueous solutions at pH = 0 (1 M HCl), 3 (citrate buffer), 8 (phosphate buffer), and 14 (1 M NaOH), respectively, and kept for 2 weeks at 37 °C. Next, a mixture of acetic acid and water at a 1:1 volume ratio^42^ was used for hydrolysis experiments at 90 °C. The third batch of polymer samples was submerged in a mixture of 10% 0.1 M aqueous HCl in 90% acetone and kept at 50 °C for 72 h. The final hydrolysis experiment was performed in a mixture of 10% 1 M aqueous HCl and 90% acetone. All of the submerged polymer samples dissolved after a few hours at 50 °C. The solvent was removed, and the residues were analyzed by TLC, SEC, and NMR spectroscopy.

## 3 Results and Discussion

The new diols **gB** and **tT** (Scheme 1) were prepared via ketalization of diketones **B** and **T**, respectively, using either glycerol or TMP triol. Glycerol is a byproduct of biodiesel production and is widely available at a very low cost.^43^ TMP, on the other hand, is a common building block employed in the polymer industry, and is also available with a 50% renewable carbon content.^44^ The spirodiols were further converted into the corresponding diacrylates and dimethacrylates, which were subsequently copolymerized with a series of linear dithiols via base-catalyzed thiol-Michael reactions to yield poly(β-thioether ester ketal)s.

### 3.1 Synthesis and Characterization of Spirodiols and Monomers

The synthetic strategy to the cyclic diketones **B** and **T** follows previously reported procedures^41,45,46^ and involves a Weiss–Cook condensation of dimethyl-1,3-acetonedicarboxylate (DAC) with either glyoxal or cyclohexane-1,2-dione, and a subsequent decarboxylation step. DAC can be obtained from citric acid via a well-established decarboxylation and esterification process,^47^ whereas cyclohexane-1,2-dione is prepared via oxidation of cyclohexanone,^48^ a widely used intermediate in the synthesis of nylon. In addition, a potential bio-based route to cyclohexane-1,2-dione has been recently reported.^49^

In the case of **T**, two slightly different procedures have been reported in the literature. In both cases, a phosphate buffer solution (pH 5.6) was used in the Weiss–Cook condensation, after which the solids were either collected and used in next step without purification (Cook et al.^45^) or washed with brine (Torres-Gómez et al.^41^). The latter alternative afforded a higher yield of the intermediate compound. After decarboxylation, the acidic reaction mixture was neutralized, followed by extraction with either ethyl acetate (Torres-Gómez^41^) or CH_2_Cl_2_ (Cook^45^). Finally, the pure diketone **T** was obtained by crystallization from methanol. We used a combination of these two methods; after the decarboxylation, we extracted the crude mixture prior to neutralizing the organic phase, hence avoiding the neutralization of a large volume of aqueous acidic solution.

Initially, we studied the ketalization reaction between diketone **B** and glycerol in the presence of a catalytic amount of *p*-TsOH (1–5 mol %) as an acidic catalyst. Water is a byproduct in this reaction and must be continuously removed to shift the equilibrium toward the desired ketal. Refluxing diketone **B** with 3.6 equiv of glycerol in the presence of *p*-TsOH (4%) in toluene for 5 h afforded diol **gB** in 54% yield (see Table S1 for details). In parallel to the incomplete conversion, a substantial amount of unidentified dark byproducts also formed under these conditions. Attempts to increase the conversion by increasing the reaction time did not improve the yield of the target diol but instead favored the formation of the dark byproducts. We speculated that the toluene reflux temperature was too high for this compound, and thus we replaced toluene with the lower-boiling cyclohexane. Reflux in cyclohexane resulted in lesser amounts of the unidentified dark byproducts, but the conversion was still incomplete after 18 h (2.6 equiv glycerol was used, **gB** isolated yield 50%). Increasing the amount of glycerol to 4 equiv did not significantly improve the yield. Next, we added a small amount of DMF to the reaction mixture to improve the solubility of the reagents and slightly increased the refluxing temperature. Reflux during 15 h in cyclohexane/DMF (12.5:1, v:v) resulted in the full consumption of the diketone, and the desired diol was isolated in 92% yield. Under these conditions, only minor amounts of the dark byproducts were detected. However, because the complete removal of DMF was tedious and due to the toxicity issues of this solvent,^50^ we instead selected an alternative 2:1 mixture of cyclohexane and toluene (v:v), which afforded the target diol **gB** as an oily substance in 82% yield. Aqueous extraction of the crude product prior to chromatography was avoided since the rather polar **gB** is slightly soluble in water.

The reaction of the tricyclic diketone **T** with TMP required higher temperature and longer time than the ketalization of **B** to reach high conversion. Reflux in toluene (2.2 equiv TMP, 5 mol % *p*-TsOH) required 48 h to afford diol **tT** in 88% yield. After 24 h, the conversion was estimated to 60% by ^1^H NMR spectroscopy. The lower reactivity was probably caused by the steric hindrance imposed by the tricyclic diketone. The reaction mixture turned dark, but we did not observe the formation of any significant amounts of byproducts. In contrast to the glycerol diol **gB**, the TMP-based diol **tT** was a crystalline compound, obtained after chromatographic purification in 88% yield. We also attempted to directly crystallize the product in a 1:1 (v:v) mixture of petroleum ether and acetone, but the isolated yield of **tT** remained quite low (ca 30%). However, in this case, an aqueous extractive workup (brine/EtOAc) was necessary to remove the unreacted TMP prior to crystallization.

Next, an NMR analysis of diol **tT** and **gB** was carried out. **tT** consisted of two diastereomers in roughly a 1:1 ratio. These isomers are *C*_2_ symmetric **tT**-*trans* and *C*_s_ symmetric **tT**-*cis*, as indicated in Scheme 1 (for NMR analysis, see Figures S9–S11). Similarly to the previously studied **tB**, they differ by the mutual orientation of the terminal ethyl and hydroxymethyl groups. These isomers were partially separable by flash chromatography but were used as a mixture in the present work for practical reasons.

**Figure 1:**
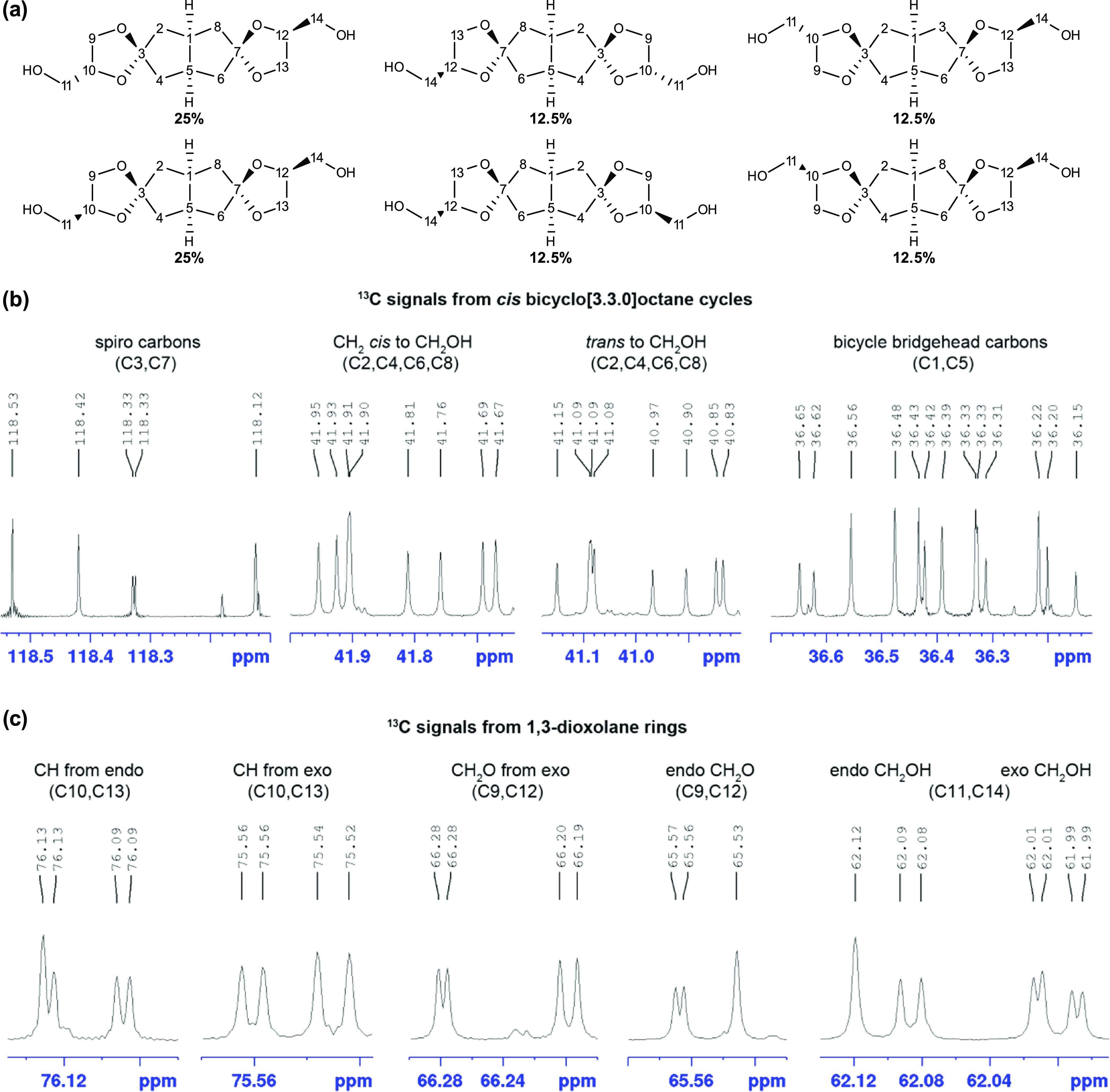
(a) Percentages of the relative amounts of specific isomer in the **gB** mixture. (b) ^13^C NMR spectrum showing cis bicyclo [3.3.0]octane cycle signals in DMSO-*d*_6_. (c) ^13^C NMR spectrum showing 1,3-dioxolane ring signals in DMSO-*d*_6_.

In contrast, diol **gB** consisted of numerous isomers. First, the glycerol can react with the ketone via a 1,2-addition, leading to a five-membered dioxalane-type ketal **gB** with hydroxymethyl group, or via 1,3-addition leading to dioxane-type ketals with secondary hydroxy groups (**x**, Scheme 1). In the first case (**gB**), the hydroxymethyl group can be connected to the *endo* or *exo* position of C-3 and C-7 of the *cis*-bicyclooctane ring and further isomers are obtained from the mutual different orientation of hydroxymethyl groups in the diketals (see structures in Figure 1). Although the ^1^H NMR spectrum at 800 MHz was noninformative about the composition of the mixture of compounds due to signal overlapping (Figure S1), the ^13^C NMR spectrum revealed the formation of a complex mixture of compounds (Figure 1). Inspection of the ^13^C NMR spectrum revealed that the most informative starting points were the regions of the spiro carbons (C3, C7) and the two carbon (C2/4 and C6/8) and oxygen atoms connected to C3/C7. There were two of them: around 119 ppm for the five-membered dioxolane ring, and around 109 ppm for the six-membered dioxane spiro compounds. The dioxolane region contained four pairs of signals with nearly equal intensities, representing about 90% of different isomeric ketals from the glycerol 1,2-ketalization. The minor ca. 10% components with dioxane ring consisted of six compounds, four of them having one dioxolane and one dioxane unit (*exo* and *endo cis* and *trans* isomers), and two isomers with only dioxane units (*cis* and *trans* isomers). These minor components were not further analyzed. The ^13^C spectrum of the main **gB** mixture revealed 58 ^13^C signals that were solvent-, concentration-, and temperature-dependent, of which 54 belonged to double-intensity signals and 4 to single-intensity signals. All 58 signals were distributed among 6 isomers, as shown in Figure S2, and they corresponded to the composition of 4 isomers with symmetrically and 2 isomers with unsymmetrically substituted spiro rings, resulting in about 2:2:1:1:1:1 ratios of the isomers with prevailing *endo–exo cis* and *trans C*_1_ symmetric isomers (Figure 1). The single-intensity signals in the bicyclo[3.3.0]octane bridgehead region belong to 2 *cis* isomers with *C*_s_ symmetry. Further details about the NMR analysis are presented in the Supporting Information.

We evaluated the hydrolytic stability of the **tT** and **gB** spirodiols by dissolution in 10 mM aqueous trifluoroacetic acid (TFA) in CD_3_CN at 20 °C. These conditions have been previously employed to study the stability of related acetals and ketals.^14,51^ The results showed that the hydrolysis rates of **tT** and **gB** were comparable under these conditions (Figures S34 and S35). After 8 h, 50% of **tT** and 56% of **gB** had been hydrolyzed, and after 24 h, 72 and 65% of the respective diols were degraded. After 1 week, both diols had almost completely been hydrolyzed into the respective original diketones and triols. The previously reported diol **tB** was found to degrade slightly faster under these conditions (∼95% after 24 h).^14^ Somewhat surprisingly, the glycerol diol **gB** was found to be very sensitive to trace amounts of HCl and water present in the undried and nonstabilized CDCl_3_, and signs of ketal cleavage were observed by ^1^H NMR analysis already after a few hours in this solvent. For that reason, all NMR analyses of **gB** were conducted in either DMSO-*d*_6_ or CD_3_CN. The TMP diols **tT** and **tB**, on the other hand, were more stable and could be analyzed in CDCl_3_.

**Scheme 2:**
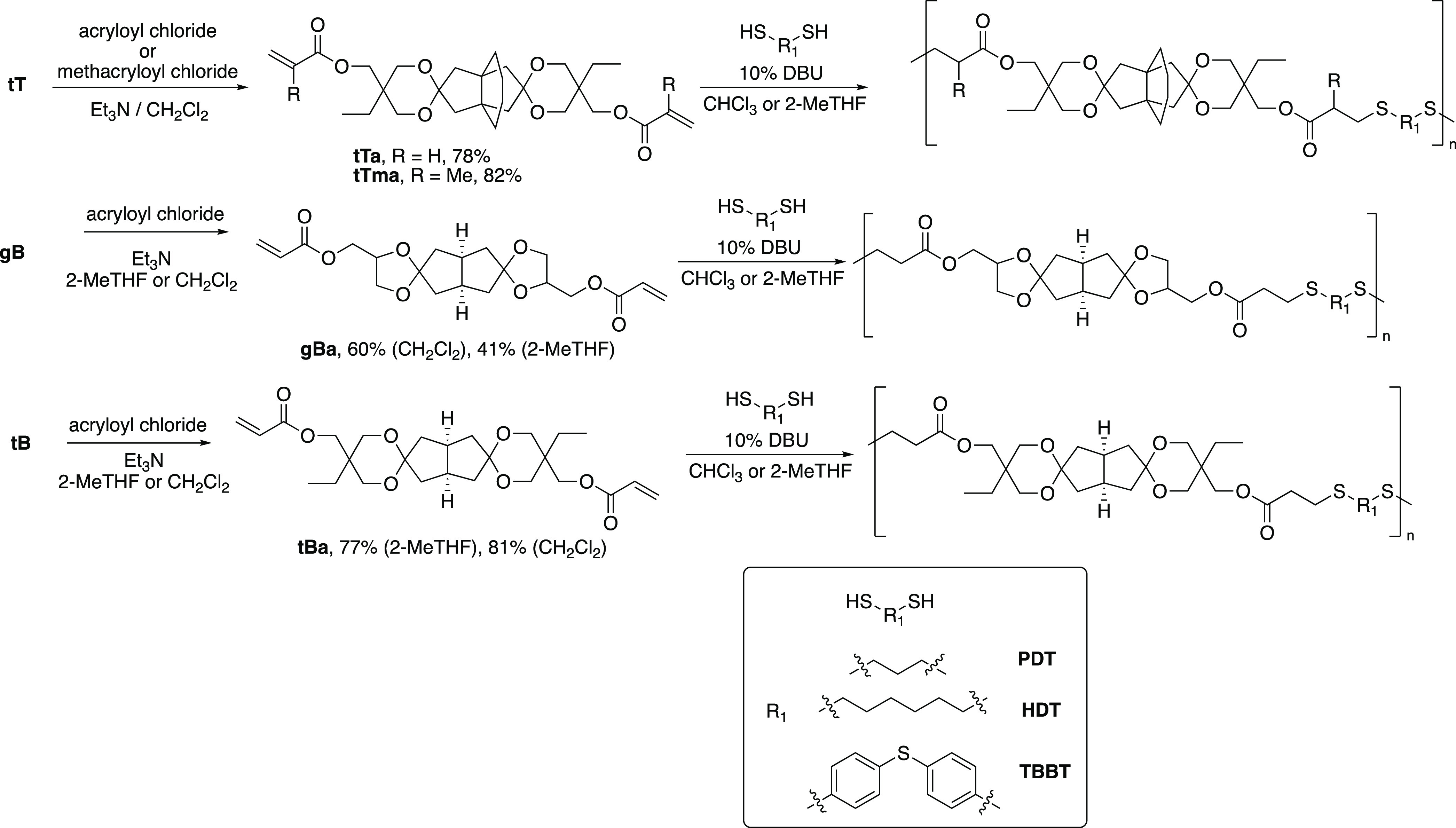
Preparation of Diacrylates and Dimethacrylates from the Different Spirodiols, Followed by Thiol-Michael Polymerizations with PDT, HDT, and TBBT (Mixtures of the Diol Isomers Were Used)

The diols **tT**, **gB**, and **tB** were then converted into the corresponding diacrylates, named as **tTa**, **gBa**, and **tBa** (“a” for acrylate), respectively, using acryloyl chloride/Et_3_N in CH_2_Cl_2_, or in the more environmentally friendly 2-MeTHF, with yields in the range 60–81 and 41–77%, respectively (Scheme 2). In the case of **gBa**, the small amounts of isomers with six-membered dioxane spiro rings were conveniently removed by flash chromatography and only dioxalane isomers were present in purified **gBa**. The diol **tT** was also converted into the dimethacrylate derivative **tTma** in 82% yield (“ma” for methacrylate).

### 3.2 Thiol-Michael Polymerizations

A range of thiol-Michael polymerizations with commercially available dithiols were carried out to study the influence of the spirocyclic arrangement, (meth)acrylate functionality, and dithiol structure (Tables 1–3). As dithiols, we selected the aliphatic 1,6-hexanedithol (HDT) and 1,3-propanedithiol (PDT), and the aromatic 4,4′-thiobisbenzenethiol (TBBT) to vary the chain stiffness. The resulting poly(β-thioether ester ketal)s were prepared by dissolving the di(meth)acrylate and the dithiol monomer in CHCl_3_ or 2-MeTHF. Next, the mixture was cooled to 0 °C using an ice bath, before adding 0.1 equiv of DBU and keeping the mixture under stirring for 24 h. Afterward, the mixture was precipitated in MeOH, filtered, and thoroughly dried. The polymers were named based on the corresponding monomers, given in parentheses, preceded by “poly” to indicate a polymer sample. For example, the polymer prepared from the bicyclic glycerol diacrylate **gBa** and 1,3-propanedithiol **PDT** was named “poly(gBa-PDT)”.

**Figure 2:**
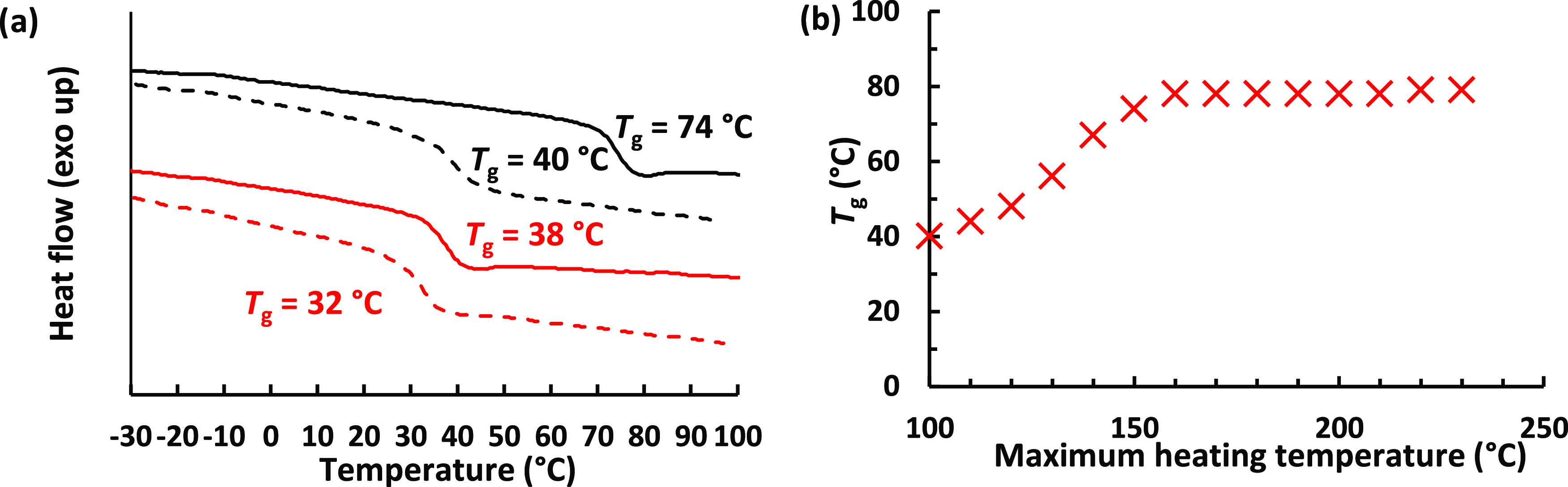
(a) DSC heating traces of poly(tTa-TBBT) (black) and poly(tTma-PDT) (red) recorded after annealing at 200 (solid lines) and 100 °C (dashed lines), respectively, showing the increase of *T*_g_ with the annealing temperature. (b) *T*_g_ measured by DSC analysis of poly(tTa-TBBT) after the sample had been heated to the given maximum temperature in the preceding heating scan.

The thermal transitions of the poly(β-thioether ester ketal)s were determined by differential scanning calorimetry (DSC). *T*_g_ marks the onset of coordinated segmental motions of the polymer chains at which the material softens and loses its mechanical rigidity. Hence, *T*_g_ typically dictates the upper use temperature for an application but is also important for the processing conditions. In our initial DSC measurements, we observed that the recorded *T*_g_ depended on the maximum temperature that the sample had been exposed to. When the samples were annealed at 200 °C, a higher *T*_g_ value was obtained compared to when the same sample was kept at 100 °C (Figure 2a). Additional investigations showed that a stepwise increase of the annealing temperature resulted in a gradual increase of *T*_g_ until a plateau value was reached (Figure 2b). This was likely caused by increasing cross-linking reactions occurring when the sample was kept at increasing temperatures. We speculate that the cross-linking may occur via thermally induced radical coupling reactions or polymerization of remaining (meth)acrylate end groups. The opening of ketal rings upon heating is a further plausible cross-linking mechanism. Indeed, samples became partially insoluble after annealing at 200 °C, thus confirming the cross-linking. Hence, all *T*_g_ values reported in the present study were determined by DSC analysis of samples that had not been heated above 100 °C, and which remained soluble.

To investigate the relationship between the structure of the poly(β-thioether ester ketal)s and the resulting physical properties, we studied the influence of three structural parameters, namely, the spirocyclic structure of the di(meth)acrylate monomer, the acrylate type (i.e., acrylate or methacrylate), and the structure of the dithiol monomer.

### 3.3 Influence of the Spirocyclic Structure

**Figure 3:**
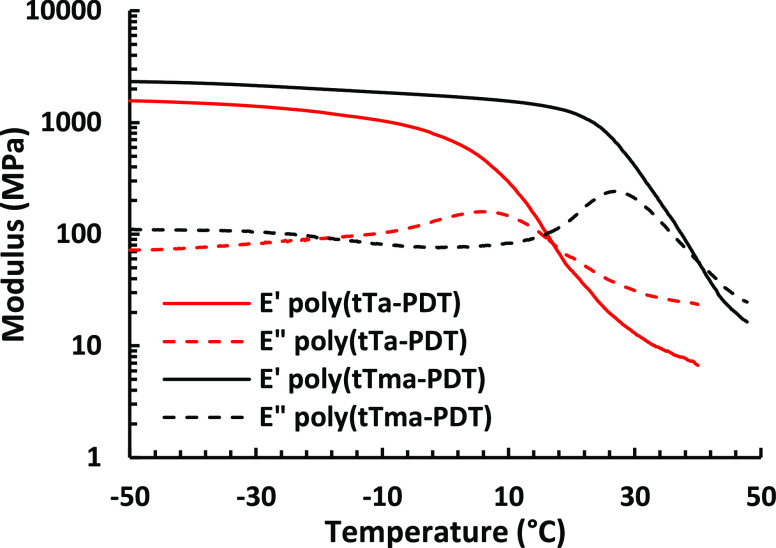
Storage (*E*′) and loss (*E*″) moduli of poly(tTa-PDT) and poly(tTma-PDT) measured by DMA at 1 Hz and 0.1% strain.

We selected the aliphatic PDT as the common dithiol monomer for the polymers prepared in this study (Table 1). The polymerizations with the different diacrylates afforded thiol–ene polymers with similar yields (70–77%). However, the molar masses varied quite significantly, from 15 to 26 kg/mol. Somewhat surprisingly, the polymerization with the glycerol-derived **gBa** was more sluggish compared to **tTa** and **tBa**, and a longer reaction time (40 h) was needed (for experimental details, see the Supporting Information). Interestingly, polymer poly(tTa-PDT), derived from the propellane-containing diacrylate **tTa**, had a much larger dispersity compared to the other poly(β-thioether ester ketal)s. We also evaluated the bio-based 2-MeTHF solvent in the polymerization of **tBa** and PDT (Table 1). Switching from CHCl_3_ to 2-MeTHF had a negligible effect on the polymer yield, but the molar mass dropped by a factor of two (entries 1 vs 2). This can be explained by the lower monomer solubility in the latter solvent. The thermal decomposition temperatures (*T*_d,95_) measured by TGA analysis were all very close to 320 °C. As expected, samples poly(gBa-PDT), poly(tBa-PDT), and poly(tTa-PDT) were all fully amorphous, and showed *T*_g_’s of −7, 15, and 24 °C, respectively (Table 1). As expected, *T*_g_ increased with the rigidity of the polymer backbone. The tricyclic structure of poly(tTa-PDT) is more rigid than the bicyclic one of poly(tBa-PDT), which provided the highest *T*_g_ to the former sample. Besides the cyclic structure, it appears that the six-membered ring, originating from the ketalization reaction with TMP, increased *T*_g_, compared to the five-membered ring formed by ketalization with glycerol. It is possible that the six-membered ring added to the rotational barrier in the polymer chain, thus reducing the segmental mobility of poly(tBa-PDT) compared to poly(gBa-PDT). However, this difference in *T*_g_ could also be the result of the larger molar mass of poly(tBa-PDT). It was not possible to analyze poly(tBa-PDT) and poly(gBa-PDT) by DMA because of the low *T*_g_’s and softness of the materials. DMA of poly(tTa-PDT) is shown in Figure 3.

**Table 1:** Polymerization and Thermal Data of Polymers with Different Spirocyclic Arrangements

entry	polymer	*M*_n_^a^ (kg/mol)	*M*_w_ (kg/mol)	*Đ*^a^	yield^b^ (%)	*T*_g_ by DSC^c^ (°C)	*T*_d,95%_^d^ (°C)	*T*_g_ by DMA^e^ (°C)
1	poly(tBa-PDT)	26	48	1.8	77	15	320	
2	poly(tBa-PDT)^f^	14	21	1.5	74	n.d.	n.d.	
3	poly(gBa-PDT)	15	29	1.9	70	–7	322	
4	poly(tTa-PDT)	18	64	3.6	78	24	323	17

a Measured by SEC in CHCl_3_.

b Isolated yield.

c *T*_g_ measured using DSC.

d Thermal degradation temperature at a 5% mass loss.

e *T*_g_ measured using DMA.

f Polymerization carried out in 2-MeTHF instead of CHCl_3_.

### 3.4 Influence of the (Meth)acrylate Functionality

We studied the effect and reactivity of the diacrylate vs dimethacrylate in polymerizations with PDT (Table 2). We further decided to use the monomers based on the tricyclic **tT** because of its rigid structure, which is likely to lead to high *T*_g_’s in relation to bicyclic monomers. The dimethacrylate-based polymer poly(tTma-PDT) was obtained in a slightly lower yield and molar mass, compared to the diacrylate equivalent. The somewhat lower reactivity of the methacrylate monomer can be explained by the electron-donating effect of the additional methyl group, which increases the electron density of the double bond, making it a less reactive Michael acceptor.^27^ The steric effect of the methyl group might also influence the methacrylate reactivity.

**Table 2:** Polymerization and Thermal Data of Polymers Prepared with Diacrylate and Dimethacrylate Functionality, Respectively

entry	polymer	*M*_n_^a^ (kg/mol)	*M*_w_ (kg/mol)	*Đ*^a^	yield^b^ (%)	*T*_g_ by DSC^c^ (°C)	*T*_d,95%_^d^ (°C)	*T*_g_ by DMA^e^ (°C)
1	poly(tTa-PDT)	18	64	3.6	78	24	323	17
2	poly(tTma-PDT)	14	37	2.6	68	32	315	27

a Measured by SEC in CHCl_3_.

b Isolated yield.

c *T*_g_ measured using DSC.

d Thermal degradation temperature at a 5% mass loss.

e *T*_g_ measured using DMA.

TGA data indicated a similar thermal stability for the two samples, and DSC analysis showed that both poly(β-thioether ester ketal)s were fully amorphous. The methacrylate-based polymer poly(tTma-PDT) had an 8 °C higher *T*_g_ than its acrylate counterpart, poly(tTa-PDT), with *T*_g_ = 32 °C. This trend is common when comparing acrylate and methacrylate-based polymers because the methyl group of the methacrylic unit increases the rotational barrier of the polymer backbone.^52^ The mechanical properties of poly(tTa-PDT) and poly(tTma-PDT) were investigated by dynamic mechanical analysis (DMA). Figure 3 shows the storage modulus (*E*′) and loss modulus (*E*″) of the samples at 1 Hz in the linear viscoelastic region (0.1% bending strain). As seen, the *E*′ value of the glassy plateau was higher for the methacrylate than the acrylate polymer. Thus, poly(tTma-PDT) showed an *E*′ value of 2.1 GPa at −30 °C, while poly(tTa-PDT) reached an *E*′ value of 1.4 GPa at the same temperature. The glass transition is marked by the decrease of *E*′, concurrently followed by an increase in *E*″. The glass-transition region seemed to be broader for poly(tTa-PDT), which can be explained by the higher value of *Đ*, indicating a more heterogeneous sample. Both samples deformed before reaching the rubbery plateau, which discontinued the measurements. The *T*_g_ values reported in Table 2 were determined as the maximum in *E*″, which gave *T*_g_ = 17 and 27 °C for poly(tTa-PDT) and poly(tTma-PDT), respectively. These values agreed well with the values obtained by DSC.

### 3.5 Influence of the Dithiol Structure

In addition to PDT, the more flexible HDT and stiffer aromatic dithiol TBBT were investigated in polymerizations with the propellane-containing diacrylate tTa to study the effect of the thiol component on the polymerizations and the properties. The yields of all of the isolated polymers were in the range of 70–77% (Table 3), indicating a similar reactivity of the different dithiols. As expected, DSC analysis showed that the polymers were completely amorphous and the *T*_g_ values were 24, 27, and 40 °C for poly(tTa-PDT), poly(tTa-HDT), and poly(tTa-TBBT), respectively. *T*_g_ was expected to increase with the rigidity of the polymer chain and should thus increase from the very flexible HDT-based polymer to the PDT-based polymer, and the sample based on the aromatic TBBT should reach the highest value. The discrepancy observed with poly(tTa-HDT) and poly(tTa-PDT) may be explained by the significantly lower *M*_n_ value of the latter sample.

**Table 3:** Polymerization and Thermal Data Comparison Using Different Dithiols

entry	polymer	*M*_n_^a^ (kg/mol)	*M*_w_ (kg/mol)	*Đ*^a^	yield^b^ (%)	*T*_g_ by DSC^c^ (°C)	*T*_d,95%_^d^	*T*_g_ by DMA^e^ (°C)
1	poly(tTa-HDT)	27	99	3.6	70	27	320	22
2	poly(tTa-PDT)	18	64	3.6	78	24	323	17
3	poly(tTa-TBBT)	22	39	1.8	77	40	317	35

a Measured by SEC in CHCl_3_.

b Isolated yield.

c *T*_g_ measured using DSC.

d Thermal degradation temperature at a 5% mass loss.

e *T*_g_ measured using DMA.

**Figure 4:**
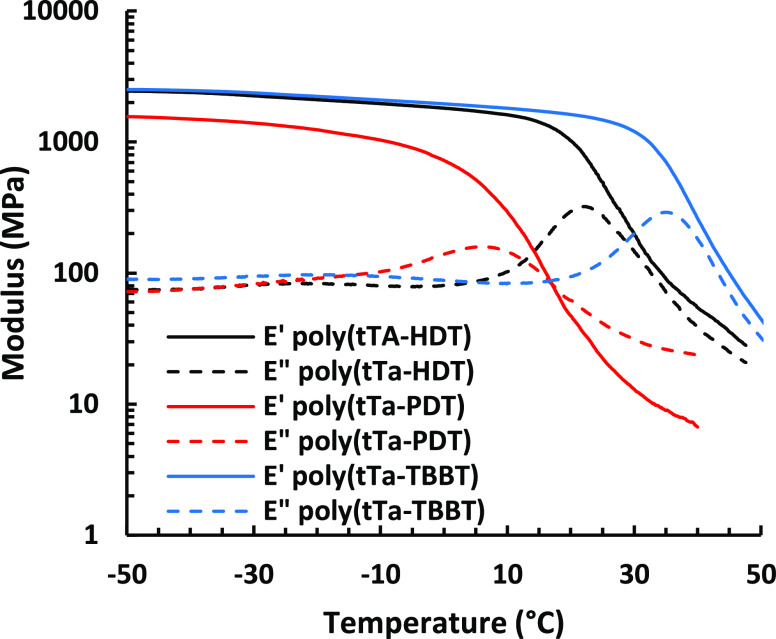
Storage (*E*′) and loss (*E*″) moduli of poly(tTa-HDT), poly(tTa-PDT), and poly(tTa-TBBT), measured by DMA at 1 Hz and 0.1% strain.

Figure 4 shows temperature sweeps of the storage (*E*′) and loss modulus (*E*″) obtained by DMA of the samples at 1 Hz and 0.1% bending strain. The *E*′ value on the glassy plateau was observed to be higher for poly(tTa-TBBT), which was consistent with the presence of the TBBT moiety that increases the rigidity of the polymer chain. Thus, poly(tTa-TBBT) had an *E*′ value of 2.4 GPa at −30 °C, while poly(tTa-HDT) reached an *E*′ value of 2.3 GPa at the same temperature. Poly(tTa-PDT) presented a much lower *E*′ value at −30 °C (1.4 GPa), which again may be explained by the significantly lower *M*_n_ value of this polymer. The *T*_g_ values reported in Table 3 were determined as the maximum in *E*″, which gave *T*_g_ = 22, 17, and 35 °C for poly(tTa-HDT), poly(tTa-PDT), and poly(tTa-TBBT), respectively. This was in agreement with the DSC data.

**Figure 5:**
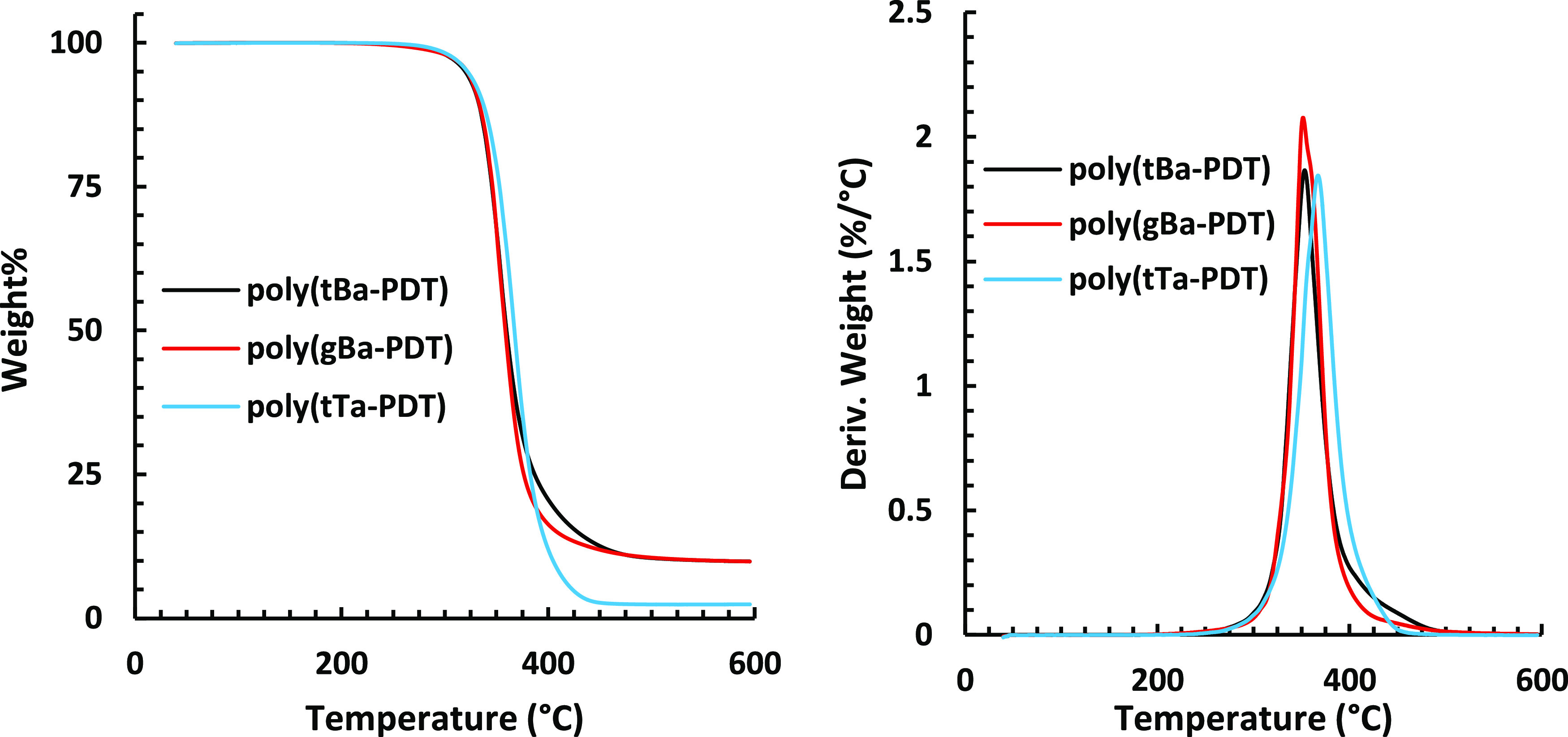
TGA traces (wt % and derivative) of poly(tBa-PDT), poly(gBa-PDT), and poly(tTa-PDT), under N_2_ atmosphere at 10 °C/min.

All of the studied poly(β-thioether ester ketal)s showed a single decomposition step in a narrow range from *T*_d,95%_ = 315–323 °C (Figures 5 and S33). These values were thus more than 200 °C above *T*_g_, indicating that the thermal window is sufficiently high to enable melt processing of these polymers ca. 30–70 °C above *T*_g_ without thermal decomposition. Still, as already discussed above, the polymers are likely to start cross-linking if heated above ca. 100 °C.

### 3.6 Hydrolytic Degradation and Chemical Recyclability of Polymers

The stability of the poly(β-thioether ester ketal)s was initially evaluated by keeping solid pieces of poly(tTma-PDT), poly(tTa-TBBT), poly(tBa-HDT), and poly(gBa-PDT) in aqueous solutions at pH = 0, 3, 8 and 14, respectively, for 14 days at 37 °C. After the immersion, the samples were dried, weighed, and analyzed by SEC in CHCl_3_. No notable changes in sample mass or molar mass were observed after this treatment.

Since the samples degraded very slowly in a purely aqueous environment, poly(tTma-PDT) was immersed in a 1:1 (v:v) mixture of acetic acid and water at 90 °C. TLC analysis of a sample taken 6 h after the immersion showed the formation of diketone **T**. Estimating the amount from NMR spectra was however complicated due to overlapping signals. Acetal bond degradation under these conditions has previously been reported for thiol–ene cross-linked networks containing acetal linkages after just a couple of hours.^42^ Next, the stability of the same polymer samples was evaluated by immersion in a water–acetone mixture [0.1 M aqueous HCl/acetone, 1:9 (v:v)] at 50 °C. The solid polymer pieces slowly dissolved after 5–8 h, and no solid sample pieces were left [except for poly(tTa-TBBT), which had several small pieces left after 8 h, most likely caused by the overall poor solubility of the polymer due to cross-linking]. After 72 h, the solvents were evaporated and the residues were dissolved in CDCl_3_ and THF for NMR and SEC analysis, respectively. The SEC analysis showed only polymer fragments with *M*_n_ values less than 1500 g/mol, which indicated a drastic degradation of the polymer chains. ^1^H NMR analysis showed the formation of a small amount of propellane diketone **T** (around 10 mol %). In the case of poly(gBa-PDT), the detected amount of the corresponding bicyclic diketone **B** was slightly higher, around 20 mol %. The estimated amounts of diketones after hydrolysis were calculated by comparing ^1^H NMR signal integrals of the TMP fragment methyl group (0.86 ppm) to the emerging diketone signals at 2.45 ppm. In the case of poly(gBa-PDT), the growing diketone signals at 2.22 ppm were compared to the CH_2_ signals from the PDT fragment at 2.68 ppm.

**Figure 6:**
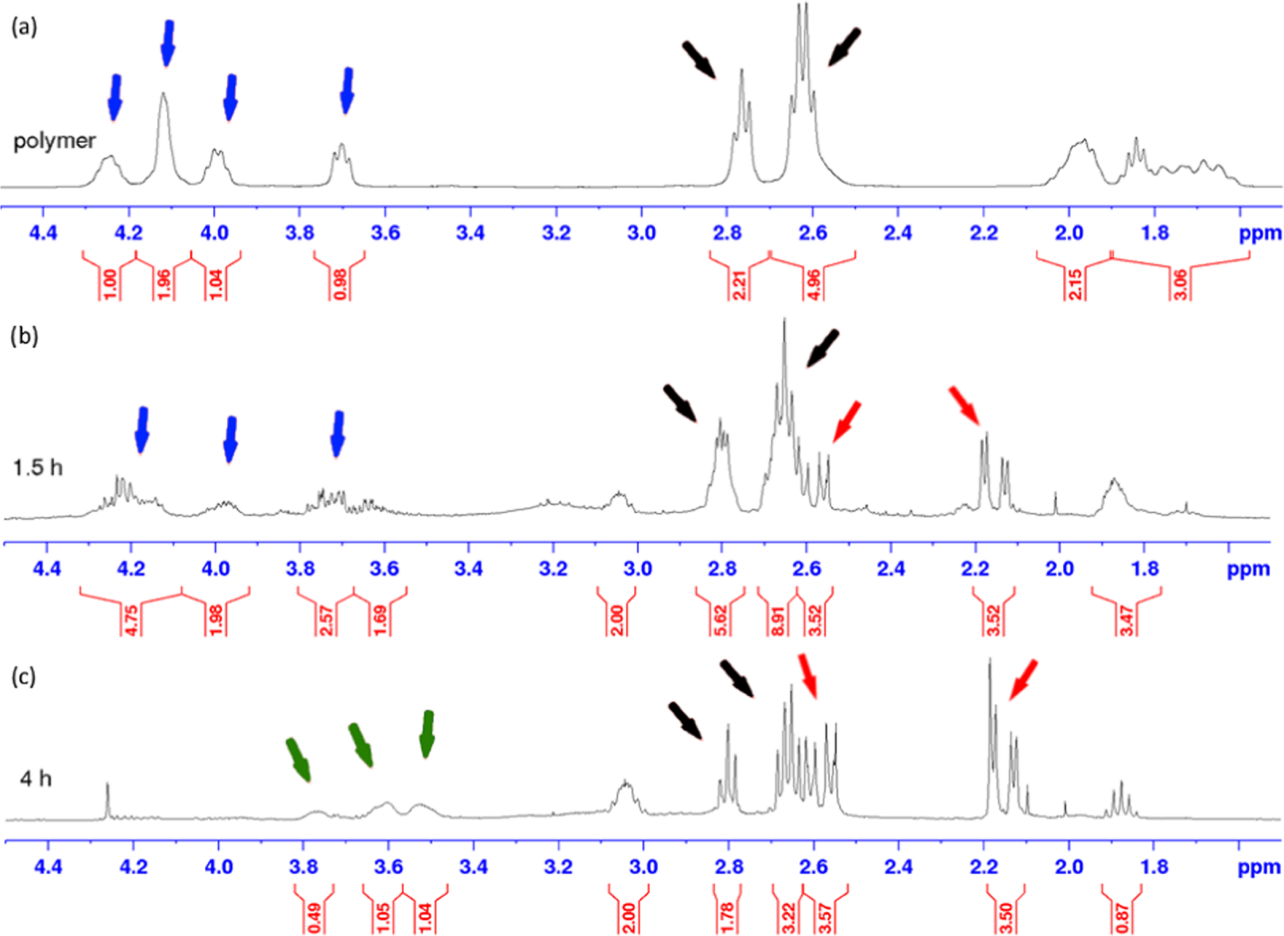
Poly(gBa-PDT) hydrolysis in 10% 1 M aqueous HCl in acetone monitored by ^1^H NMR analysis. Red arrows indicate the formation of diketone signals, and blue arrows indicate the disappearance of the signals from the dioxalane ring. Green arrows point at the appearance of glycerol signals, and black arrows show the signals from the PDT fragment.

When stronger acidic conditions were applied [1 M aqueous HCl/acetone, 1:9 (v:v)], the hydrolytic degradation occurred significantly faster. A film of poly(tTma-PDT) (60 mg) dissolved in a few hours after immersion at 50 °C. At that stage, the NMR analysis of the solution indicated the presence of ca. 20 mol % of diketone **T**. The amount of **T** gradually increased, and after 1 week, around 60 mol % of **T** was detected (Figure S37). The glycerol-derived sample poly(gBa-PDT) degraded significantly faster under the same conditions (Figure 6). The sample was visually completely dissolved after 1.5 h, and the ^1^H NMR spectrum of the liquid phase indicated the formation of the diketone with a parallel decrease of the dioxalane ring signals (Figure 6b). After 4 h, the ketal groups were fully hydrolyzed (Figure 6c) and essentially complete polymer chain degradation had occurred. Mixtures of water and organic solvents have previously been reported to degrade acetal-containing polymers. For example, acetal functionalities in polyurethane thermosets have been hydrolyzed under similar conditions.^53^

The degradation experiments indicated that the poly(β-thioether ester ketal)s were stable under both acidic and basic aqueous conditions. However, polymer degradation occurs via ketal hydrolysis in an acidic water–acetone mixture, where acetone most probably facilitates swelling, which significantly enhances the rate of hydrolysis. This path clearly opens up prospects for chemical recycling of these materials.

## 4 Conclusions

A straightforward synthetic pathway to two conformationally rigid biobased alicyclic spirodiols from readily available starting materials has been developed. Inexpensive citric acid was first converted into bicyclic and tricyclic ketones, which after subsequent ketalization with glycerol and trimethylolpropane, respectively, afforded rigid diol building blocks containing either five- or six-membered spirocyclic rings. The isomeric compositions of these novel diols were fully analyzed and assigned by advanced NMR spectroscopy methods.

Next, the new spirodiols were converted into corresponding di(meth)acrylates and evaluated in thiol–ene-type polymerizations with various dithiols. The thiol–ene polymerizations in the presence of a catalytic amount of DBU afforded poly(β-thioether ester ketal)s with thermal stability above 300 °C and *T*_g_ values ranging from −7 to 40 °C. The highest *T*_g_ was obtained by combining a tricyclic spiro diacrylate with an aromatic dithiol. An increase in *T*_*g*_ was seen upon successive heating of the samples, which may be the result of thermally induced cross-linking via residual acrylate end groups in the polymer. The polymers were stable in acidic and basic aqueous conditions (pH 0–14), but in a mixture of 1 M aqueous HCl/acetone (1:9), the ketal functionalities were cleanly hydrolyzed to afford the initial diketones, thus opening a path for chemical recycling of these materials. Overall, these results indicate that the novel spirodiols developed in this work are a valuable addition to the important list of rigid biobased monomers possible to use for the preparation of high-performance polymers.

## Supporting Information Available

Detailed monomer and polymer synthesis procdedures; NMR spectra of monomers and polymers; SEC graphs, DSC, and TGA curves of polymers; NMR spectra of monomer and polymer degradation studies; and polymer solubility data (PDF)
