# Supplementary material for: Chemically Recyclable Poly(β-thioether ester)s Based on Rigid Spirocyclic Ketal Diols Derived from Citric Acid
Source: Biomacromolecules. 2022 May 26;23(6):2685–96. doi: 10.1021/acs.biomac.2c00452 (PMC9198987; doi:10.1021/acs.biomac.2c00452)
Supplement: Supplementary file 1 — bm2c00452_si_001.pdf [file bm2c00452_si_001.pdf]

Supporting Information

CHEMICALLY RECYCLABLE POLY(Β-THIOESTER)S BASED ON RIGID SPIROCYCLIC KETAL DIOLS DERIVED FROM CITRIC ACID

Rauno Sedrik,[†] Olivier Bonjour,[§] Siim Laanesoo,[†] Ilme Liblikas,[†] Tõnis Pehk,[‡] Patric Jannasch*[,†,§], Lauri Vares*[,†]

[†] Institute of Technology, University of Tartu, Nooruse 1, Tartu 50411, Estonia
[‡] Laboratory of Chemical Physics, National Institute of Chemical Physics and Biophysics, Akadeemia tee 23, Tallinn 12618, Estonia
[§] Department of Chemistry, Lund University, Box 124, Lund 221 00, Sweden

Table of Contents

Monomer synthesis 3
Synthesis of [4.3.3]propellane-8,11-diketone T 3
Synthesis of propellane-spirodiol tB....................3
Synthesis of propellane-spiro-diacrylate tTa 4
Synthesis of propellane-spiro-dimethacrylate tTma 5
Synthesis of spiro-diacrylate tBa 5
Synthesis of glycerol-spiro-diacrylate gBa 6
Polymerizations....................7
Polymerization of poly(tTa-HDT) 7
Polymerization of poly(tTa-PDT)....................8
Polymerization of poly(tTa-TBBT) 8
Polymerization of poly(tTma-PDT)....................9
Polymerization of poly(tBa-PDT)....................9
Polymerization of poly(gBa-PDT)....................10
NMR analysis....................10
Glycerol spirodiol gB and diacrylate gBa NMR analysis....................10
[4.3.3]propellane spirodiol tT NMR analysis 16
NMR Spectra 18
Monomer ^{1}H and ^{13}C NMR spectra....................18
Polymer ^{1}H NMR spectra 28
Polymer characterization 34
SEC curves....................34
DSC traces 35
TGA traces....................36
Spirodiol hydrolytic stability studies 36

Polymer hydrolysis experiments....................37
Solubility of polymers....................38
References....................39

Monomer synthesis

Synthesis of [4.3.3]propellane-8,11-diketone T

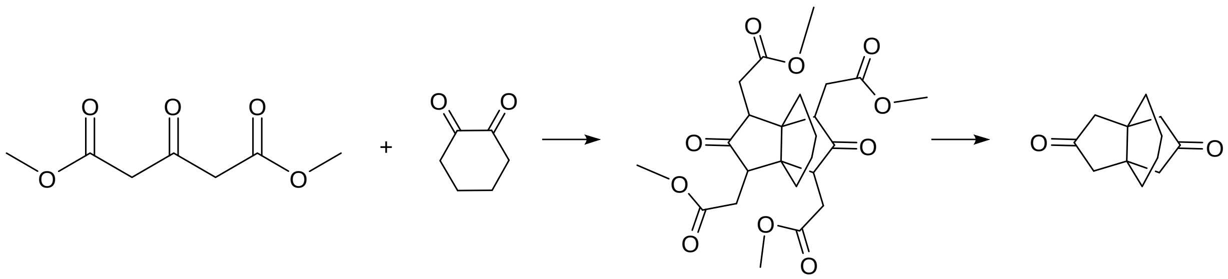

1 dimethyl-1,3-acetone dicarboxylate

2 cyclohexane-1,2-dione

3 8,11-dioxo[4.3.3]propellane-7,9,10,12-tetracarboxylate

T [4.3.3]propellane-8,11-dione

Cyclohexane-1,2-dione (**2**) (8.5 g, 75 mmol) was dissolved in 45 mL of methanol and 250 mL phosphate-citrate buffer (pH 5.6) and dimethyl-1,3-acetonedicarboxylate (**1**) (29.0 g, 166 mmol, 2.2 eq) were added. The mixture was stirred at room temperature overnight, after which a white precipitate started to form. The mixture was stirred for an additional 48 hours, the buffer solution was decanted, and the precipitate was washed three times with 50 mL of brine. After drying 25.7 g of the crude intermediate compound was obtained as a white solid (yield 80%). The product **3** was used without further purification in the next step.
Compound **3** was dissolved in hot methanol (55 mL), 200 mL of 6 M hydrochloric acid was added, and the mixture was refluxed overnight. The mixture was cooled down, extracted with CH_2Cl_2, and neutralized with a saturated aq. $NaHCO_3$ solution. The organic layer was dried over $MgSO_4$ and concentrated under reduced pressure to obtain the solid crude product, which was further purified by recrystallization from methanol (three times) to obtain 6.23 g (yield 43%) of white crystalline **T**.

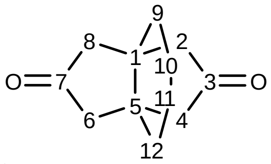

^{1}H NMR (400 MHz, $CDCl_3$): δ 2.45 (d, J=19.2 Hz, $CH_{2(2,4,6,8\text{-exo})}$,4H), 2.27 (d, J=19.4 Hz, $CH_{2(2,4,6,8\text{-endo})}$, 4H), 1.54 ($CH_{2(9,10,11,12)}$, 8H). (**Fig. S12**)
^{13}C NMR (100 MHz, $CDCl_3$): δ 216.8 ($CO_{(3,7)}$), 48.9 ($C_{(1,5)}$), 44.5 ($CH_{2(2,4,6,8)}$), 31.5 ($CH_{2(9,12)}$), 20.9 ($CH_{2(10,11)}$). (**Fig. S13**)
HRMS (ESI): calculated for $C_{12}H_{16}O_2$ $[M + Na]^+$ 215.1048, found 215.1042.
HRMS (ESI): calculated for $C_{12}H_{16}O_2$ $[M + H]^+$ 193.1228, found 193.1223.
R_f = 0.23 (20% EtOAc in petrol ether)

Synthesis of propellane-spirodiol tB

B (2.30 g, 16.65 mmol), TMP (5.36 g, 39.95 mmol, 2.40 eq) and *p*-toluenesulfonic acid monohydrate (92 mg, 0.48 mmol, 0.029 eq) were weighted into the flask. After that, toluene (80 mL) was added. The reaction flask was equipped with Dean-Stark apparatus and the mixture was refluxed overnight. After cooling down, the crude reaction mixture was concentrated under the reduced pressure. The crude product was purified by flash chromatography (5% MeOH/CH_2Cl_2) to obtain **tB** as a colorless solid compound (5.77 g, 93.5%).

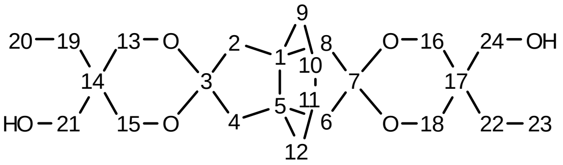

^{1}H NMR (400 MHz, $CDCl_3$): δ 3.77 (bs, $CH_{2(14,20)}$, 2H), 3.74 (bs, $CH_{2(14,20)}$, 2H), δ: 3.69 (dd, $^{2}J_{HH}$=11.8 Hz, $^{4}J_{HH}$=2.0 Hz, $CH_{2(9e,11e,15e,17e)}$, 4H), 3.55 (dd, $^{2}J_{HH}$=11.6 Hz, $^{3}J_{HH}$=7.7 Hz, $CH_{2(9a,11a,15a,17a)}$, 4H), 2.53 (m, $CH_{(1,5)}$, 2H), 2.29 (m, $CH_{2(2,4,6,8)}$, 2H), 2.08 (m, $CH_{2(2,4,6,8)}$, 2H), 1.71 (m, $CH_{2(2,4,6,8}$, 4H), 1.29 (q, $^{4}J_{HH}$=7.7 Hz, $CH_{2(12,18)}$, 2H), 1.25 (q, $^{4}J_{HH}$=7.7 Hz, $CH_{2(12,18)}$, 2H), 0.83 (t, $^{4}J_{HH}$=7.7 Hz, $CH_{3(13,19)}$, 3H), 0.82 (t, $^{4}J_{HH}$=7.7 Hz $CH_{3(13,19)}$, 3H)
^{13}C NMR (100.6 MHz, $CDCl_3$): δ 110.3 ($C_{(3,7)}$), 67.1 ($CH_{2(14,20)}$) 66.3 ($CH_{2(9,15)}$), 63.0 ($CH_{2(11,17)}$), 43.4 ($CH_{2(2,4,6,8)}$), 42.9 ($CH_{2(2,4,6,8)}$), 38.1 ($CH_{(1,5)}$), 36.9 ($CH_{(1,5)}$), 36.9 ($C_{(10,16)}$), 36.8 ($C_{(10,16)}$), 36.7 ($CH_{2(2,4,6,8)}$), 36.1 ($CH_{2(2,4,6,8)}$), 35.8 ($CH_{(1,5)}$), 23.8 ($CH_{2(12,\ 18)}$), 7.0 ($CH_{3(13,19)}$).
R_f = 0.22 (5% MeOH in CH_2Cl_2)

Table S1. Optimization of the ketalization reaction between diketone **B** and glycerol to afford spiro-diol **gB**.

Entry	Glycerol (equiv.)	Catalyst (mol%)	Solvent	Reflux time (h)	Oil bath temp (°C)	Isolated yield of **gB** (%)	Comment
1	3.6	4	Toluene	5	140	54	Incomplete conversion, turned very dark, ca 10% monoketone also isolated
2	2.4	1	Toluene	16	140	17	A lot of black by-products
3	4	10	cHex	4	105	64	Incomplete conversion, no dark byproducts
4	2.6	5	cHex	18	105	50	Incomplete conversion, some dark byproducts
5	6	5	cHex:DMF (5:1)	23	115	66	Full conversion (no diketone or monoketone left), lower yield due to dark byproducts
6	6	5	cHex:DMF (5:1)	4	115	44	Incomplete conversion, no dark byproducts
7	2.6	5	cHex:DMF (10:1)	7	115	84	No diketone left, small amount of monoketone left
**8**	**2.8**	**1**	**cHex:DMF (12.5:1)**	**15**	**115**	**92**	***Reported in the paper***
**9**	**2.4**	**2.3**	**cHex:Tol (2:1)**	**16**	**120**	**82**	***Reported in the paper*** *(traces of monoketone left in crude)*

Synthesis of propellane-spiro-diacrylate tTa

tT (4.023 g, 9.47 mmol) was dissolved in 40 mL of CH_2Cl_2, the flask was flushed with argon and capped with a rubber septum. The mixture was cooled on an ice bath and acryloyl chloride (1.715 g, 18.9 mmol) and Et_3N (4.0 mL, 27.2 mmol) were added slowly at the same time. The ice bath was removed, and the mixture was stirred overnight. The resulting mixture was then extracted once with aq. $NaHCO_3$. The organic layer was dried on $MgSO_4$ and concentrated under reduced pressure. The concentrate was purified by flash chromatography (10% EtOAc in petrol ether) to afford **tTa** as a transparent viscous liquid (3.961 g, yield 78%).

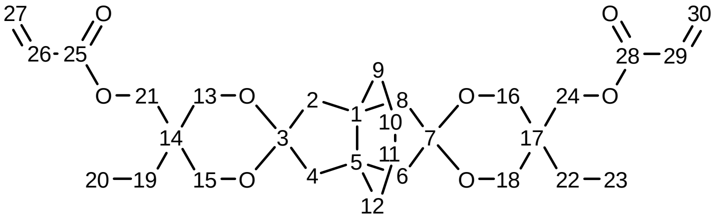

^{1}H NMR (400 MHz, $CDCl_3$): δ 6.39 (dd, $^3J_{HH}$=17.2 Hz, $^2J_{HH}$=1.4 Hz, $CH_{2E(27,30)}$, 2H), 6.11 (dd, $^3J_{HH}$=17.3 Hz, $^2J_{HH}$=10.3 Hz, $CH_{(26,29)}$, 2H), 5.82 (d, $^2J_{HH}$=10.5 Hz, $CH_{2Z(27,30)}$, 2H), 4.32 (s, $CH_{2(21,24)}$, 2H), 4.28 (s, $CH_{2(21,24)}$, 2H), 3.71-3.57 (m, $CH_{2(13,15,16,18)}$, 8H), 2.04 (m, $CH_{2(2,4,6,8)}$, 8H), 1.42-1.40 (m, $CH_{2(9,10,11,12)}$, 8H), 1.25 (q, $CH_{2(19,22)}$, 4H), 0.82 (t, $^3J_{HH}$=7.6 Hz, $CH_{3(20,23)}$, 3H), 0.81 (t, $^3J_{HH}$=7.6 Hz, $CH_{3(20,23)}$, 3H).
^{13}C NMR (100.6 MHz, $CDCl_3$): δ 167.0 ($CO_{(25,28)}$), 130.8 ($CH_{2(27,30)}$), 128.2 ($CH_{(26,29)}$), 109.0 ($C_{(3,7)}$), 66.6 ($CH_{2(13,15,16,18)}$), 66.4 ($CH_{2(13,15,16,18)}$) 65.9 ($CH_{2(13,15,16,18)}$), 64.3 ($CH_{2(13,15,16,18)}$), 63.8 ($CH_{2(13,15,16,18)}$), 60.4 ($CH_{2(21,24)}$), 49.4 ($CH_{2(2,4,6,8)}$), 49.2 ($C_{(1,5)}$), 49.1 ($CH_{2(2,4,6,8)}$), 47.0 ($C_{(1,5)}$), 45.7 ($C_{(1,5)}$), 44.5 ($CH_{2(2,4,6,8)}$), 44.0 ($CH_{2(2,4,6,8)}$), 43.0 ($CH_{2(2,4,6,8)}$), 35.7 ($C_{(14,17)}$), 31.5 ($CH_{2(9,12)}$), 31.3 ($CH_{2(9,12)}$), 23.8 ($CH_{2(19,22)}$), 21.2 ($CH_{2(10,11)}$), 7.3 ($CH_{3(20,23)}$), 6.9 ($CH_{3(20,23)}$).
HRMS (ESI): calculated for $C_{30}H_{44}O_8$ $[M + H]^+$ 533.3114, found 533.3109.
R_f = 0.66 (20% EtOAc in petrol ether).

Synthesis of propellane-spiro-dimethacrylate tTma

tT (4.058 g, 9.56 mol) was dissolved in 40 mL of CH_2Cl_2, the flask was flushed with argon and capped with a rubber septum. The mixture was cooled on an ice bath and methacryloyl chloride (2.498 g, 23.9 mmol, 2.5 eq) and Et_3N (4.0 mL, 27.2 mmol) were added slowly at the same time. The ice bath was removed, and the mixture was stirred overnight at room temperature. The resulting mixture was extracted with saturated aq. $NaHCO_3$. The organic layer was dried on $MgSO_4$ and concentrated under reduced pressure. The crude product was purified by flash chromatography (10% EtOAc in petrol ether) to obtain pure **tTma** as a transparent viscous liquid (4.40 g, yield 82%).

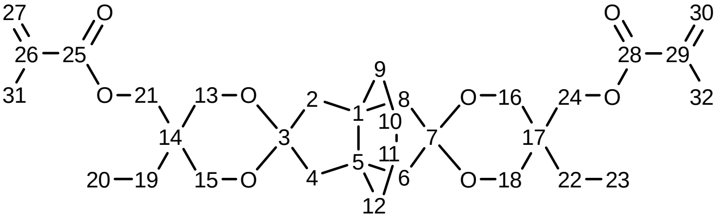

^{1}H NMR (400 MHz, CDCl3): δ 6.07 (m, $CH_{2(27,30)}$, 2H), 5.54 (m, $CH_{2(27,30)}$, 2H), 4.28 (s, $CH_{2(21,24)}$, 2H), 4.24 (s, $CH_{2(21,24)}$ 2H) 3.69-3.54 (m, $CH_{2(13,15,16,18)}$, 8H), 2.03 ($CH_{2(2,4,6,8)+EtOAc)}$, 8H), 1.93 ($CH_{3(31,32)}$, 6H), 1.38 (m,$CH_{2(9,10,11,12)}$, 8H), 1.23 (m, $CH_{2(19,22)}$, 4H), 0.81 (t, $^3J_{HH}$=7.6 Hz, $CH_{3(20,23)}$, 3H), 0.80 (t, $^3J_{HH}$=7.6 Hz, $CH_{3(20,23)}$, 3H).
^{13}C NMR (100.6 MHz, $CDCl_3$): δ 167.1 ($CO_{(25,28)}$), 136.2 ($CH_{2(27,30)}$), 125.3 ($CH_{(26,29)}$), 109.4 ($C_{(3,7)}$), 66.4 ($CH_{2(13,15,16,18)}$), 64.1 ($CH_{2(13,15,16,18)}$), 60.2 ($CH_{2(21,24)}$), 49.3 ($C_{(2,4,6,8)}$), 49.1 ($C_{(1,5)}$), 48.6 ($CH_{2(2,4,6,8)}$), 48.0 ($CH_{2(1,5)}$), 47.0 ($CH_{2(1,5)}$), 44.3 ($CH_{2(2,4,6,8)}$), 43.7 ($CH_{2(2,4,6,8)}$), 35.6 ($C_{(14,17)}$), 30.9 ($CH_{2(9,12)}$), 23.9 ($CH_{2(19,22)}$), 21.2 ($CH_{2(10,11)}$), 18.2 ($CH_{3(31,32)}$), 6.9 ($CH_{3(20,23)}$), 6.8 ($CH_{3(20,23)}$).
HRMS (ESI): calculated for $C_{32}H_{48}O_8$ $[M + H]^+$ 561.3427, found 561.3422.
R_f = 0.55 (20% EtOAc in petrol ether).

Synthesis of spiro-diacrylate tBa

tB (1.979 g, 5.34 mmol) in a round bottom flask was dissolved in dry CH_2Cl_2 (15 mL). The flask was flushed with argon, capped with a rubber septum, and cooled down on an ice bath.

Thereafter Et_3N (2.0 mL) and acryloyl chloride (0.99 mL, 2.2 eq) were added simultaneously dropwise. The ice bath was removed, and the mixture was stirred overnight. The completion of the reaction was estimated by TLC. The reaction was quenched by addition of saturated aq. $NaHCO_3$ (50 mL) and extracted three times with CH_2Cl_2 (3x 50 mL). The organic phases were combined, dried over $MgSO_4$, and concentrated under the reduced pressure. The product was purified via flash chromatography over silica gel (30% EtOAc in petroleum ether). The pure product was obtained as an oily viscous liquid (2.07 g, yield 81%).

Alternative method using 2-MeTHF as solvent.

tB (826 mg, 2.23 mmol) in a round bottom flask was dissolved in dry 2-MeTHF (30 mL). The flask was flushed with argon, capped with a rubber septum, and cooled down on an ice bath. Thereafter Et_3N (0.81 mL) and acryloyl chloride (0.42 mL, 5.22 mmol, 2.25 eq) were added simultaneously dropwise. The ice bath was removed, and the mixture was stirred for 48 hours at room temperature. The completion of the reaction was estimated by TLC. The reaction was quenched by addition of saturated aq. $NaHCO_3$ (50 mL) and extracted three times with EtOAc (3x 50 mL). The organic phases were combined, dried over $MgSO_4$, and concentrated under the reduced pressure. The product was purified by flash chromatography over silica gel (30% EtOAc in petroleum ether). The pure product was obtained as an oily viscous liquid (825 mg, yield 77%)

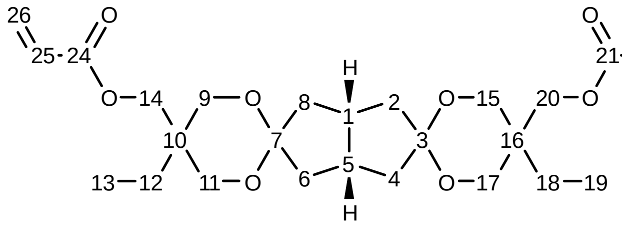

^{1}H NMR (400 MHz, $CDCl_3$): *d* 6.36 (dd, $^3J_{HH}$= 17.4 Hz, $^2J_{HH}$=1.2 Hz, $CH_{2(23E,26E)}$, 2H), 6.09 (dd, $^3J_{HH}$= 17.5 Hz, $^3J_{HH}$=10.4 Hz, $CH_{(22,25)}$, 2H), 5.76 (dd, $^3J_{HH}$= 10.4 Hz, $^2J_{HH}$=1.2 Hz, $CH_{2(23Z,26Z)}$, 2H), 4.29 (bs, $CH_{2(14,20)}$, 2H), 4.24 (bs, $CH_{2(14,20)}$, 2H), 3.68 (dd, $^2J_{HH}$=11.8 Hz, $^4J_{HH}$=1.3 Hz, $CH_{2(9a,11a,15a,17a)}$, 4H), 3.55 (dd, $^2J_{HH}$=11.8 Hz, $^3J_{HH}$=6.0 Hz, $CH_{2(9a,11a,15a,17a)}$, 4H), 2.51 (m, $CH_{(1,5)}$, 2H), 2.23 (m, $CH_{2(2,4,6,8)}$, 2H), 2.06 (m, $CH_{2(2,4,6,8)}$, 2H), 1.70 (m, $CH_{2(2,4,6,8)}$, 4H), 1.32 (t, $^3J_{HH}$=7.6 Hz, $CH_{2(12,18)}$, 2H), 1.27 (t, $^3J_{HH}$=7.6 Hz, $CH_{2(12,18)}$, 2H), 0.79 (q, $^3J_{HH}$=7.6 Hz, $CH_{3(13,19)}$, 3H), 0.78 (q, $^3J_{HH}$=7.6 Hz, $CH_{3(13,19)}$, 3H)

^{13}C NMR (100.6 MHz, $CDCl_3$): *d* 166.0 ($CO_{(21,14)}$), 130.6 ($CH_{2(23,26)}$), 128.2 ($CH_{(22,25)}$), 110.2 ($C_{(3,7)}$), 66.8 ($CH_{2(14,20)}$), 66.1 ($CH_{2(9,15)}$), 63.8 ($CH_{2(11,17)}$), 43.0 ($CH_{2(2,4,6,8)}$), 42.2 ($CH_{2(2,4,6,8)}$), 37.8 ($CH_{1,5}$), 37.0 ($CH_{2(2,4,6,8)}$), 36.8 ($C_{(1,5)}$), 36.2 ($CH_{2(2,4,6,8}$), 35.9 ($CH_{(1,5)}$), 35.8 ($C_{(10,16)}$), 23.8 ($CH_{2(12,18)}$), 6.8 ($CH_{3(13,19)}$).

HRMS (ESI): calculated for $C_{26}H_{39}O_8$ $[M + H]^+$ 479.2639, found 479.2635.

R_f = 0.52 (25% EtOAc in petrol ether).

Synthesis of glycerol-spiro-diacrylate gBa

gB (3.915 g, 13.67 mmol) was dissolved in dry CH_2Cl_2 (30 mL). The flask was capped with a rubber septum, flushed with argon, and cooled down using an ice bath. Acryloyl chloride (3.27 mL, 34.18 mmol, 2.4 eq) and Et_3N (4.76 mL) were added simultaneously dropwise. The ice bath was removed, and the mixture was stirred overnight at room temperature. The reaction was quenched by addition of saturated aq. $NaHCO_3$ (50 mL) and extracted three times with CH_2Cl_2 (3x 50 mL). The organic phases were combined, dried over $MgSO_4$, and concentrated under reduced pressure. The mixture was purified by silica flash column (30% EtOAc in petrol ether). The pure product was obtained as an oily viscous liquid (3.219 g, yield 60%).

Alternative method using 2-MeTHF as solvent.
gB (0.938 g, 3.28 mmol) was dissolved in dry 2-MeTHF (32 mL). The flask was capped with a rubber septum, flushed with argon, and cooled down using an ice bath. Acryloyl chloride (0.71 mL, 7.51 mmol, 2.2 eq) and Et_3N (1.2 mL) were added simultaneously dropwise. The ice bath was removed, and the mixture was stirred overnight at room temperature. The reaction was quenched by addition of saturated aq. $NaHCO_3$ (50 mL) and extracted three times with EtOAc (3x 50 mL). The organic phases were combined, dried over $MgSO_4$, and concentrated under reduced pressure. The mixture was purified by silica flash column (35% EtOAc in petrol ether). The pure product was obtained as an oily viscous liquid (0.534 g, yield 41%).

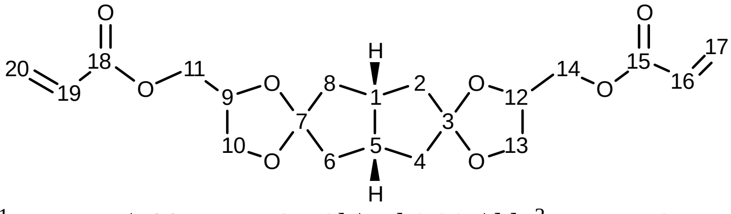

^{1}H NMR (400 MHz, $CDCl_3$): *d* 6.39 (dd, $^{3}J_{HH}$= 17.2 Hz, $^{2}J_{HH}$=1.3 Hz, $CH_{2(17E, 20E)}$, 2H), 6.10 (dd, $^{3}J_{HH}$= 17.2 Hz, $^{3}J_{HH}$=10.4 Hz, $CH_{(16,19)}$, 2H), 5.82 (dd, $^{3}J_{HH}$= 10.4 Hz, $^{2}J_{HH}$=1.3 Hz, $CH_{2(17Z,20Z)}$, 2H), 3.36-4.20 ($CH_{X(9,10,11,12,13,14)}$,10H), 2.56 ($CH_{(1,5)}$, 2H), 1.94 ($CH_{2(2,4,6,8)}$, 4H), 1.68 ($CH_{2(2,4,6,8)}$, 4H).
^{13}C NMR (100.6 MHz, $CDCl_3$): *d* 165.7 ($CO_{(11,15)}$), 131.31 ($CH_{2(17,20)}$), 127.8 ($CH_{(16,19)}$), 119.6 ($C_{(3,7)}$), 73.4 ($CH_{(9,12)}$), 72.8 ($CH_{(9,12)}$), 66.5 ($CH_{2(10,11,13,14)}$), 66.4 ($CH_{2(10,11,13,14)}$), 65.8 ($CH_{2(10,11,13,14)}$), 66.7 ($CH_{2(10,11,13,14)}$), 64.5 ($CH_{2(10,11,13,14)}$), 42.2-41.1 ($CH_{2(2,4,6,8)}$), 37.0-36.6 ($CH_{(1,5)}$).
HRMS (ESI): calculated for $C_{20}H_{26}O_8$ $[M + Na]^+$ 417.1513, found 417.1520.
R_f = 0.34 (25% EtOAc in petrol ether).

Polymerizations

The di(meth)acrylate (typically about 500 mg) was dissolved in $CHCl_3$ (ca 100 mg/mL) and 1 equivalent of dithiol was added. The mixture was cooled on an ice bath and 0.1 equivalents of DBU was added as a solution in chloroform. The ice bath was removed shortly afterwards, and the mixture was stirred at room temperature for 24 hours. The polymer was then precipitated in 100 mL of MeOH and allowed to stir slowly overnight (16 h), after which the polymer had precipitated to the bottom. The solvent was decanted, and the polymer residue was left to dry for 5-10 minutes, after which a small amount of CH_2Cl_2 (2-3 mL) was added to solve the polymer for casting a film. The film was cast into a small Petri dish and left to dry at room temperature overnight, after which the film was removed from the dish for further drying under reduced pressure.

Polymerization of poly(tTa-HDT)

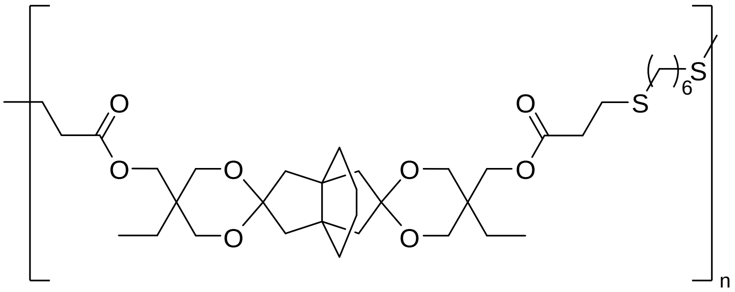

tTa (386.8 mg, 0.73 mmol) was dissolved in 5 mL of $CHCl_3$, HDT (112.1 mg, 114 µl, 0.78 mmol) was added, and the mixture was cooled on an ice bath. DBU (11.9 mg, 0.078 mmol)

was added as a solution in $CHCl_3$, and the ice bath was removed. The mixture was stirred at room temperature for 24 hours. The mixture was then precipitated in 100 mL of MeOH while stirring slowly. The next day, the ether was decanted, the polymer residue solved in CH_2Cl_2 and cast into a Petri dish to obtain a thin film (373.5 g, yield 70.3%).
^{1}H NMR (400 MHz, $CDCl_3$): *d* 4.24, 3.66, 3.55, 2.77, 2.62, 2.52, 2.03, 1.58, 1.40, 1.29, 0.80. (**Fig. S22**)

Polymerization of poly(tTa-PDT)

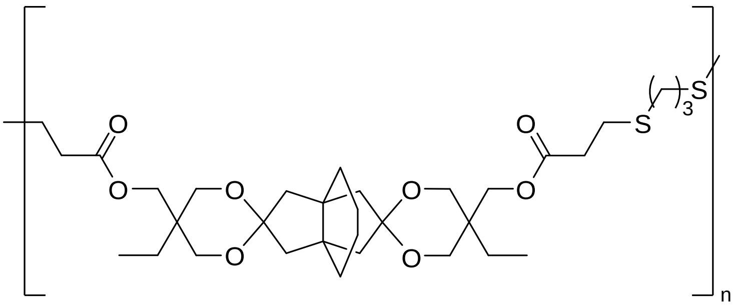

tTa (1.378 g, 2.59 mmol) was dissolved in 10 mL of $CHCl_3$, PDT (288 mg, 267 µl, 2.66 mmol) was added, and the mixture was cooled on an ice bath. DBU (42.6 mg, 0.28 mmol) was added as a solution in $CHCl_3$, and the ice bath was removed. The mixture was stirred at room temperature for 24 hours. The solution was then precipitated in 100 mL of MeOH while stirring slowly. The next day, the ether was decanted, the polymer residue was solved in CH_2Cl_2 and cast into a Petri dish to obtain a thin film (1.383 g, yield 77.6%).
^{1}H NMR (400 MHz, $CDCl_3$): *d* 4.24, 4.05, 3.75, 3.66, 3.59, 3.55, 2.77, 2.63, 2.33, 2.24, 1.99, 1.85, 1.62, 1.41, 1.29, 0.88, 0.81. (**Fig. S23**)

Polymerization of poly(tTa-TBBT)

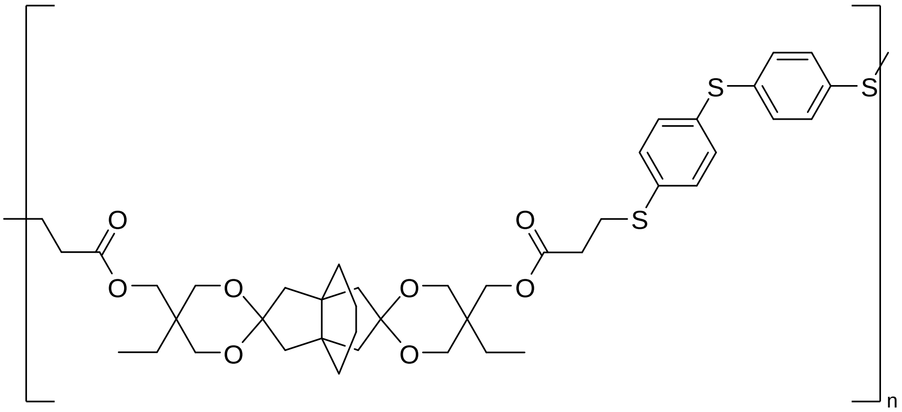

tTa (1.386 g, 2.60 mmol) was dissolved in 10 mL of $CHCl_3$, TBBT (670.8 mg, 2.67 mmol) was added, and the mixture was cooled on an ice bath. DBU (42.9 mg, 0.28 mmol) was added as a solution in $CHCl_3$, and the ice bath was removed. The mixture was stirred at room temperature for 24 hours. The mixture was then precipitated in 100 mL of MeOH while stirring slowly. The next day, the ether was decanted, the polymer residue solved in CH_2Cl_2 and cast into a Petri dish to obtain a thin film (1.665 g, yield 76.7%).
Some of the precipitate (170 mg) remained insoluble in CH_2Cl_2 and was collected separately.
Alternative method in 2-Me-THF
tTa (183.1 mg, 0.34 mmol) was dissolved in 6 mL of 2-MeTHF, TBBT (88.6 mg, 0.35 mmol) was added, and the mixture was cooled on an ice bath. DBU (5.6 mg, 0.03 mmol) was added as a solution in 2-MeTHF, and the ice bath was removed. The mixture was stirred at room

temperature for 24 hours. Thereafter the mixture was then precipitated in 100 mL of MeOH while stirring slowly. The next day, the ether was decanted, the polymer residue solved in CH_2Cl_2 and cast into a Petri dish to obtain a thin film (110.1 mg, yield 38.4%).
^{1}H NMR (400 MHz, $CDCl_3$): *d* 7.40, 7.29, 4.50, 4.24, 4.05, 3.58, 3.48, 3.16, 2.65, 2.24, 1.99, 1.61, 1.40, 1.28, 0.87, 0.80. (**Fig. S24**)

Polymerization of poly(tTma-PDT)

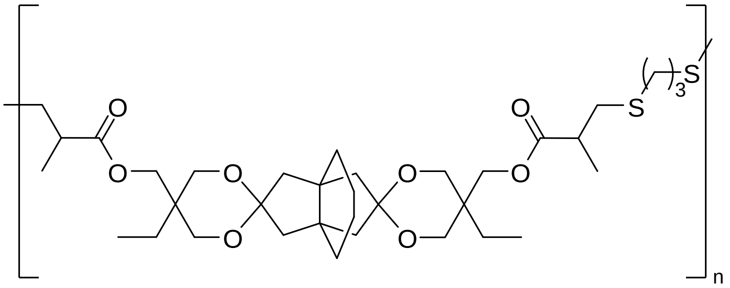

tTma (1.633 g, 2.92 mmol) was dissolved in 6 mL of $CHCl_3$, PDT (324.9 mg, 301 µl, 3.00 mmol) was added, and the mixture was cooled on an ice bath. DBU (48.1 mg, 0.31 mmol) was added as a solution in $CHCl_3$, and the ice bath was removed. The mixture was stirred at room temperature for 24 hours. The mixture was then precipitated in 100 mL of MeOH while stirring slowly. The next day, the ether was decanted, the polymer residue solved in CH_2Cl_2 and cast into a Petri dish to obtain a thin film (1.423 g, yield 67.9%).
^{1}H NMR (400 MHz, $CDCl_3$): *d* 4.23, 4.18, 3.70, 3.51, 2.76, 2.59, 2.23, 1.99, 1.82, 1.49, 1.28, 0.88, 0.80. (**Fig. S25**)

Polymerization of poly(tBa-PDT)

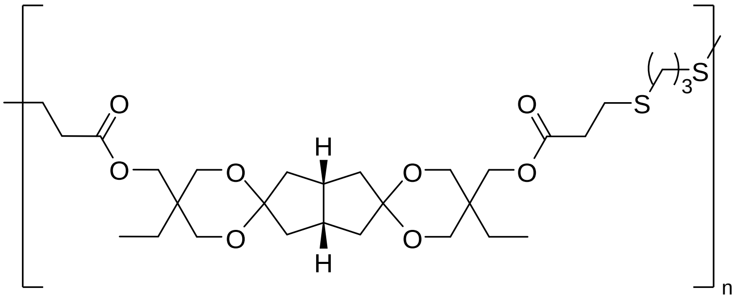

tBa (1.12 g, 2.34 mmol) was dissolved in 12 mL of $CHCl_3$, PDT (260.6 mg, 242 µL, 2.41 mmol) was added, and the mixture was cooled on an ice bath. DBU (39.4 mg, 0.25 mmol) was added as a solution in $CHCl_3$, and the ice bath was removed. The mixture was stirred at room temperature for 40 hours. The mixture was then precipitated twice into 200 mL of MeOH while stirring slowly. The next day, the ether was decanted, and the polymer residue was obtained as a sticky soft yellowish solid (1.11 g, yield 77%).
Alternative method in 2-MeTHF
tBa (459 mg, 0.96 mmol) was dissolved in 10 mL of 2-MeTHF, PDT (101.2 mg, 94 µL, 0.94 mmol) was added, and the mixture was cooled on an ice bath. DBU (15.4 mg, 0.10 mmol) was added as a solution in 2-MeTHF, and the ice bath was removed. The mixture was stirred at room temperature for 48 hours. The mixture was then precipitated twice in 200 mL of MeOH while stirring slowly. The next day, the ether was decanted, and the polymer residue was obtained as a sticky soft yellowish solid (0.459 g, yield 73.6%).
^{1}H NMR (400 MHz, $CDCl_3$): *d* 4.24, 3.65, 3.57, 2.76, 2.61, 2.51, 2.26, 2.08, 1.84, 1.70, 1.30, 0.80. (**Fig. S26**)

Polymerization of poly(gBa-PDT)

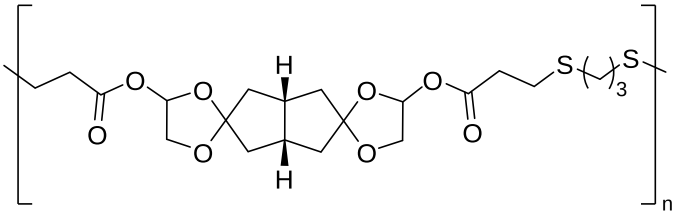

gBa (0.892g, 2.26 mmol) was dissolved in 10 mL of DCM, PDT (259.5 mg, 241 µl, 2.33 mmol) was added, and the mixture was cooled on an ice bath. DBU (38.6 mg, 0.25 mmol) was added as a solution in DCM, and the ice bath was removed. The mixture was stirred at room temperature for 40 hours. The mixture was then precipitated twice in 200 mL of Et_2O while stirring slowly. The next day, the ether was decanted, and the polymer residue was obtained as a soft solid (0.83 g, yield 70.0%).
^{1}H NMR (400 MHz, $CDCl_3$): *d* 4.24, 4.12, 3.99, 3.70, 2.77, 2.62, 1.96, 1.84, 1.72. (**Fig. S27**)

NMR analysis

Glycerol spirodiol gB and diacrylate gBa NMR analysis

Ketalization of *cis*-bicyclo[3.3.0]octane-3,7-dione **B** with glycerol results in numerous isomers. At first the formation of 1,2 or 1,3 ketals is possible. In first case hydroxymethyl group can be connected to *endo* or *exo* position of C3 and C7 of *cis*-bicyclooctane ring and further isomers are obtained from the mutual different orientation of hydroxymethyl groups in diketals. ^{1}H NMR spectrum at 800 MHz is non-informative about the composition of mixture of compounds (Fig. S1). It is hard to resolve even numerous first order multiplets from 4-CH_2OH substituted 1,3 dioxolane ring. For example, the number of signals from the vicinal couplings of H-4 between 4.00 and 3.95 ppm with neighbor methylene protons must be 128. ^{13}C NMR spectrum of ketalization product reveals the formation of complex mixture of compounds. For ^{13}C NMR spectrum the most informative starting points are the regions of spiro carbons with connected to them two carbon and two oxygen atoms. For the naming of these isomers generic names were used (see Fig. S2)
In principle spiro connected to bicyclo[3.3.0]octane hydroxymethyl group at C4 of 1,3-dioxolane ring can have 2 different configurations, but they are barely observed due to low barrier conformational mobility of 1,3-dioxolane ring. Geometry optimizations by AM1 and Gaussian calculations show that these isomers differ in their energies in the order of only 100 cal/mol and have in most stable conformation diversely twisted bicyclo[3.3.0]octane 5-membered rings which are characterized also by the different dihedral angles between the bicyclo[3.3.0]octane bridgehead H atoms. Different calculations give these angles values from nearly zero to more than 30 degrees. The 1,3-dioxolane parts of isomers are characterized by a low inversion barrier of conversion from the different mutual orientation of substituents on the 1,3-dioxolane ring. No NMR study of this conversion was found, but an ESR study from 1973 has found that the inversion barrier in 2-methyl 1,3-dioxolane is as low as 5.6±0.2 kcal/mol[1]. This needs temperatures below -100 °C degrees to observe different conformers in NMR spectra. Room temperature linewidths in present mixture are quite narrow to resolve 0.003 ppm differences in ^{13}C chemical shifts, but at the same time they already demonstrate the small exchange broadening effects. This is seen in the ^{13}C spectrum of bicyclo[3.3.0]octane bridgehead carbons in a mixture of glycerol di-and monoketals at room temperature (Fig. S4). Resolution enhancement reveals the presence of dynamic broadening in signals from diketals. The monoketal itself is also not free from the exchange effects, because the keto ring signals are even sharper compared to the other monoketal signals. The number of observed isomers

led to the conclusion that mutual orientation of substituents in 1,3-dioxolane ring isomers are still separable in NMR spectra. Additionally, *exo* and *endo* substitution *cis* and *trans* orientations of hydroxymethyl substituents were observed. For further analysis the configuration of one hydroxymethyl group was fixed and remaining substitution patterns were fixed toward this substituent. The analysis of 6 isomeric diketals was based on NMR spectra of glycerol monoketals and 2,2-dimethyl-1,3-dioxolan-4-yl-methanol (solketal, Fig. S3)
^{13}C NMR spectrum of monoketals shows the presence of 2 compounds defined as *exo* and *endo* isomers with the chemical shift differences between the corresponding atoms from 0.01 to 0.5 ppm. The largest difference is observed on methylene groups of 1,3-dioxolane ring due to their *exo* or *endo* orientation on bicyclo[3.3.0] ring in beta position from spiro carbon. As a model compound for the assignment of *exo* or *endo* methylene groups the chemical shifts of 3-methoxy isomers of *cis*-bicyclooctane derivatives were used[2]. In this study *endo* methoxy carbons on C3 of bicyclooctane were shifted to low field. The same regularity is observed also for 1,3-dioxolane ring methine carbon atoms in present isomers. Further confirmation of assignment of *exo* or *endo* configuration of hydroxymethyl substituents follows from ^{1}H chemical shift differences of bicyclo[3.3.0]octane bridgehead proton chemical shifts, which result from long range deshielding effects of CH_2OH groups in *exo* isomers by shifting bridgehead protons to low fields by about 0.02 ppm. Very small ^{1}H chemical shift differences in two monoketal isomers complicate the use of NOESY experiments for the analysis of interactions between the spiro and bicyclooctane ring protons in these isomers.
Another model compound, solketal behaves differently from the 2-methyl-1,4-dioxaspiro[4.5]decane with 4-methylsubstituted 1,3-dioxolane ring. For the last compound half chair conformation was declared on the basis of vicinal H-H spin-spin coupling constants with methine proton as 5.7 and 8.4 Hz[3]. In solketal and in present monoketal and diketals these coupling constants have very similar values (in solketal 6.5 and 6.6 Hz in $CDCl_3$ and 6.3 and 6.4 Hz in DMSO, in both monoketals 6.7 and 6.3 Hz in $CDCl_3$ and 6.5 and 6.1 Hz in DMSO). These results justify the use of solketal as adequate model for the analysis of present isomers. Methyl atoms on C2 of solketal have different ^{1}H and ^{13}C chemical shifts. These chemical shifts were assigned by NOESY experiments, which show that both proton and carbon chemical shifts are for methyl groups *cis* oriented to hydroxymethyl group shifted towards low fields. This result is in accordance with ^{13}C NMR studies of stereoisomeric 2,4-dialkyl 1,3-dioxolanes[4]. Full assignment of ^{13}C chemical shifts in monoketals was achieved by ^{13}C-^{13}C INADEQUATE correlation experiments. This results in assignment of connections between the bridgehead and methylene carbons of bicyclo[3.3.0]octane ring, which are important for the assignment of *cis* and *trans* isomers of unsymmetrical *endo-exo* isomers. Correlations between the bridgehead carbons were not observed due to too low intensities of outer signals of AB spin systems.
With the information from solketal and monoketals the diketal mixture was analyzed by various 2D FT experiments (COSY, NOESY, HSQ, HMBC, SELECTIVE HMBC, INADEQUATE). Spectra were measured in $CDCl_3$, MeOD and DMSO-d_6. Best resolution of bicyclo[3.3.0]octane bridgehead protons was observed in DMSO solution (Fig. S1), being complex band of overlapping signals, but still giving possibility to assign by 2D FT bridgehead 10 carbon signals to definite isomers (Fig. S4). INADEQUATE experiment was used to sort out signals to all isomers. In Fig. S5 the connectivity diagram of bridgehead carbons is demonstrated and in Fig. S6 the assignment of methylene carbon atoms in 6 isomers is shown.
Acrylic acid diesters from the mixture of spirodiols have retained the same relative concentrations of 6 the isomers. Expanded ^{13}C NMR spectrum is quite similar to the spectrum of diols (Fig. S7). In ^{13}C typical NMR esterification effects are observed in alcohol parts of isomers where in alpha position regular ~2 ppm low field and in beta position ~3 ppm high field shifts are registered. At more remote positions different types of carbon atoms are shifted

marginally to higher fields. Terminal acrylic carbons are not now any more separated to 8 components and carbonyl carbons show 2 signals representing only *exo* and *endo* orientation towards bicycle C3 and C7. Esterification of spirodiols results in smaller variations of carbon chemical shifts within bicyclo[3.3.0]octane bridgehead carbon chemical shifts. In diester they occupy less than 0.30 ppm, in spirodiols they have 0.50 ppm range.
The most surprising result in ^{1}H NMR is the resolution of vinyl protons to 6 from possible 8 types. In Fig. S8 ^{1}H signals from high field half of terminal Z-vinyl protons with only geminal 1.4 Hz coupling constants are shown. These chemical shift differences are result of 22 bond distance between the terminal vinyl H atoms in these isomeric acrylic acid diesters.

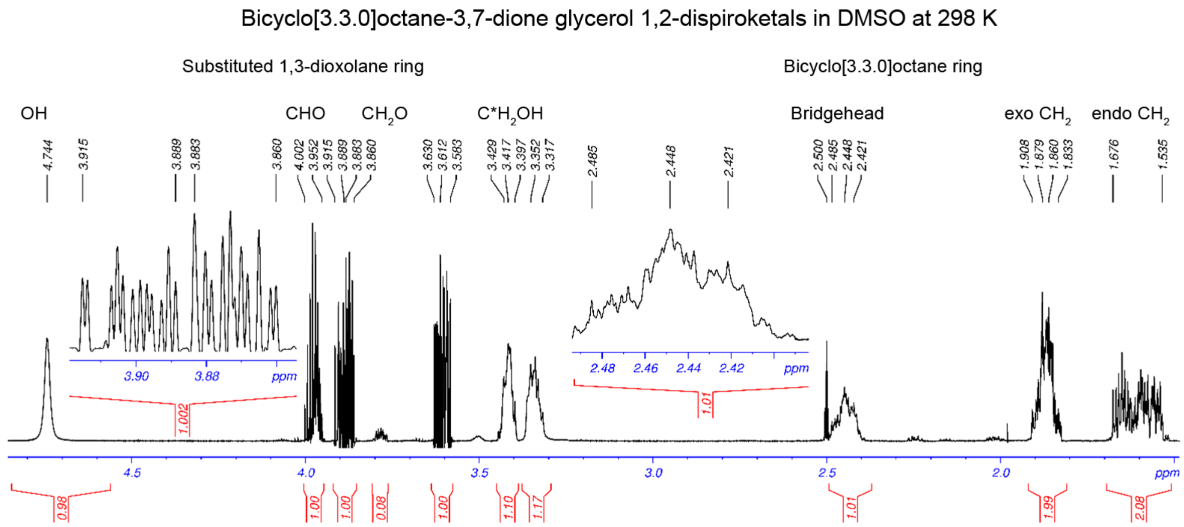

Fig. S1. *Room temperature 800 MHz ^{1}H NMR spectrum from isomeric dispirodiols mixture in DMSO solution*

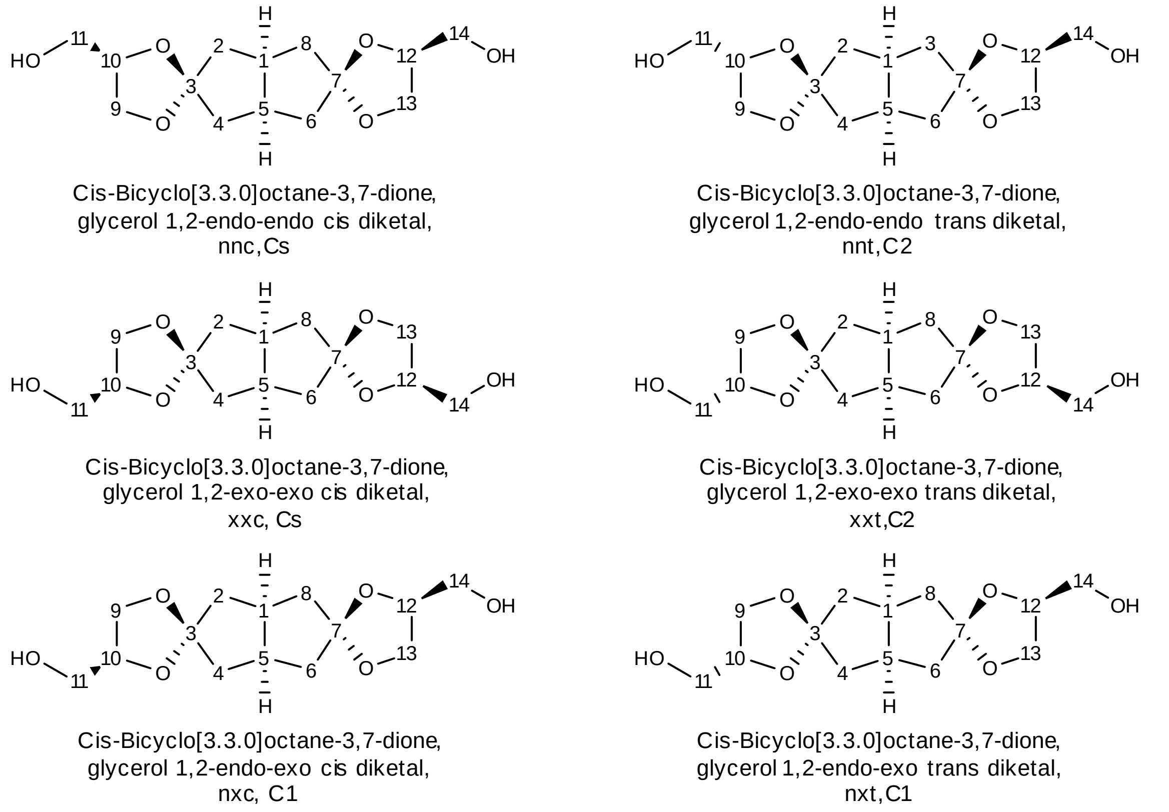

Fig. S2. *Isomeric diketals, obtained by glycerol 1,2-ketalization of cis-bicyclo[3.3.0]octane-3,7-dione with their generic names, used abbreviations and symmetry point groups.*

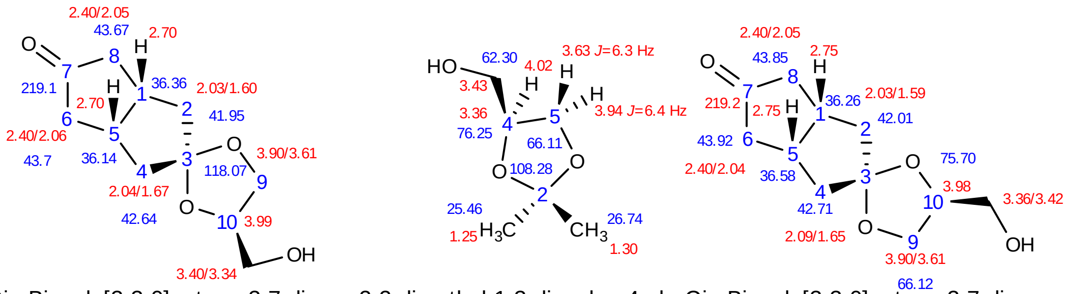

Cis-Bicyclo[3.3.0]octane-3,7-dione, glycerol 1,2-endo monoketal, C1

2,2-dimethyl-1,3-dioxolan-4-yl-methanol, solketal

Cis-Bicyclo[3.3.0]octane-3,7-dione, glycerol 1,2-exo monoketal, C1

Fig. S3. *NMR of model compounds in DMSO at 298 K used for the analysis of NMR spectra of bicyclo[3.3.0]octane-3,7-dione glycerol diketals.*

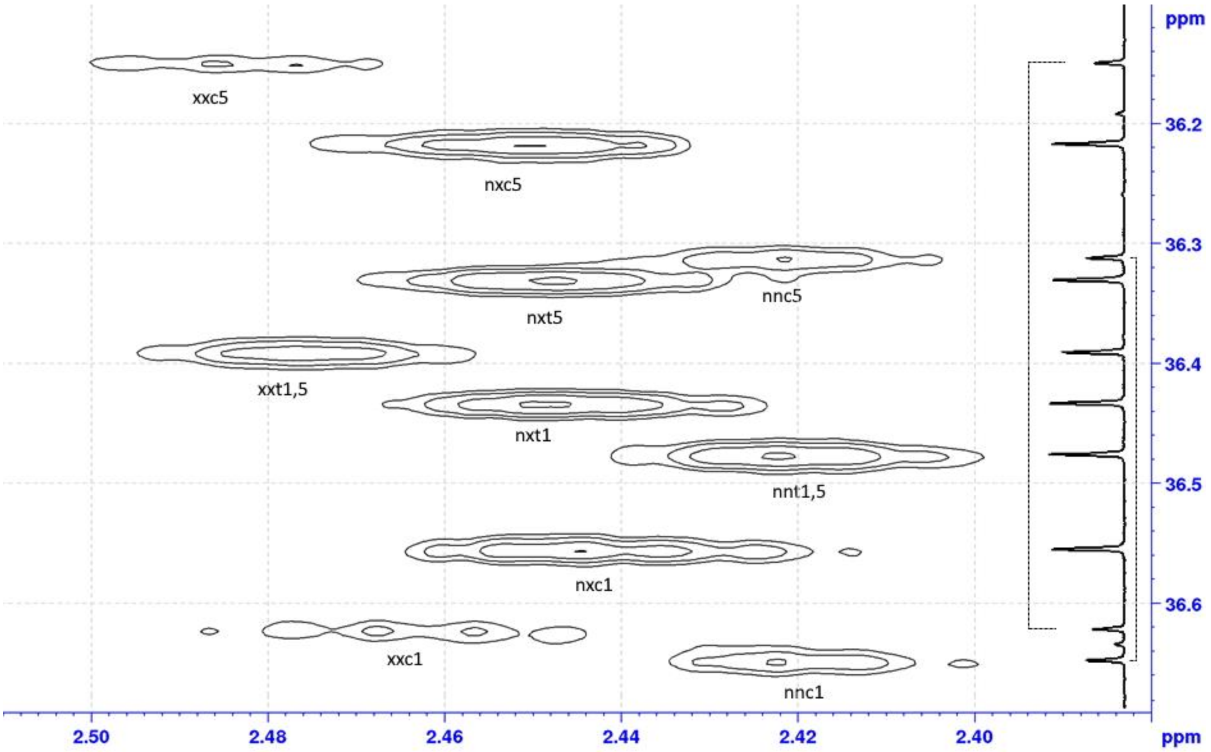

Fig. S4. *Assignment of bicyclo[3.3.0]octane bridgehead ^{1}H and ^{13}C chemical shifts from 2D HSQ experiment. Abbreviations: x- exo, n- endo, c- cis, t- trans, followed by carbon number from* ***Fig. S2****.*

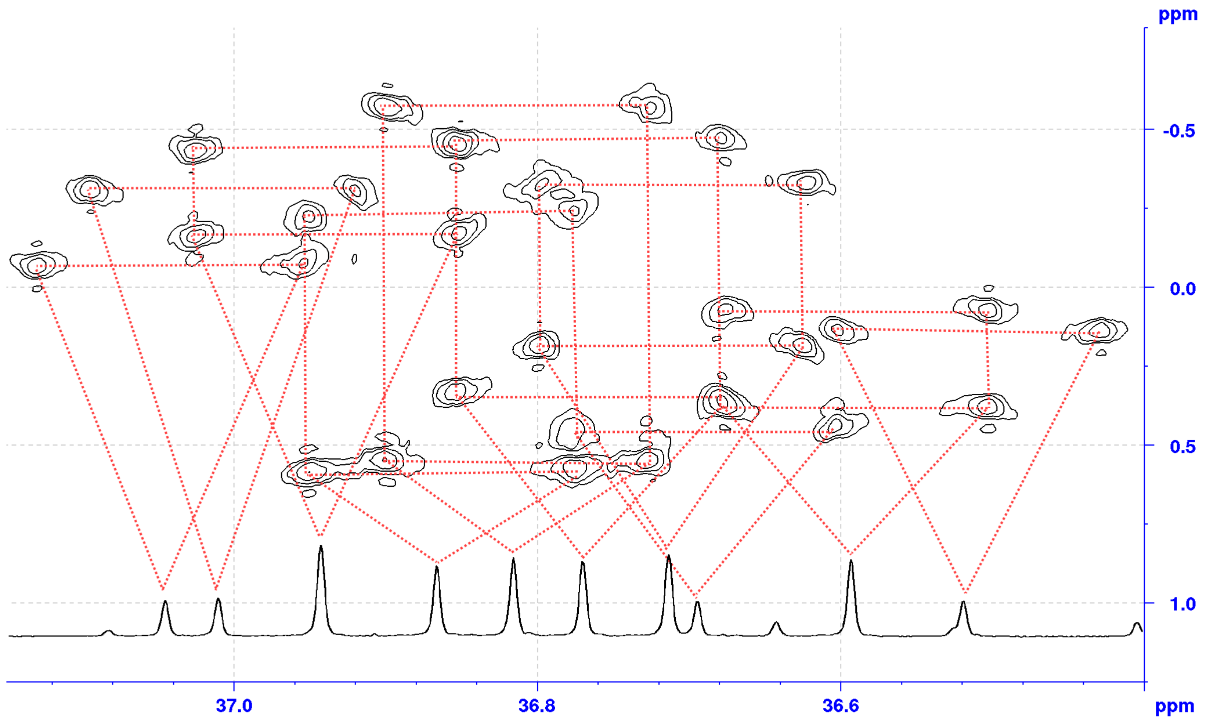

Fig. S5. *Part of INADEQUATE 2D double quantum transfer experiment showing direct connections of bicyclooctane bridgehead to methylene carbon atoms.*

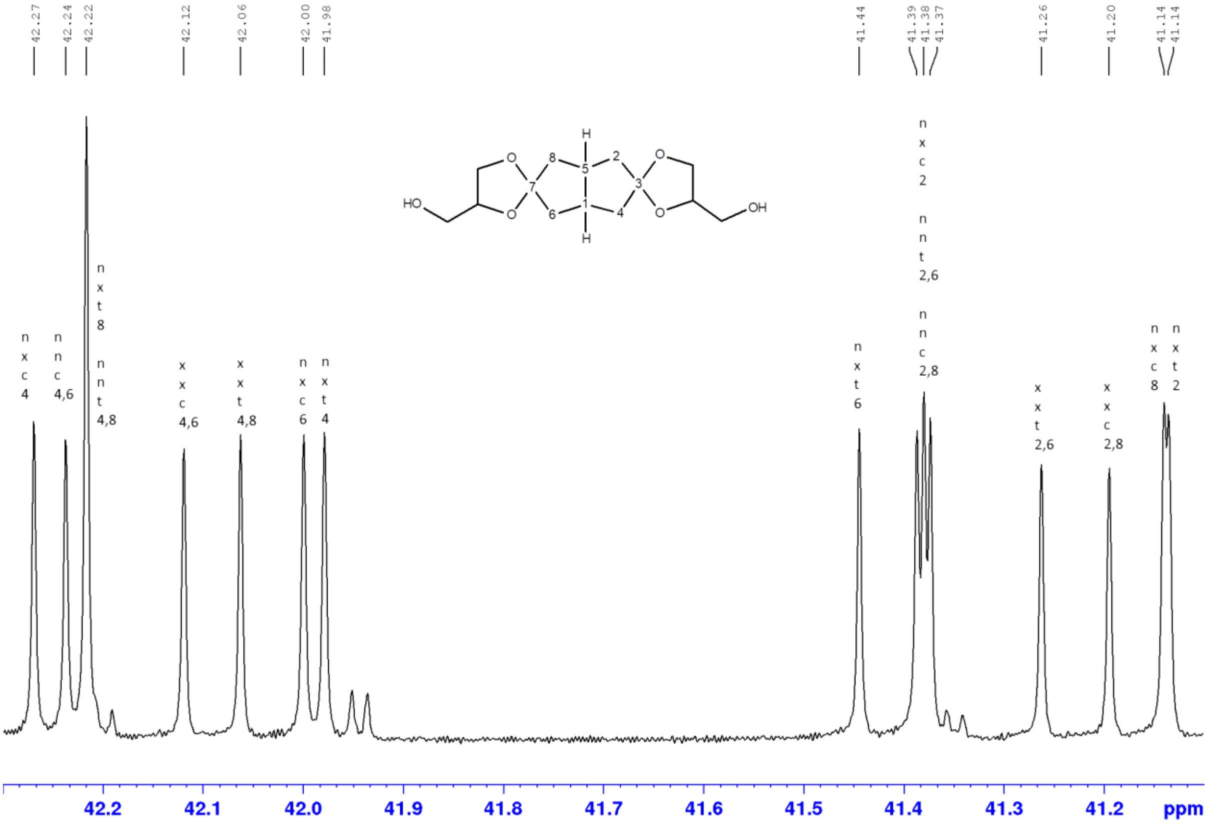

Fig. S6. Assignment of bicyclo[3.3.0]octane ring methylene carbon signals with the help of 2D INADEQUATE experiment. Abbreviations from ***Fig. S2.***

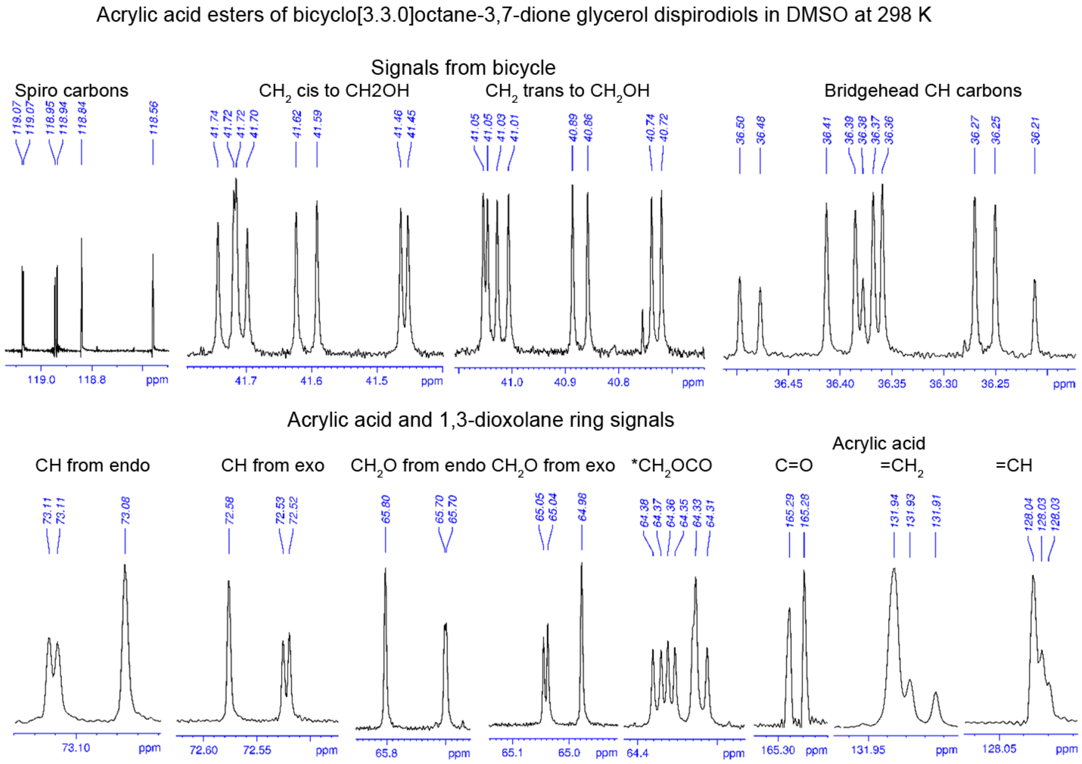

Fig. S7. *Expanded 800 MHz* [13]*C NMR spectrum of acrylic acid esters of from spirodiols mixture.*

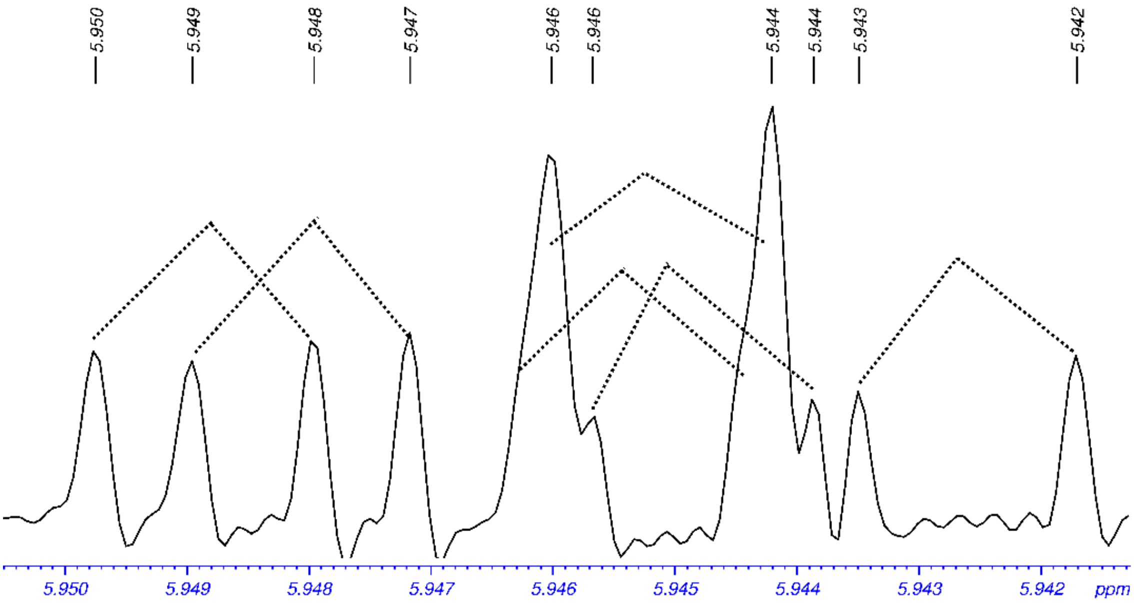

Fig. S8. *High field half of terminal Z proton signals from diacrylate dispirodiols mixture. Doublets are from geminal 1.4 Hz coupling between the terminal protons.*

[4.3.3]propellane spirodiol tT NMR analysis

^{1}H spectrum of **tT** points to dynamic effects in the molecule. Two bands from carbocyclic six-membered ring protons at 1.4 ppm (Fig. S11) are not unresolved equatorial and axial protons, but they are result of intramolecular exchange process. This exchange is even better seen in ^{13}C spectrum (Fig. S10) from the observed linewidths, where signals from all 5 rings of these molecules are influenced. Linewidths in this spectrum reflect the chemical shift differences in exchanging positions of molecules. They are smallest in quaternary carbons resulting in their opposite to normal most intensive signal intensities.

In reported NMR data for unsubstituted [4.3.3]propellane 2 singlet signals with intensity ratio 2 to 3 at 1.40 and 1.58 ppm were reported for ^{1}H at 80 MHz and assigned ^{13}C chemical shifts fit with present data of isomeric propellanes except needed obvious exchange of assignment of six membered ring C2, C5 and five membered rings methylene groups signals. Nothing was reported about intramolecular exchange processes for [4.3.3]propellane. The simplest model compound for dynamics study should be 1,1,2,2-tetramethyl cyclohexane, but data for inversion barrier in this compound were not available. For 1,1-dimethyl cyclohexane experimental NMR studies have reported for ΔG of 10.2[5] and 10.5[6] kcal/mol. These values are very close to reported values on unsubstituted cyclohexane.[7] Thus the observed exchange broadening is specific to present isomers. Our NMR probehead was not suitable for low temperature experiments where temperatures lower than -50 °C are needed. AM1 calculations show that *trans* isomer is more stable by 90 cal/mol and in both isomers the dihedral angle at bridgehead in 6-membered carbocycle is 36.6 degrees. In NMR spectra all methylene protons with 14.4 Hz geminal spin-spin coupling constants in 5-membered rings resonate within 0.07 ppm. For methylene carbons this interval is nearly 100 times larger, demonstrating the advantages of ^{13}C NMR spectroscopy in stereochemical studies.

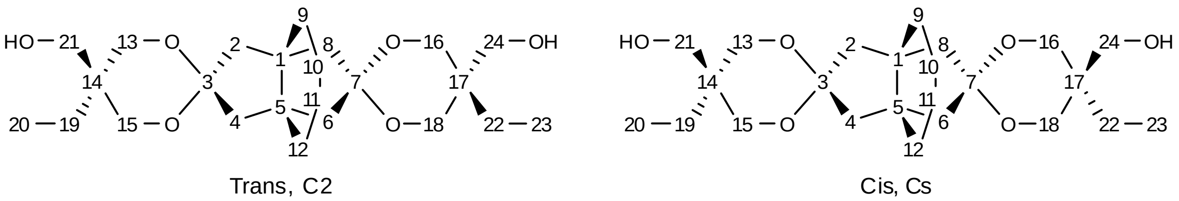

Fig. S9. *Tricyclo[4.3.3.0$^{1.6}$]dodecane-8,11-dione trimethylolpropane diketal cis and trans isomers.*

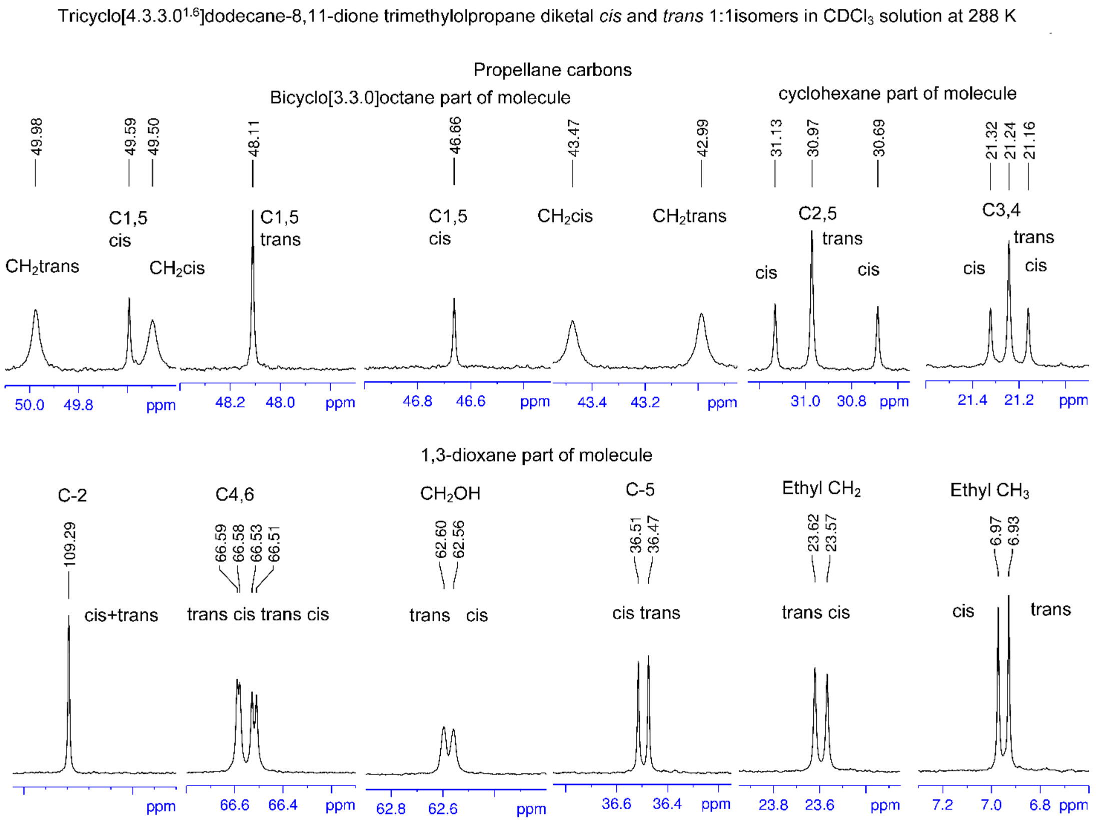

Fig. S10. *Expanded* ^{13}C *NMR spectrum of tricyclo[4.3.3.0*$^{1.6}$*]dodecane-8,11-dione trimethylolpropane diketal cis and trans 1:1 isomers in* $CDCl_3$ *solution at 288 K. Equal horizontal scaling is used for subspectra and vertical scaling demonstrates different linewidths from the inversion exchange processes.*

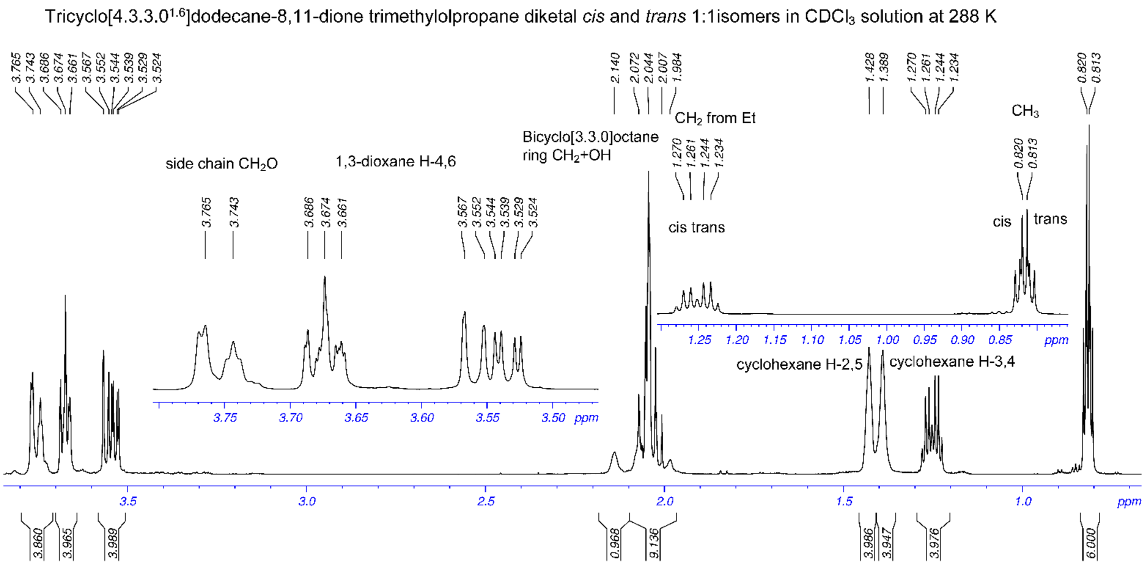

Fig. S11. ^1H *800 MHz NMR spectrum of tricyclo[4.3.3.0*$^{1.6}$*]dodecane-8,11-dione trimethylolpropane diketal cis and trans 1:1 isomers in* $CDCl_3$ *solution at 288 K.*

NMR Spectra

Monomer ^{1}H and ^{13}C NMR spectra

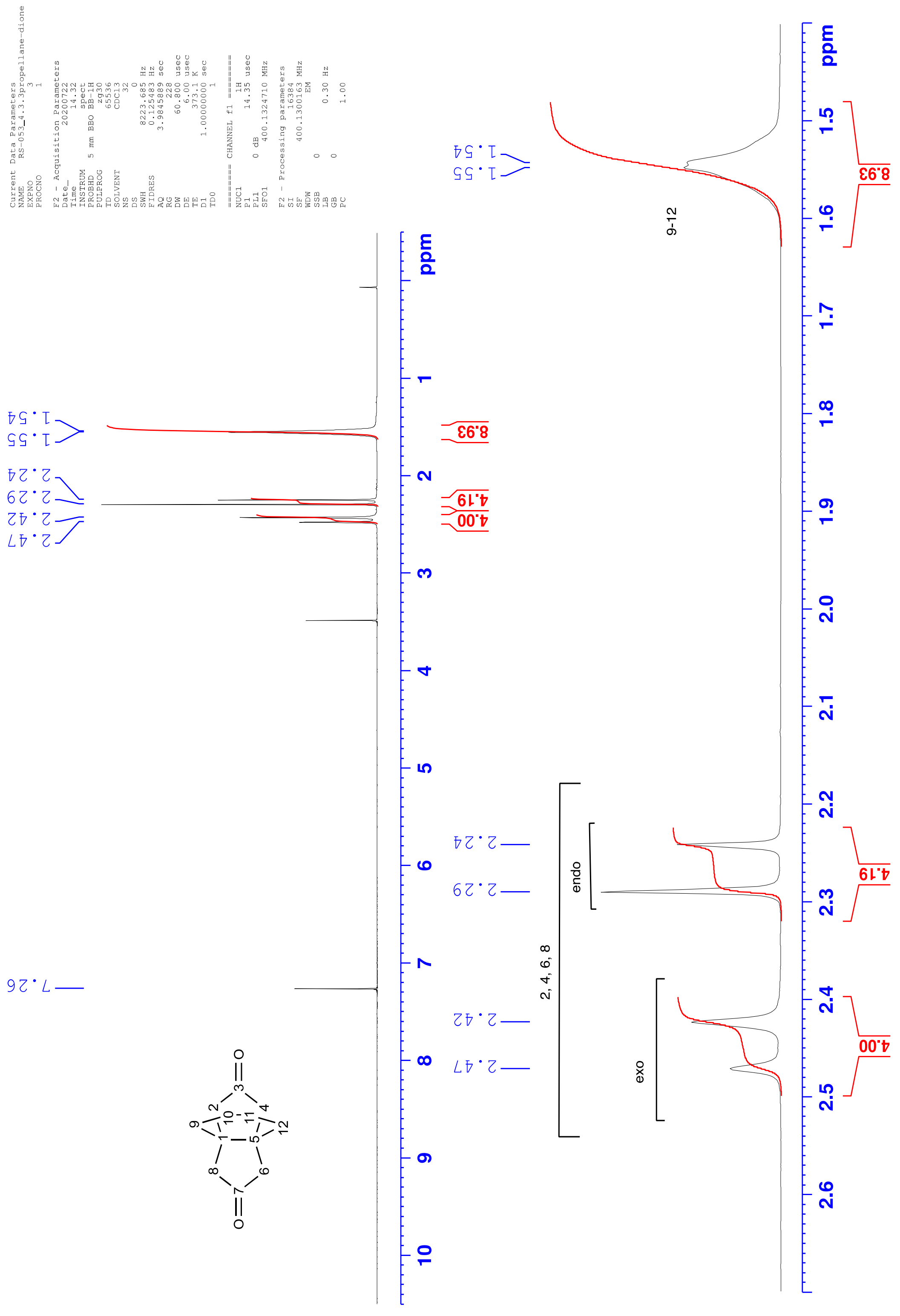

Fig. S12. *400 MHz ^{1}H spectrum of [4.3.3]propellane-8,11-diketone **T** in $CDCl_3$.*

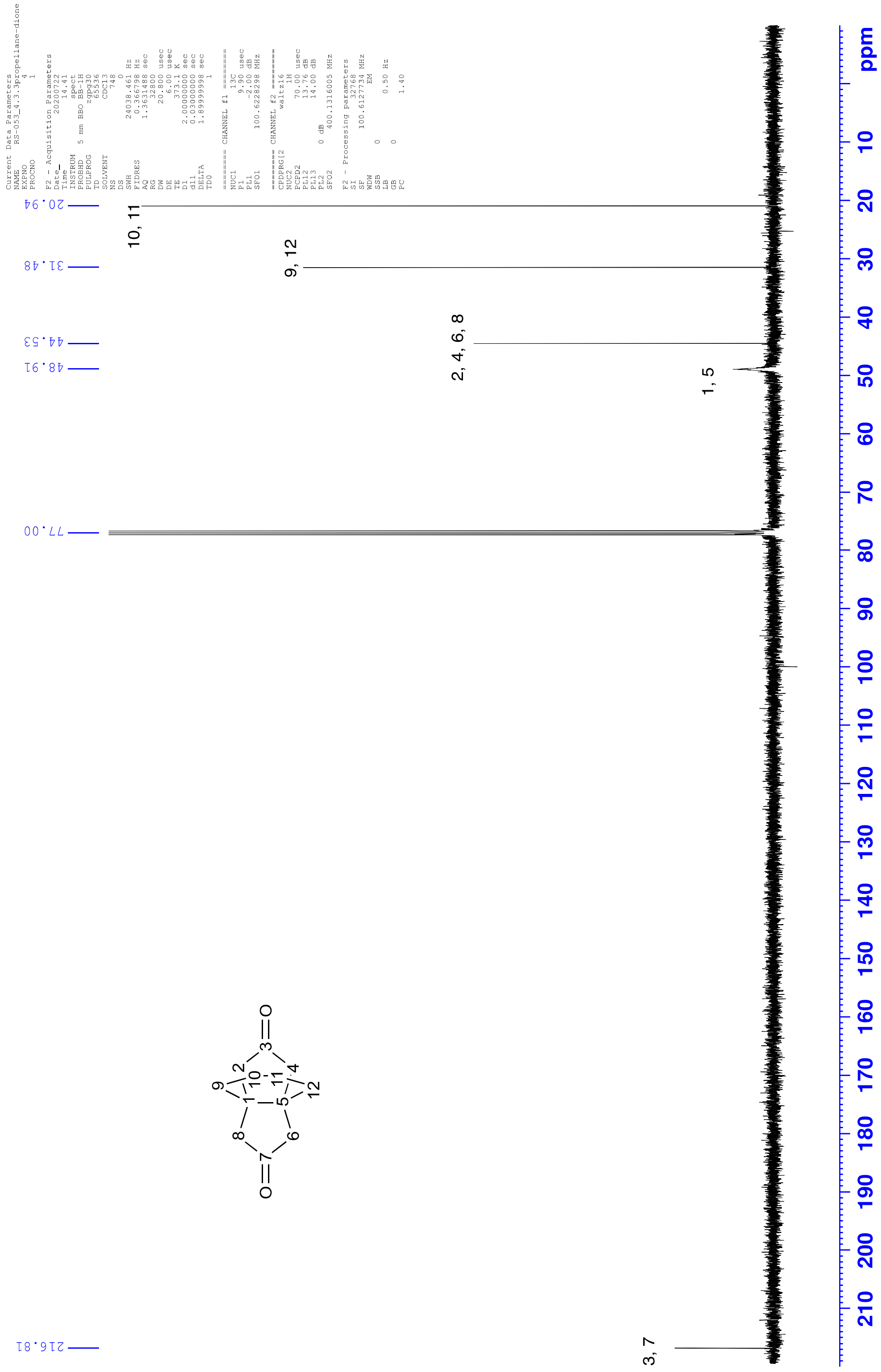

Fig. S13. *100 MHz* ^{13}*C spectrum of [4.3.3]propellane-8,11-diketone* ***T*** *in* $CDCl_3$.

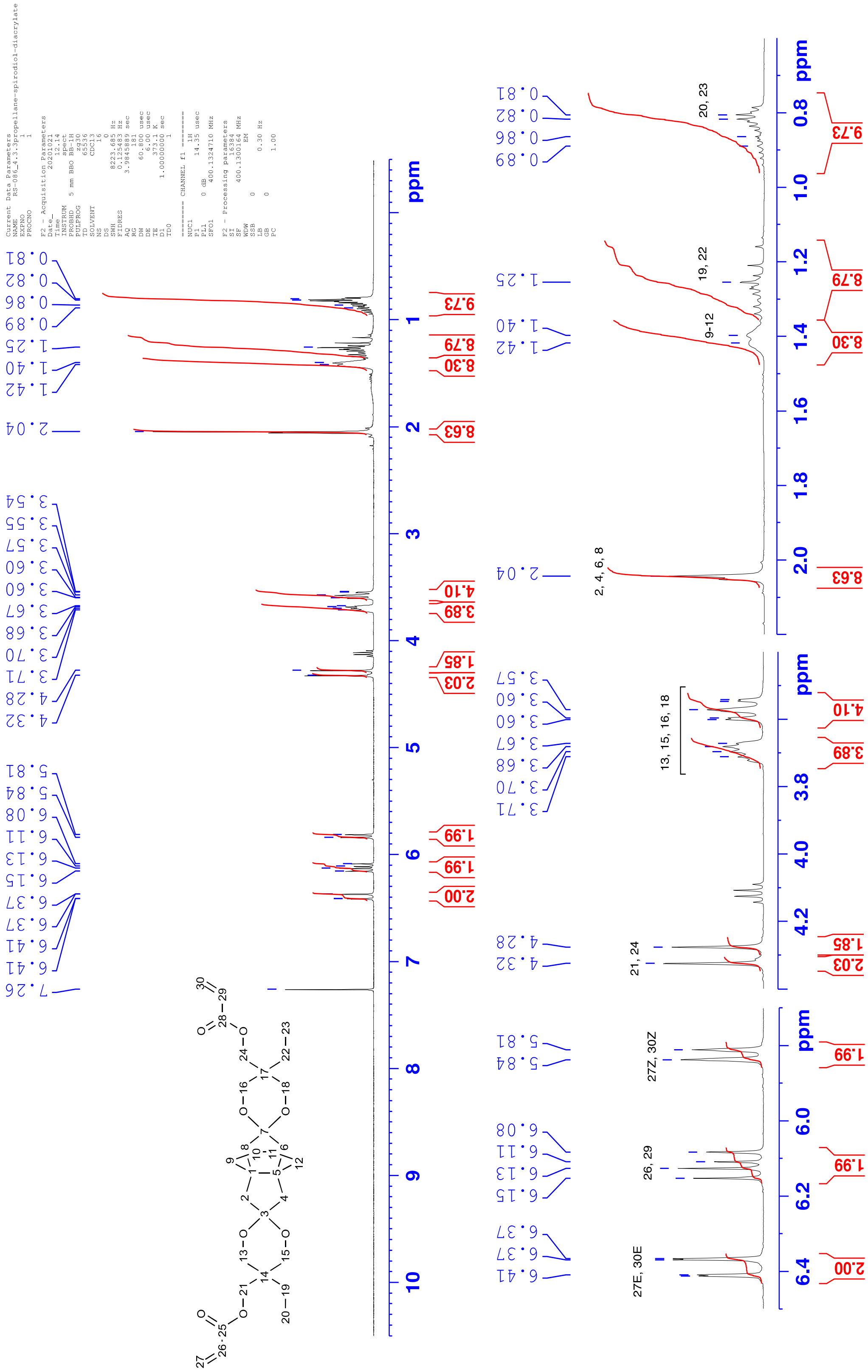

Fig. S14. *400 MHz* [1]*H spectrum of [4.3.3]propellane-spiro-diacrylate* ***tTa*** *in* $CDCl_3$.

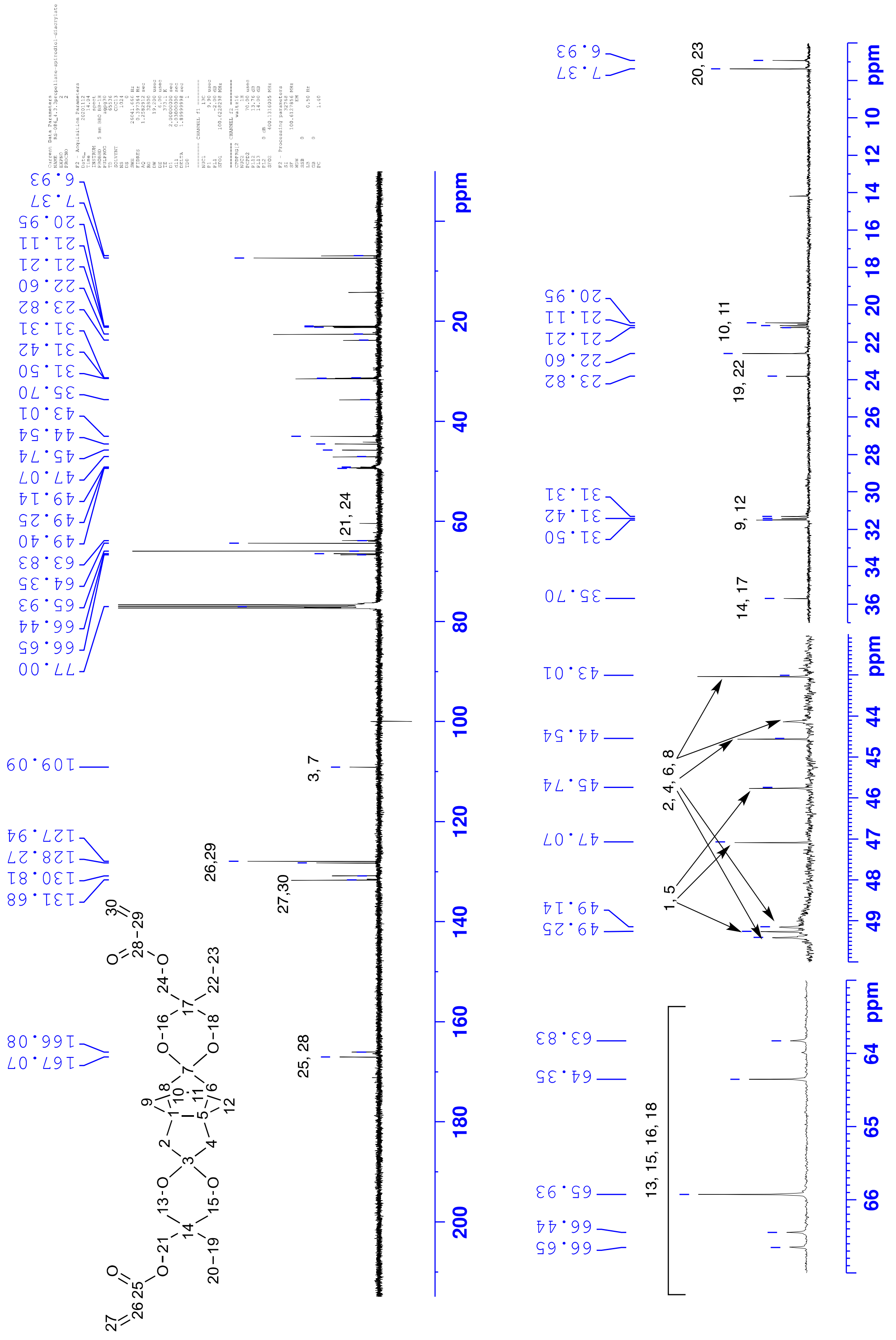

Fig. S15. *100 MHz ^{13}C spectrum of [4.3.3]propellane-spiro-diacrylate **tTa** in $CDCl_3$.*

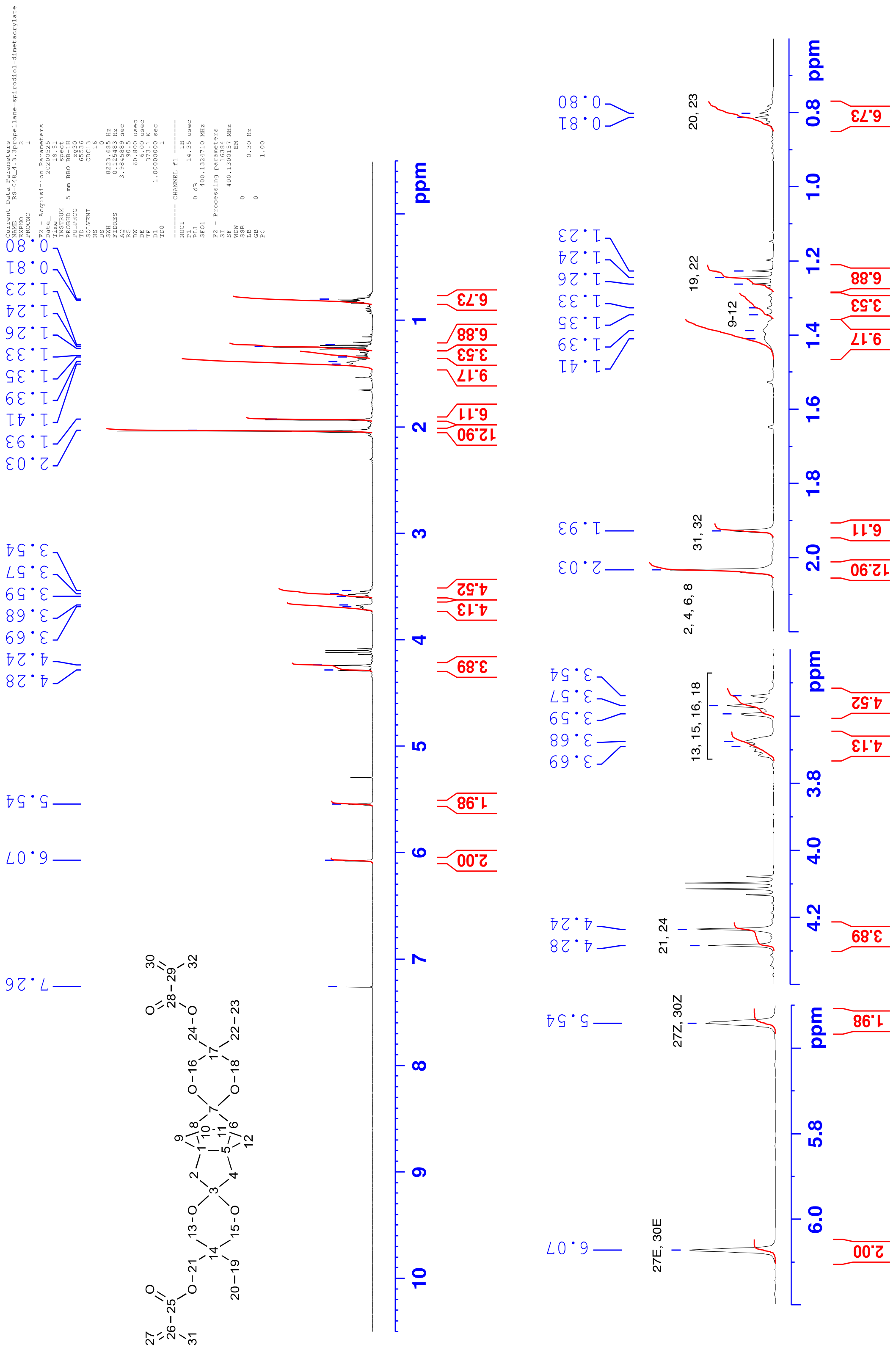

Fig. S16. *400 MHz ^{1}H spectrum of [4.3.3]propellane-spiro-dimethacrylate* ***tTma*** *in $CDCl_3$.*

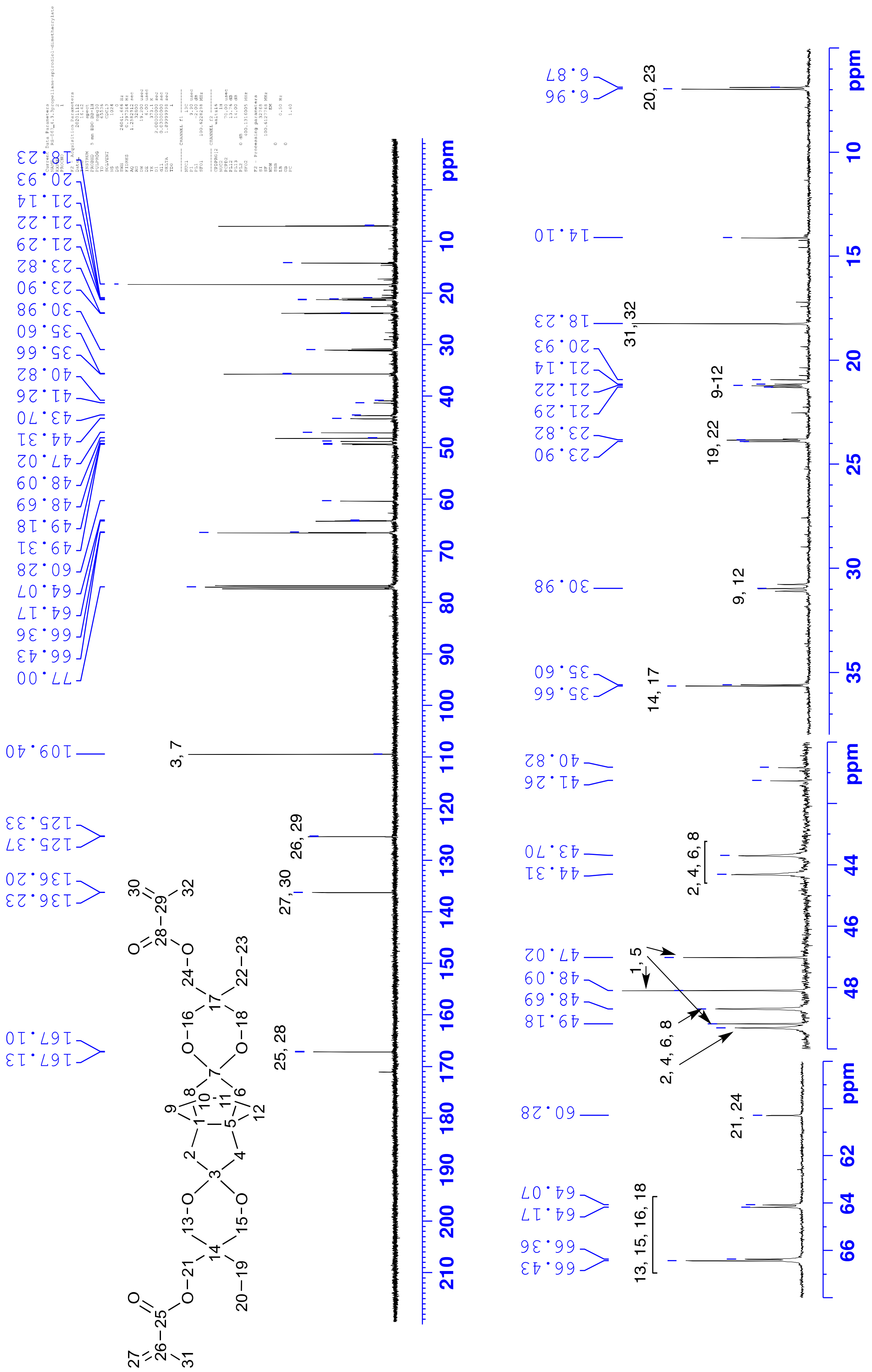

Fig. S17. *100 MHz* ^{13}*C spectrum of [4.3.3]propellane-spiro-diacrylate* ***tTma*** *in* $CDCl_3$*.*

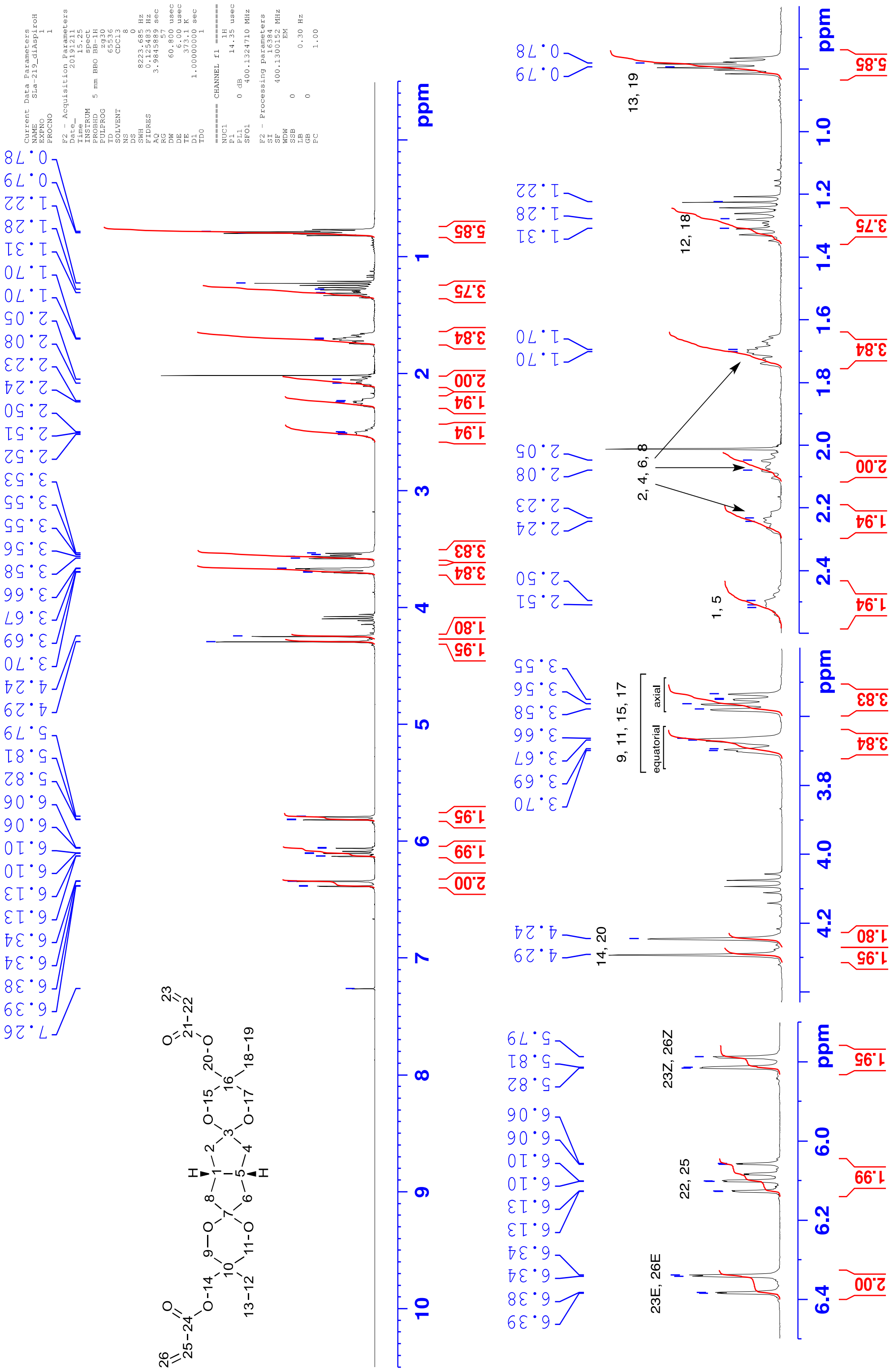

Fig. S18. *400 MHz* ^{1}H *spectrum of [3.3.0]spiro-diacrylate* ***tBa*** *in* $CDCl_3$.

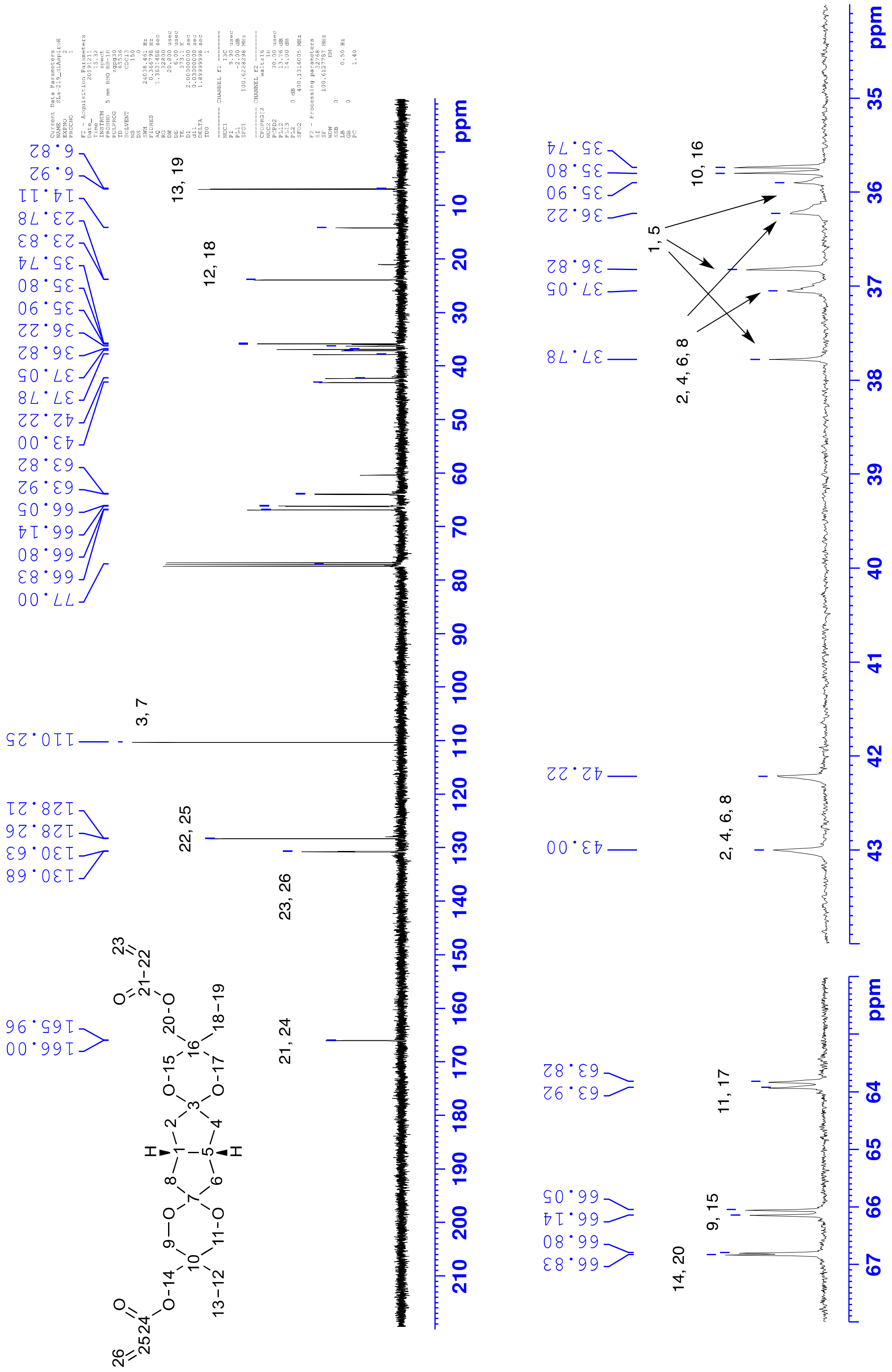

Fig. S19. *100 MHz* ^{13}*C spectrum of [3.3.0]spiro-diacrylate* ***tBa*** *in* $CDCl_3$.

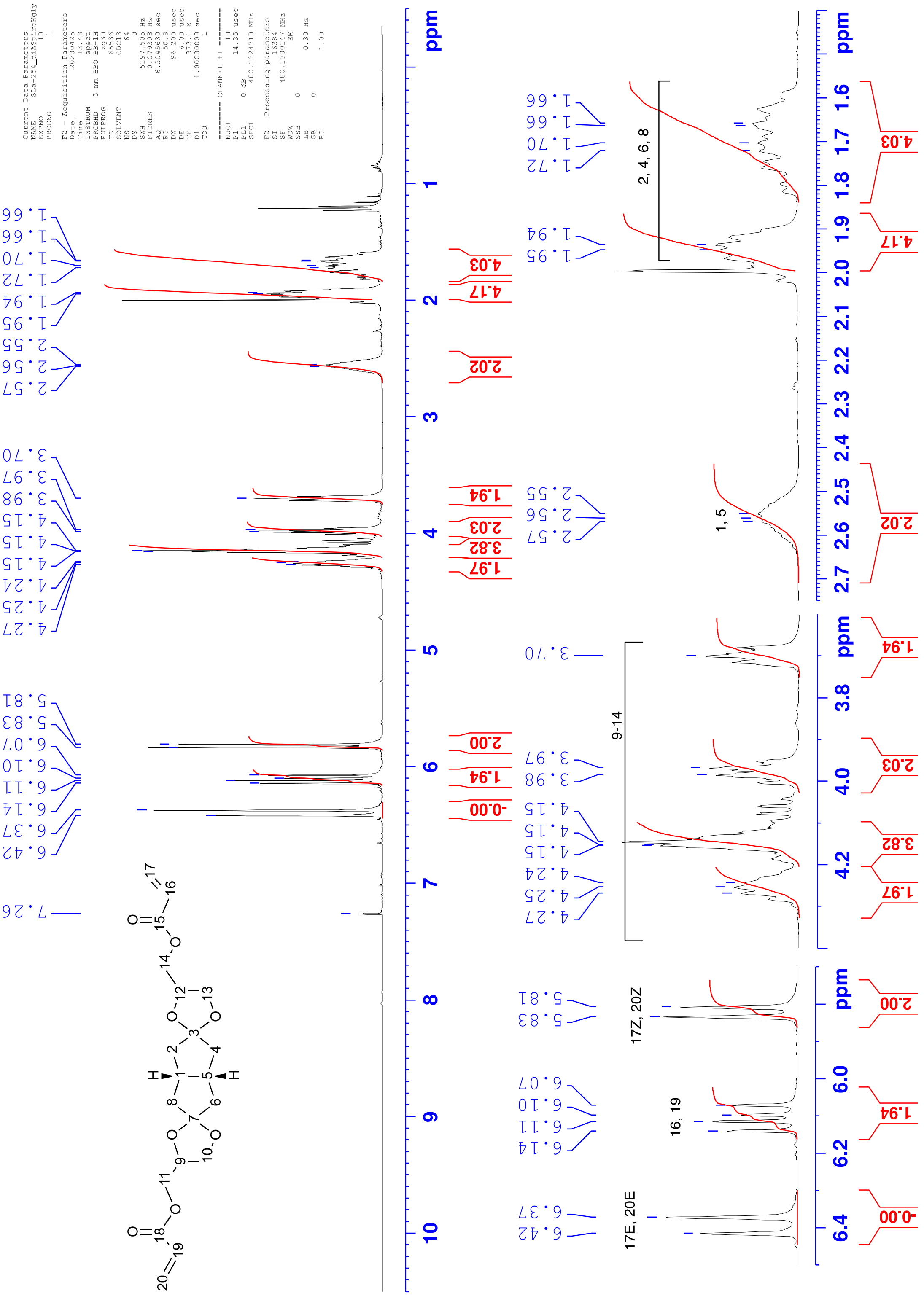

Fig. S20. *400 MHz* ^{1}*H spectrum of [3.3.0]glycerol-spiro-diacrylate* ***gBa*** *in* $CDCl_3$.

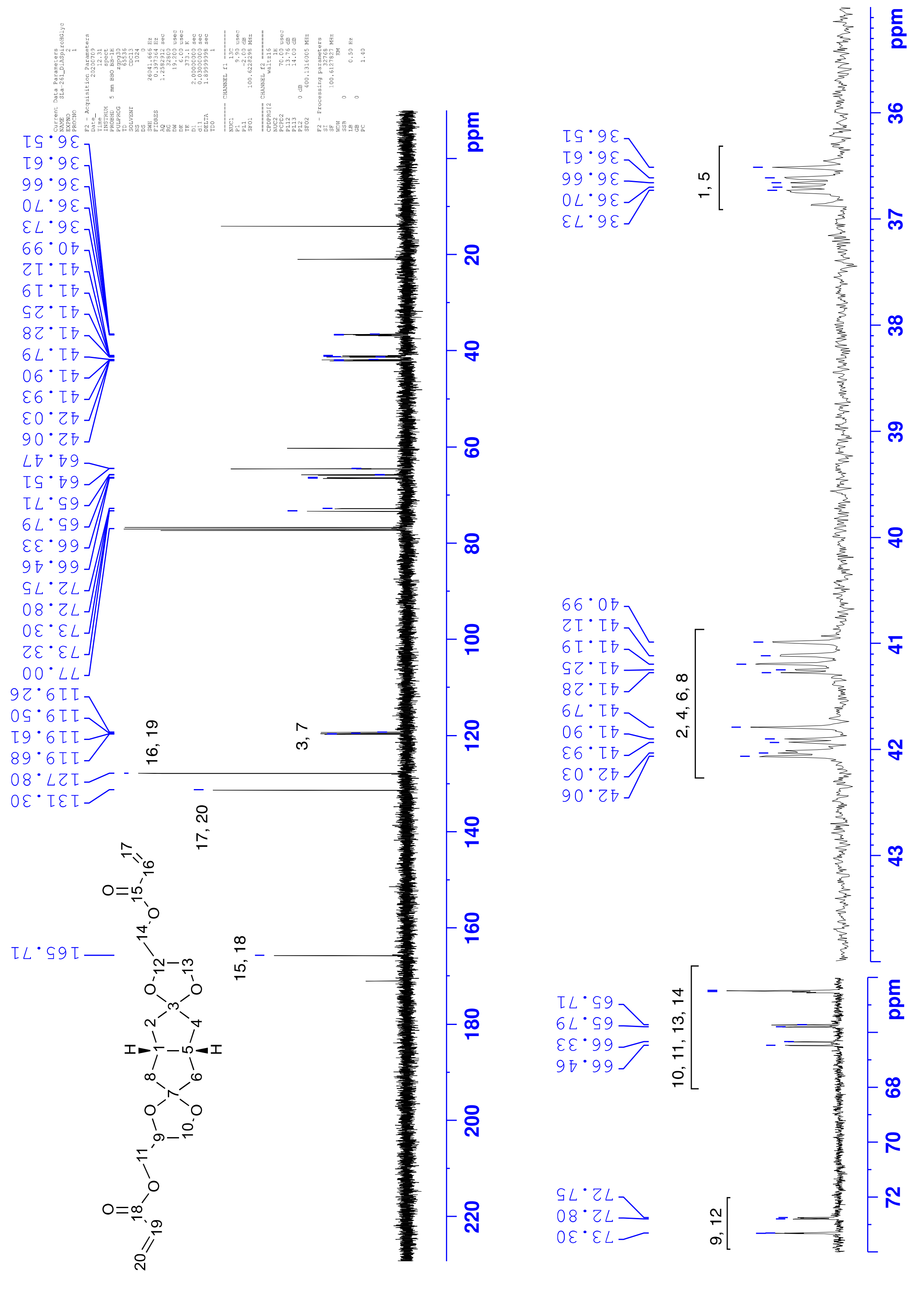

Fig. S21. 100 MHz ^{13}C spectrum of [3.3.0]glycerol-spiro-diacrylate **gBa** in $CDCl_3$.

Polymer ^{1}H NMR spectra

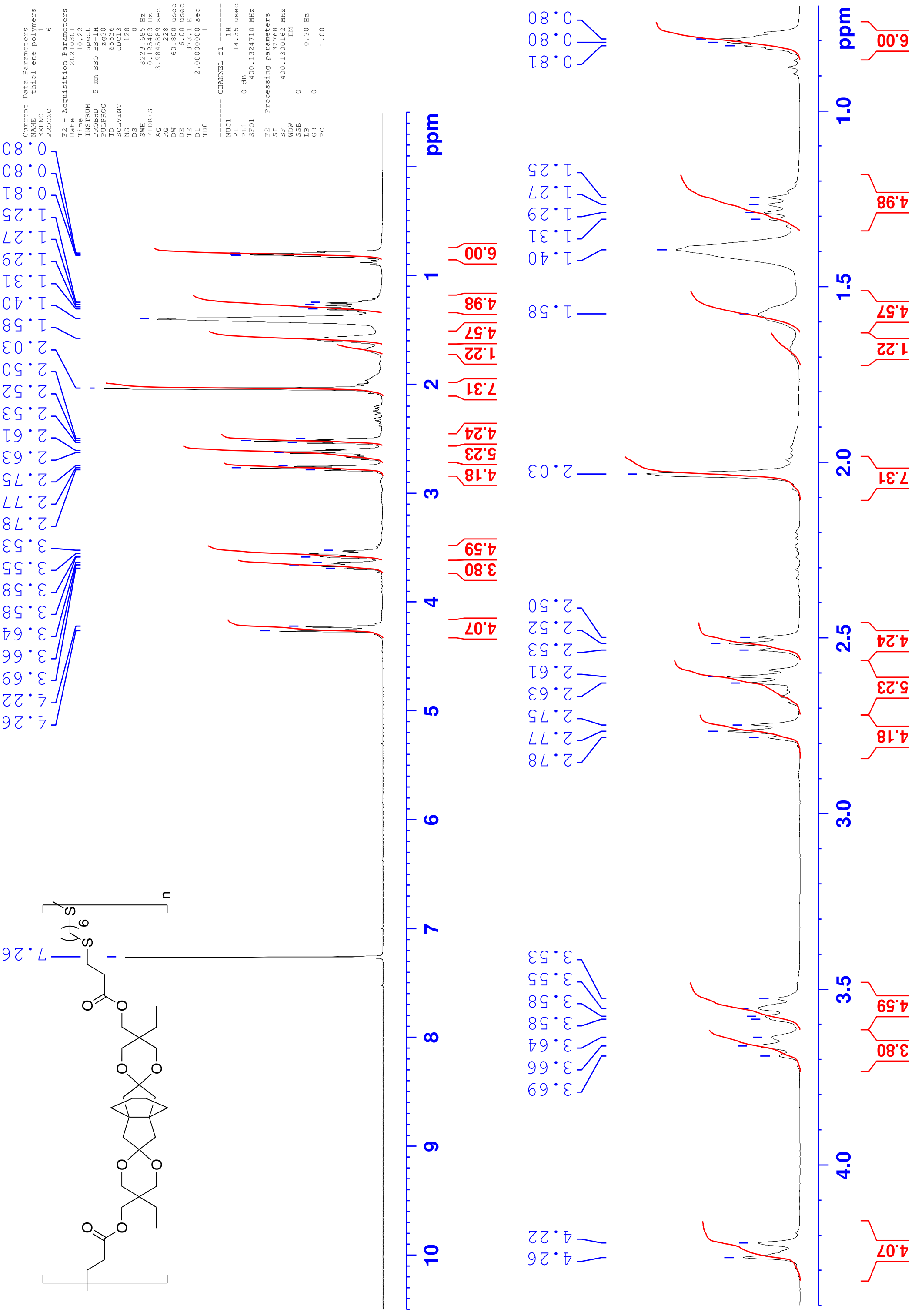

Fig. S22. *400 MHz ^{1}H spectrum of poly(tTa-HDT) in $CDCl_3$.*

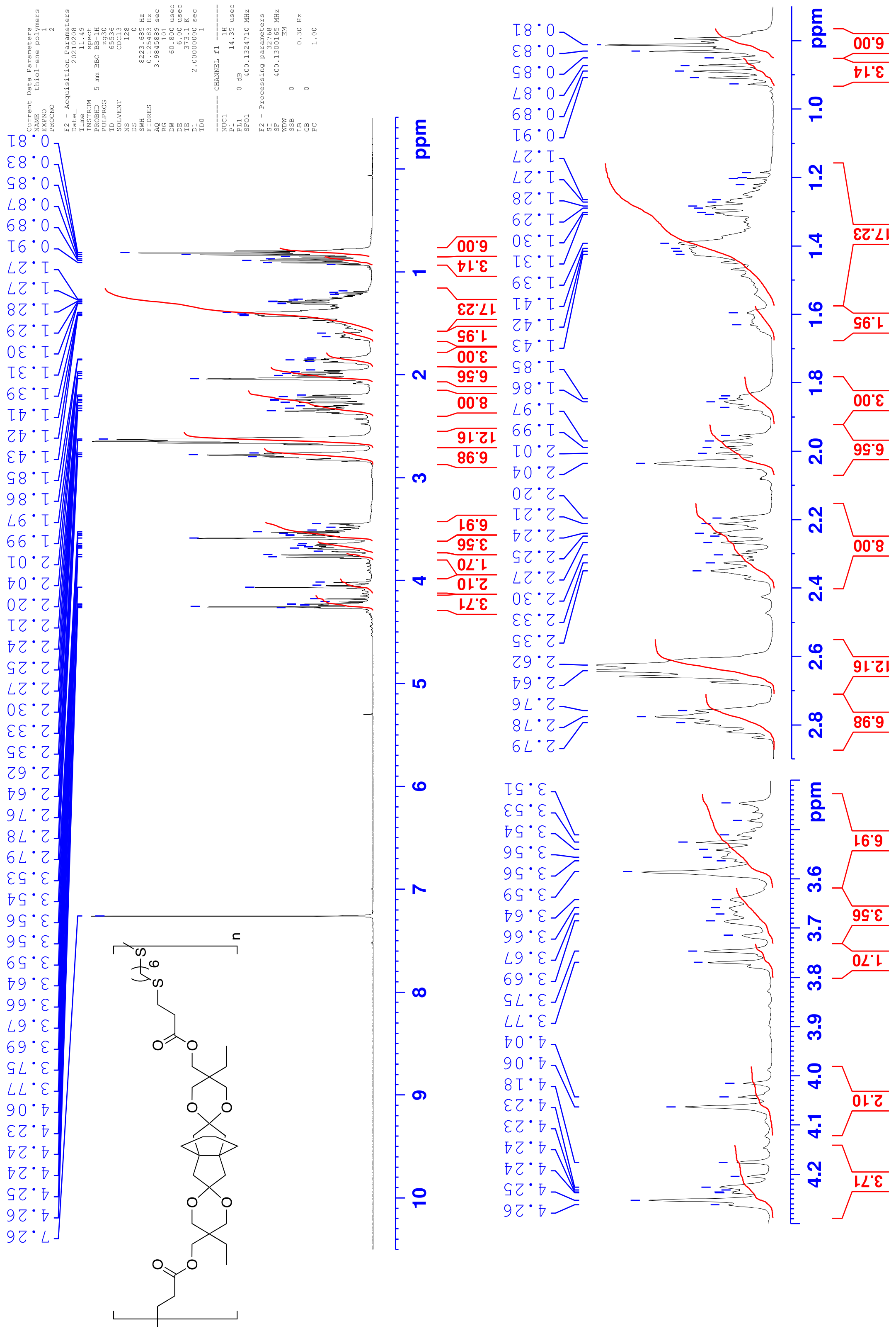

Fig. S23. *400 MHz* ^{1}H *spectrum of poly(tTa-PDT) in* $CDCl_3$.

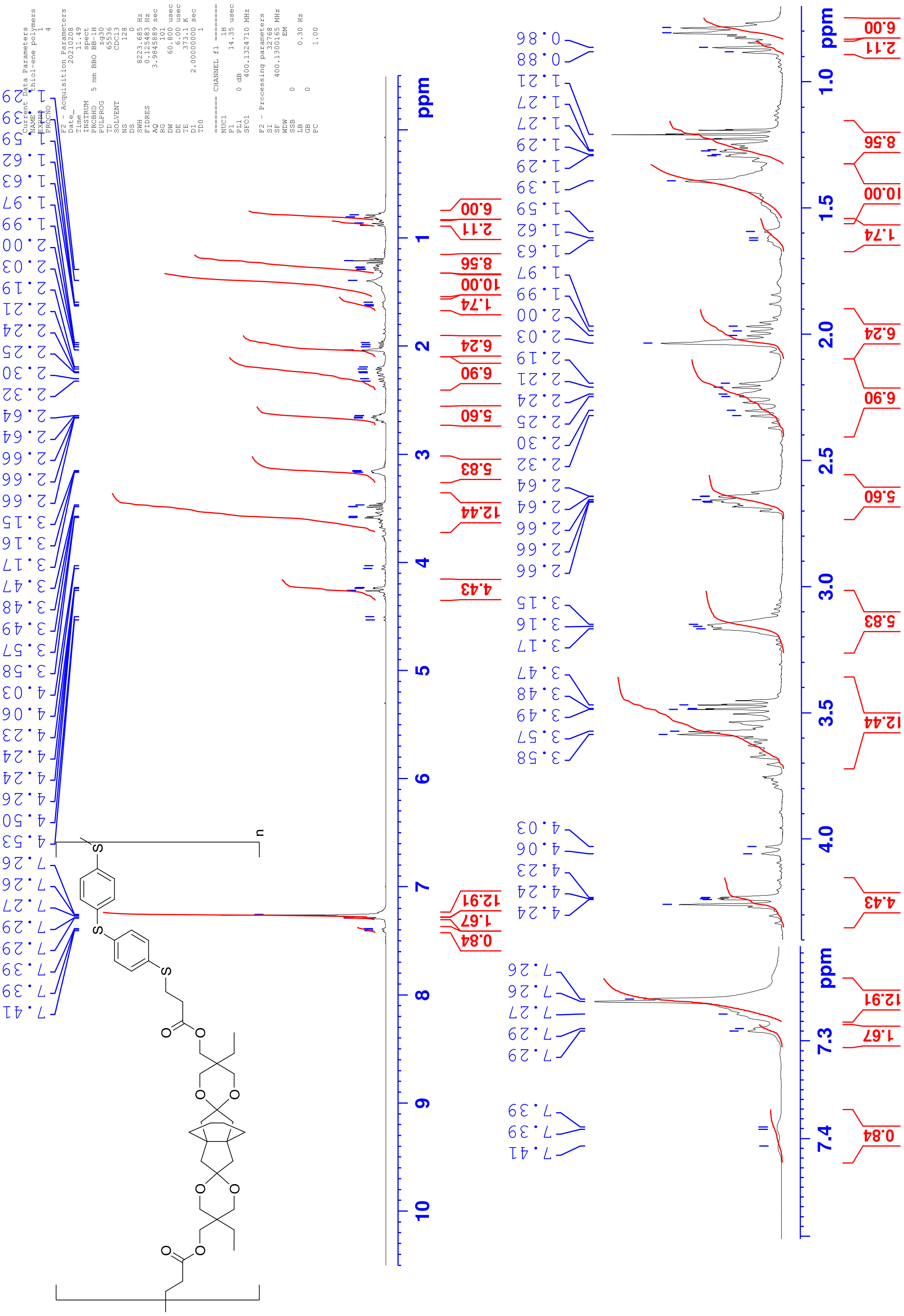

Fig. S24. *400 MHz* ^{1}H *spectrum of poly(tTa-TBBT) in* $CDCl_3$.

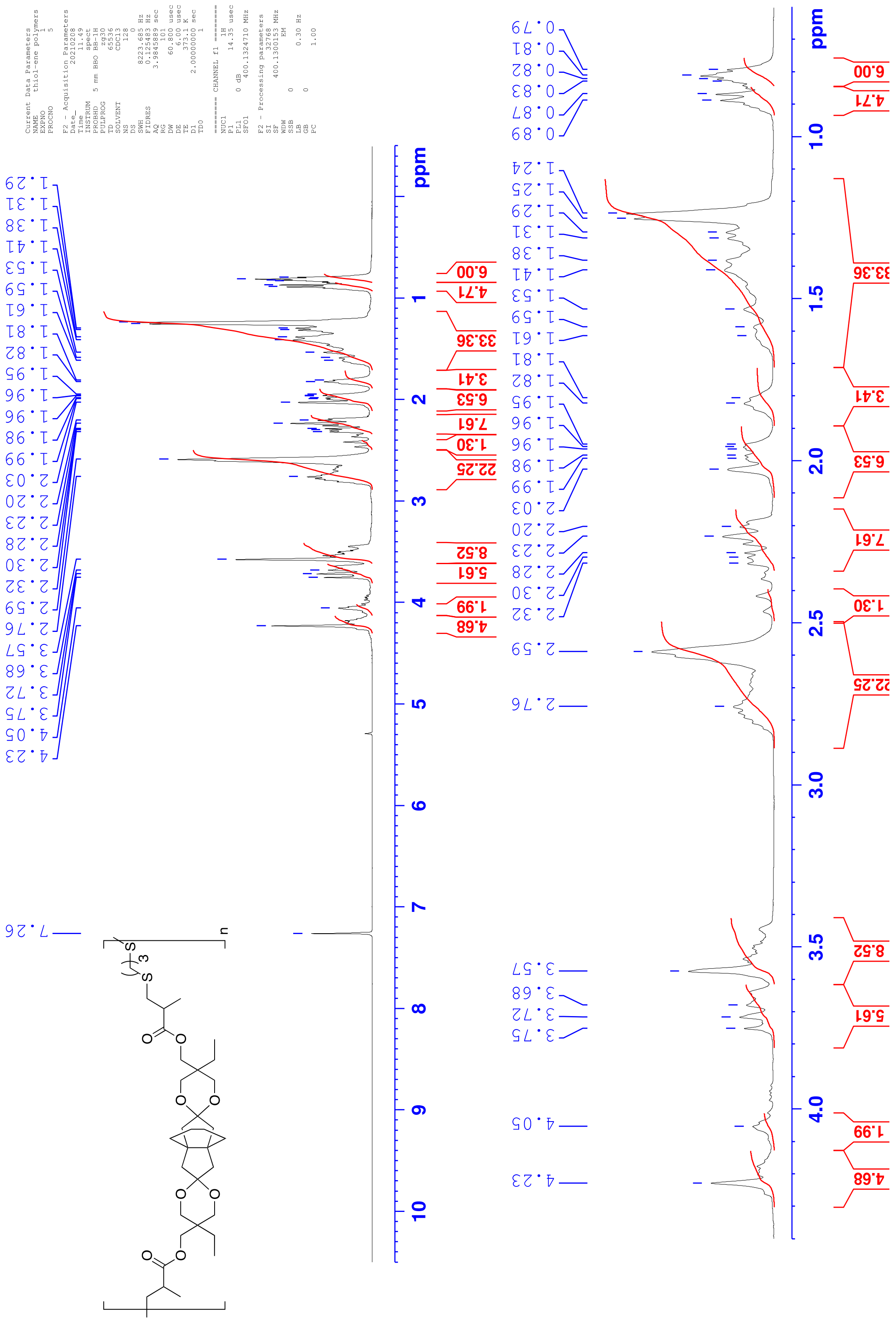

Fig. S25. *400 MHz* [1]*H spectrum of poly(tTma-PDT) in* $CDCl_3$.

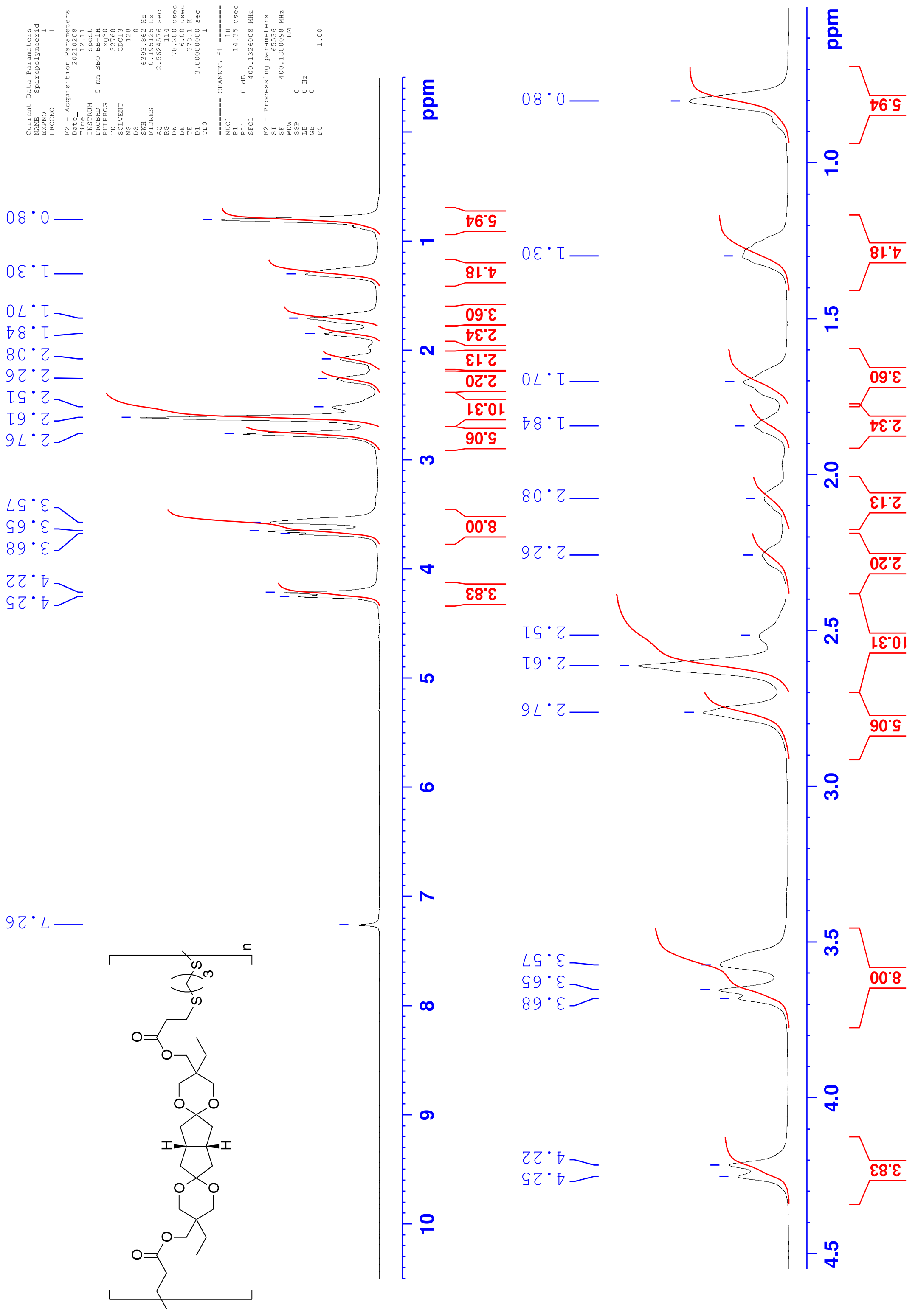

Fig. S26. *400 MHz* [1]*H spectrum of poly(tBa-PDT) in $CDCl_3$.*

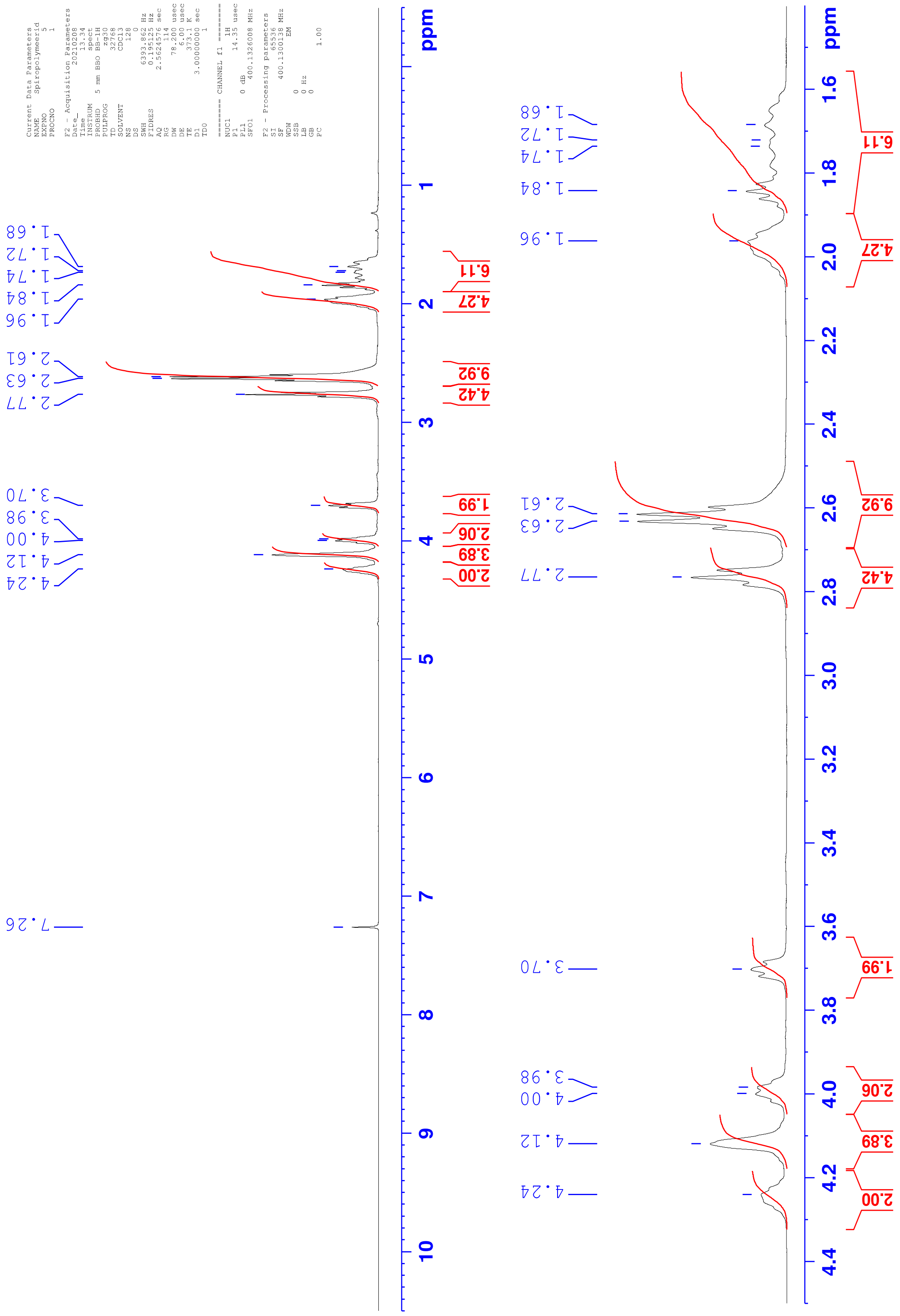

Fig. S27. *400 MHz ^{1}H spectrum of poly(gBa-PDT) in $CDCl_3$.*

Polymer characterization

SEC curves

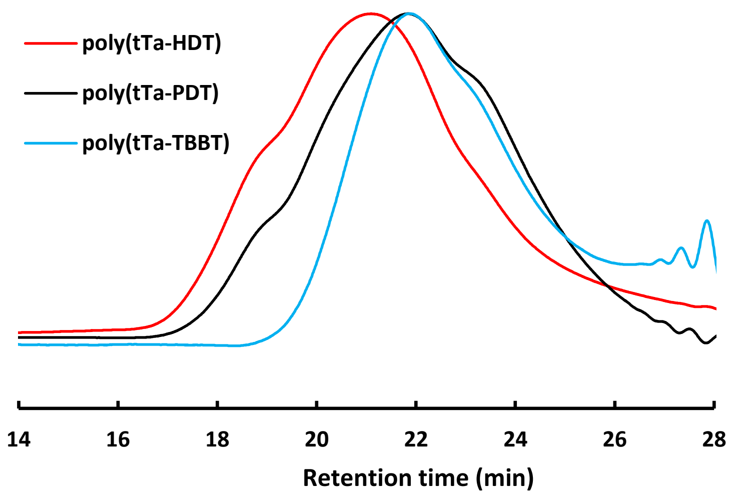

Fig. S28. *SEC curves of poly(tTa-HDT), poly(tTa-PDT) and poly(tTa-TBBT) measured in THF at 40°C.*

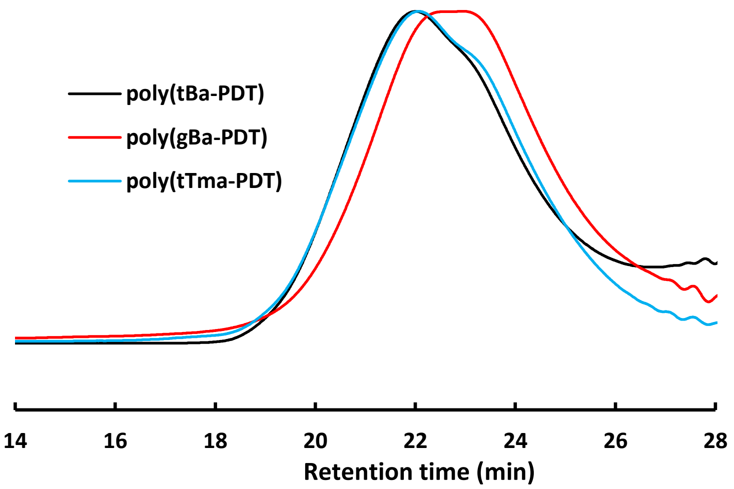

Fig. S29. *SEC curves of poly(tBa-PDT), poly(gBa-PDT) and poly(tTma-PDT) measured in THF at 40°C.*

DSC traces

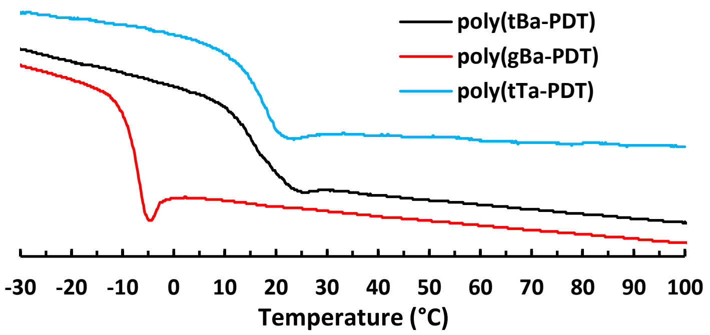

Fig. S30. *Second heating DSC traces of poly(tBa-PDT), poly(gBa-PDT) and poly(tTa-PDT).*

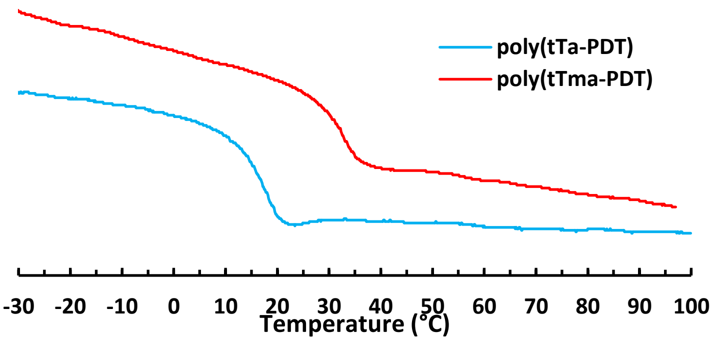

Fig. S31. *Second heating DSC traces of poly(tTa-PDT) and poly(tTma-PDT).*

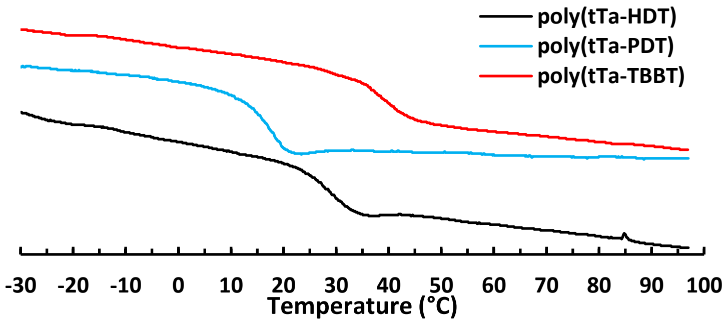

Fig. S32. *Second heating DSC traces of poly(tTa-HDT), poly(tTa-PDT) and poly(tTa-TBBT).*

TGA traces

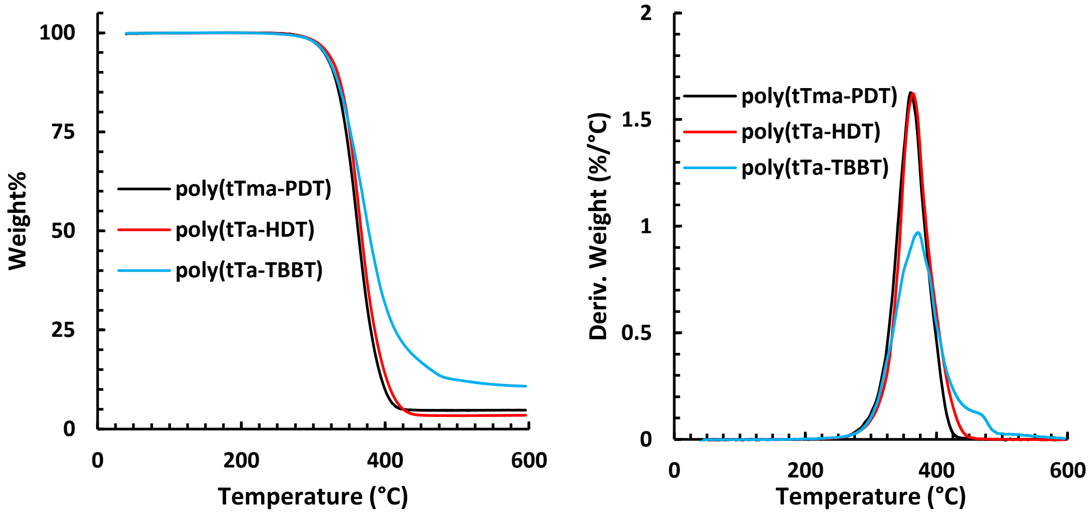

Fig. S33. *TGA traces (weight% and derivative) of poly(tTma-PDT), poly(tTa-HDT) and poly(tTa-TBBT), under N_2 atmosphere at 10 °C/min.*

Spirodiol hydrolytic stability studies

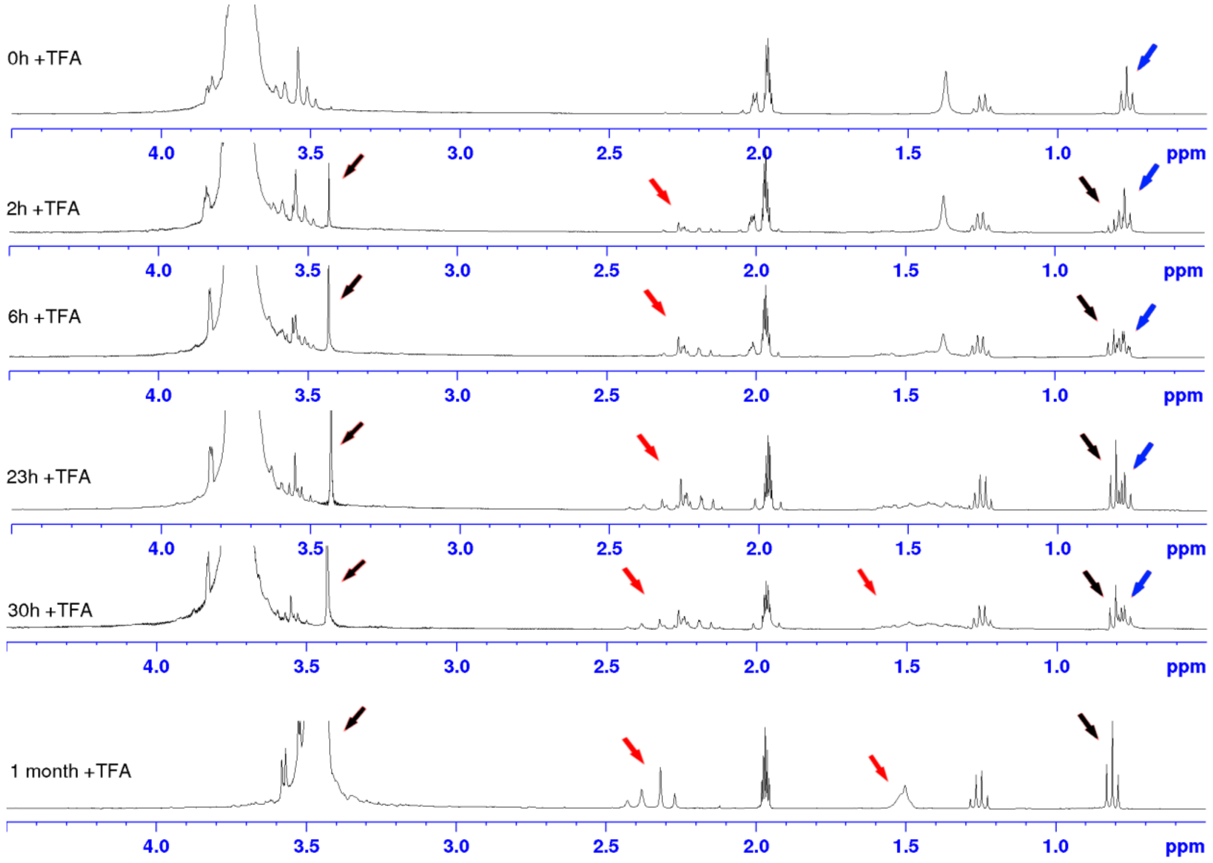

Fig. S34. *400 MHz ^{1}H NMR spectra recorded at different time intervals of **tT** hydrolysis in 10 mM TFA (aq.)/CD_3CN at room temperature. Red arrows show formation of diketone **T**, black arrows show formation of TMP. Blue arrows indicate the change in the methyl signal from **tT**.*

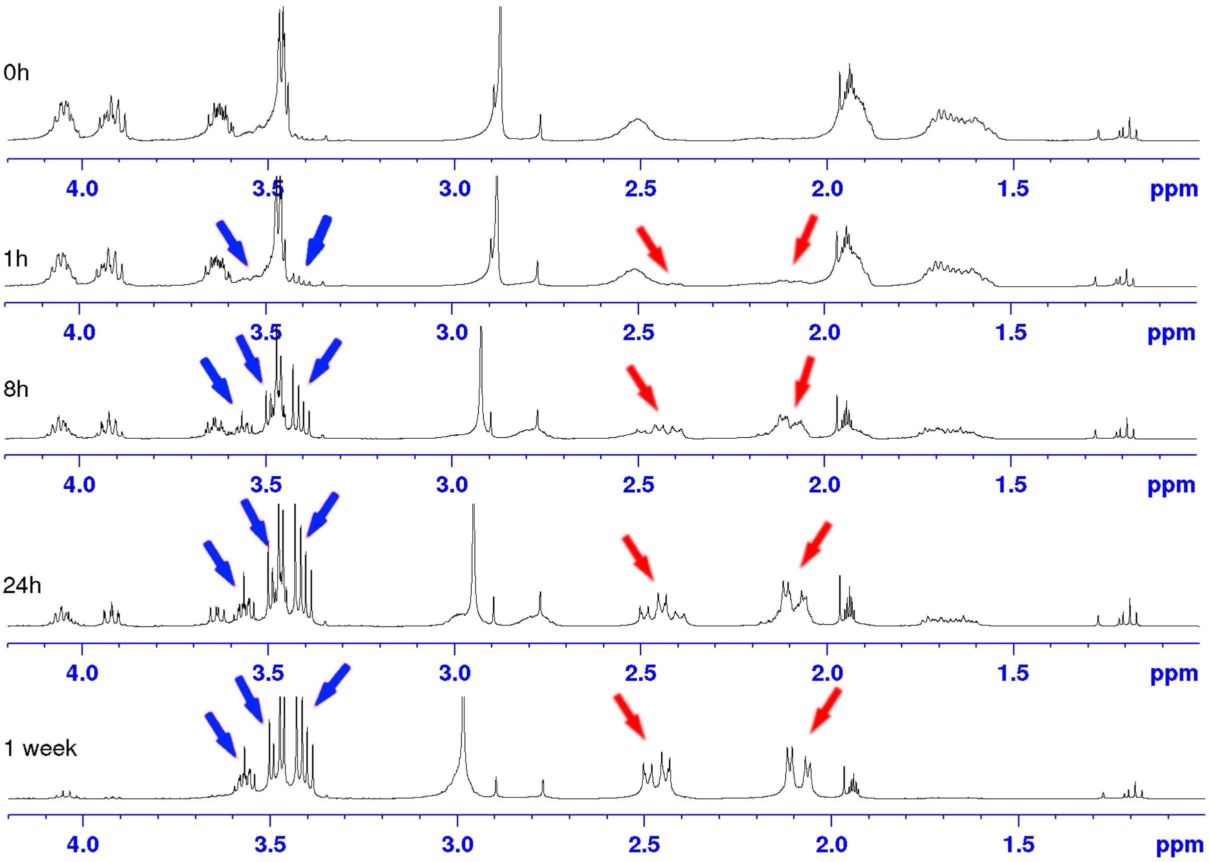

Fig. S35. *400 MHz ^{1}H NMR spectra recorded at different time intervals of **gB** hydrolysis in 10 mM TFA (aq.)/CD_3CN at room temperature. Red arrows indicate the formation of **B**, blue arrows show the formation of glycerol.*

Polymer hydrolysis experiments

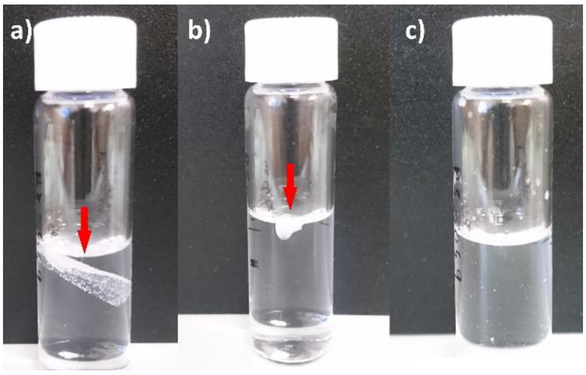

Fig. S36. *Degradation of poly(tTma-PDT) in 10% 1 M HCl (aq.)/90% acetone at 50 °C a) 0h, film submerged, b) 1h, film has coagulated, c) 2h, film has fully dissolved.*

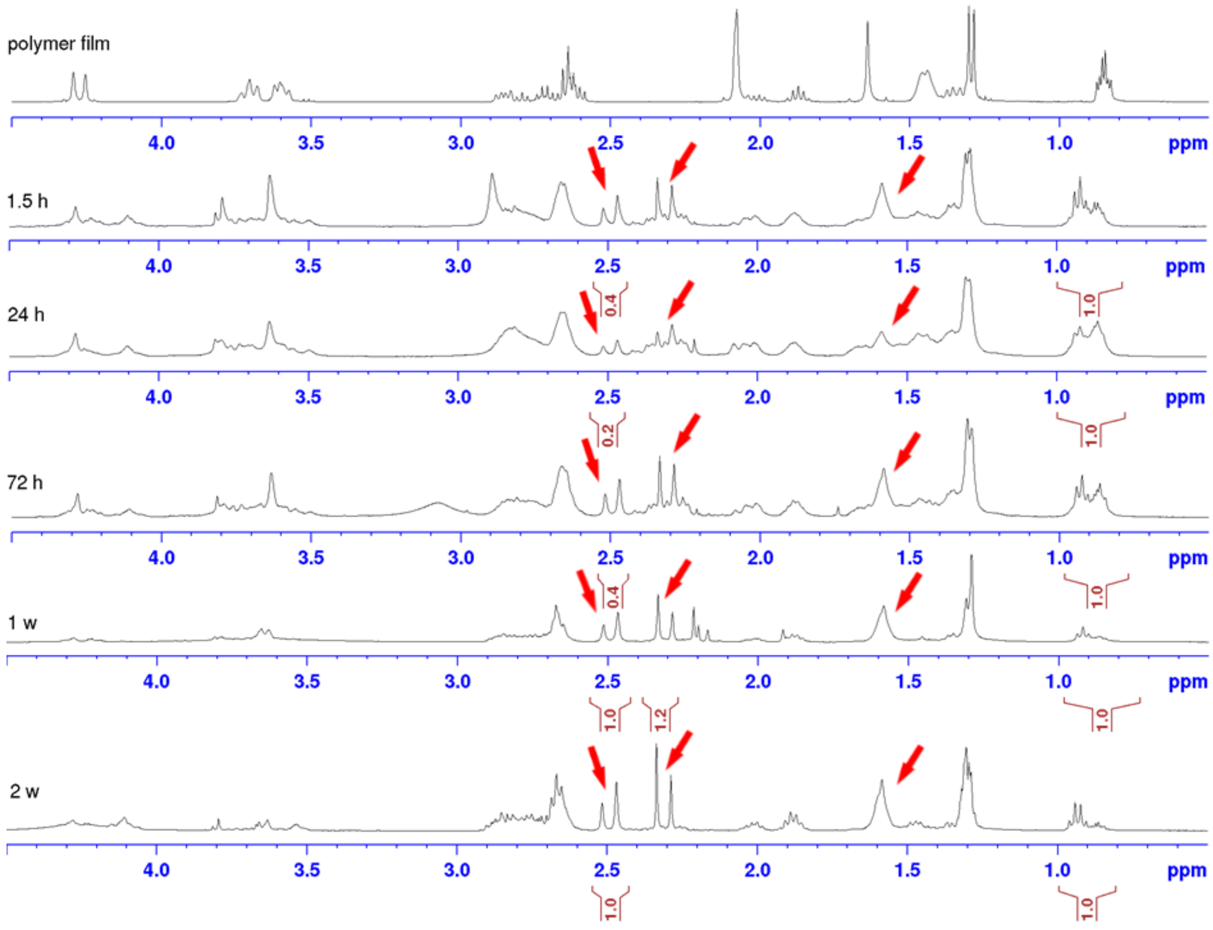

Fig. S37. *400 MHz ^{1}H NMR spectra recorded at regular intervals of poly(tTma-PDT) hydrolysis in 10% 1 M HCl (aq.)/90% acetone. Red arrows show forming diketone signals.*

Solubility of polymers

Table S2. Polymer solubility in a selection of solvents.

Polymer	H_2O	EtOAc	MeOH	THF	Et_2O	CH_3CN	$CHCl_3$	Toluene	DMSO
poly(tTa-HDT)	-	-	-	+	-	-	+	+	-
poly(tTa-PDT)	-	+	-	+	-	-	+	+	-
poly(tTa-TBBT)	-	-	-	+/-	-	-	+/-	+/-	-
poly(tTma-PDT)	-	+	-	+	-	-	+	+	-
poly(tBa-PDT)	-	-	-	+	-	-	+	+	-
poly(gBa-PDT)	-	- *	-	+	-	-	+	- *	-

*"+" indicates full solubility, "-" indicates insolubility, "+/-" indicates partial solubility. * cloudy*

References

(1) Kobayashi, S. O.; Simamura, O. An ESR Study on Pyramidal Inversion at the Tervalent Carbon Atom of 2-Methyl-1,3-Dioxolan-2-Yl Radical. *Chem. Lett.* **1973**, *2*, 699–702. https://doi.org/10.1246/cl.1973.699.

(2) Mihovilovic, M. D.; Müller, B.; Schulze, A.; Stanetty, P.; Kayser, M. M. An Enantiodivergent Trend in Microbial Baeyer−Villiger Oxidations of Functionalized Pentalenones by Recombinant Whole Cells Expressing Monooxygenases from Acinetobacter and Pseudomonas. *European J. Org. Chem.* **2003**, *2003*, 2243–2249. https://doi.org/10.1002/ejoc.200300023.

(3) Csunderlik, C.; Chirilă, T.; Bacaloglu, R. 1H NMR Investigations on the Conformation of the Spirodioxolanes Derived from Vicinal Diols. II—The Ketal of Cyclohexanone and Racemic 1,2-Propanediol. *Org. Magn. Reson.* **1982**, *18*, 153–156. https://doi.org/10.1002/mrc.1270180308.

(4) Eliel, E. L.; Rao, V. S.; Pietrusiewicz, K. M. Carbon-13 NMR Spectra of Saturated Heterocycles: VIII—Tetrahydrofurans and 1,3-Dioxolanes. *Org. Magn. Reson.* **1979**, *12*, 461–466. https://doi.org/10.1002/mrc.1270120805.

(5) Grant, D. M.; Dalling, D. K.; Johnson, L. F. Conformational Inversion Rates in the Dimethylcyclohexanes and in Some Cis-Decalins. *J. Am. Chem. Soc.* **1971**, *93*, 3678–3682. https://doi.org/10.1021/ja00744a021.

(6) Friebolin, H.; Faisst, W.; Schmid, G.; Kabuss, S. Konformative Beweglichkeit Flexibler Ringsysteme. Untersuchungen Mit Hilfe Der Protonenresonanz-Spektroskopie : IX. Mitteilung: 1,1-Dimethylcyclohexan Und Davon Abgelietete Verbindungen. *Tetrahedron Lett.* **1966**, 1317.

(7) Anet, F. A. L.; Bourn, A. J. R. Nuclear Magnetic Resonance Line-Shape and Double-Resonance Studies of Ring Inversion in Cyclohexane-D11. *J. Am. Chem. Soc.* **1967**, *89*, 760–768. https://doi.org/10.1021/ja00980a006.
